# Glycoprotein Hormones and Their Receptors Emerged at the Origin of Metazoans

**DOI:** 10.1093/gbe/evu118

**Published:** 2014-06-05

**Authors:** Graeme J. Roch, Nancy M. Sherwood

**Affiliations:** Department of Biology, University of Victoria, British Columbia, Canada

**Keywords:** cystine knot growth factor, bursicon, thyrostimulin, BMP antagonist, LGR evolution, GPH evolution

## Abstract

The cystine knot growth factor (CKGF) superfamily includes important secreted developmental regulators, including the families of transforming growth factor beta, nerve growth factor, platelet-derived growth factor, and the glycoprotein hormones (GPHs). The evolutionary origin of the GPHs and the related invertebrate bursicon hormone, and their characteristic receptors, contributes to an understanding of the endocrine system in metazoans. Using a sensitive search method with hidden Markov models, we identified homologs of the hormones and receptors, along with the closely related bone morphogenetic protein (BMP) antagonists in basal metazoans. In sponges and a comb jelly, cystine knot hormones (CKHs) with mixed features of GPHs, bursicon, and BMP antagonists were identified using primary sequence and phylogenetic analysis. Also, we identified potential receptors for these CKHs, leucine-rich repeat-containing G protein-coupled receptors (LGRs), in the same species. Cnidarians, such as the sea anemone, coral, and hydra, diverged later in metazoan evolution and appear to have duplicated and differentiated CKH-like peptides resulting in bursicon/GPH-like peptides and several BMP antagonists: Gremlin (Grem), sclerostin domain containing (SOSD), neuroblastoma suppressor of tumorigenicity 1 (NBL1), and Norrie disease protein. An expanded cnidarian LGR group also evolved, including receptors for GPH and bursicon. With the appearance of bilaterians, a separate GPH (thyrostimulin) along with bursicon and BMP antagonists were present. Synteny indicates that the GPHs, Grem, and SOSD have been maintained in a common gene neighborhood throughout much of metazoan evolution. The stable and highly conserved CKGFs are not identified in nonmetazoan organisms but are established with their receptors in the basal metazoans, becoming critical to growth, development, and regulation in all animals.

## Introduction

The evolutionary origin of hormones and receptors is an important question than can be addressed with genomic and phylogenetic analysis. A number of glycoprotein hormones (GPHs), including the pituitary hormones follicle-stimulating hormone (FSH), luteinizing hormone (LH), and thyroid-stimulating hormone (TSH), have a characteristic signature of cysteine amino acids from which three disulfide bonds form a knotted protein. These hormones are composed of two cystine knot glycoprotein subunits, α and β, forming a heterodimer (Bousfield et al. 2006; Bousfield and Dias 2011). The same α subunit, known as GPHα1, is common to FSH, LH, and TSH, whereas the β subunits (GPHβ1, GPHβ2, and GPHβ3) are distinct and define the specificity of the hormone. Human chorionic gonadotropin (hCG), a product of the placenta, is related to the pituitary hormones and likewise shares the common GPHα1 subunit but has a specific GPHβ4 subunit. Multiple functions are regulated by these vertebrate GPHs including control of reproduction by FSH and LH and metabolism by TSH (Hearn and Gomme 2000).

Examination of the human genome revealed a novel GPH related to pituitary hormones; this molecule was designated thyrostimulin (TS) because it bound the TSH receptor, leading to stimulation of thyroxine T4 (Nakabayashi et al. 2002). Like the pituitary hormones, TS forms a heterodimer with α and β subunits, designated as GPHα2 and GPHβ5 (Sudo et al. 2005). Mammalian TS has been localized in the pituitary and in several other organs including the gonads (Nakabayashi et al. 2002; Sun et al. 2010). To date, the functions of TS appear to be pleiotropic and include an increase in T4 and modulation of reproduction (Sudo et al. 2005; Okada et al. 2006; Sun et al. 2010). The TSs and pituitary GPHs shared a common ancestor in early vertebrates (Heyland et al. 2012). A single copy of each subunit gene (*gpha2* and *gphb5*) for invertebrate TS is thought to have duplicated during the two whole-genome duplications in ancestral vertebrates (Putnam et al. 2008; Dos Santos et al. 2011). Subsequent losses and modifications of the genes led to the retention of TS in vertebrates and the emergence of FSH, TSH, and LH/CG (Sudo et al. 2005; Roch et al. 2011). TS orthologs were also identified in invertebrates, including the nematode, fly, amphioxus, and tunicate (Vitt et al. 2001; Hsu et al. 2002; Campbell et al. 2004; Park et al. 2005; Holland et al. 2008; Dos Santos et al. 2009, 2011; Tando and Kubokawa 2009a, 2009b; Sellami et al. 2011). TS and the other pituitary GPHs were not found in basal animals including the sea anemone (Cnidaria), *Trichoplax* (Placozoa), a sponge (Porifera) (Dos Santos et al. 2009), or in nonmetazoan organisms such as yeast and mold (Vitt et al. 2001).

The pituitary GPHs and TS bind a family of related receptors, the leucine-rich repeat-containing G protein-coupled receptors (LGRs) (Park et al. 2005; Sudo et al. 2005; Van Hiel et al. 2012; Dong et al. 2013). These receptors have large ectodomains containing leucine-rich repeat (LRR) domains that confer ligand specificity. They also contain an extracellular “hinge” connected to the seven-transmembrane (7TM) domain. The first invertebrate LGR ortholog was identified and cloned from the sea anemone, a basal eumetazoan (Nothacker and Grimmelikhuijzen 1993; Vibede et al. 1998). Subsequently, LGR orthologs have been identified in a diversity of eumetazoan animals (Campbell et al. 2004; Vassart et al. 2004; Park et al. 2005; Freamat et al. 2006; Freamat and Sower 2008; Hauser et al. 2008; Van Loy et al. 2008; Van Hiel et al. 2012; Dong et al. 2013).

The GPHs are part of a larger superfamily composed of several other secreted proteins that contain the homologous cystine knot growth factor (CKGF) domain. There are two additional cysteine knot superfamilies, inhibitor cystine knots and cyclic cystine knots, which are not homologous to CKGFs (Craik et al. 2001). All three groups have two disulfide bonds that form a ring and a third disulfide bond that penetrates the ring, but only the growth factor knot uses cysteines I and IV of the knot as the penetrating bond. The other types of cystine knots are found in small molecules such as enzyme inhibitors or toxins in fungi, plants, or animals (Craik et al. 2001).

The CKGF superfamily includes the following six groups with the CKGF domain as the primary structural feature: 1) the GPH family, 2) bursicon hormone (Burs), 3) the bone morphogenetic protein (BMP) antagonist family, 4) the transforming growth factor beta (TGFβ) family, 5) the platelet-derived growth factor (PDGF) family, and 6) the nerve growth factor (NGF) family (Vitt et al. 2001; Luo et al. 2005; Mendive et al. 2005; Bousfield et al. 2006). Other CKGF domain-containing proteins (e.g., mucin, slit, jagged, hemolectin, chordin, and noggin) have multiple domains and were not compared with the single-domain families for this study (Vitt et al. 2001; Avsian-Kretchmer and Hsueh 2004; Lang et al. 2007). The bursicons are invertebrate hormones with two heterodimeric subunits that bind LGRs, similar to the GPHs (Luo et al. 2005; Mendive et al. 2005). The BMP antagonists prevent BMP ligands from binding their receptors (Rider and Mulloy 2010). Representatives of this group include gremlin (Grem), sclerostin, and sclerostin domain containing (SOSD), neuroblastoma suppressor of tumorigenicity 1 (NBL1, also known as DAN), and Norrie disease protein (NDP, also known as norrin) (Vitt et al. 2001; Avsian-Kretchmer and Hsueh 2004; Luo et al. 2005; Xu et al. 2012; Deng et al. 2013; Perbal 2013). Finally, members of the NGF, TGFβ, and PDGF families tend to bind various receptor kinases (Vitt et al. 2001; Bousfield et al. 2006).

The members of each family are of considerable interest because they regulate embryonic and organ development, growth, metabolism, and reproduction (Hearn and Gomme 2000; Mendive et al. 2005; Rider and Mulloy 2010). These secreted molecules are closely associated with the origin of multicellular animals, presumably for intercellular signaling including hormonal communication. This study considers the evolution of GPH and the other CKGF families with greatest homology, including bursicon and the BMP antagonists. We examine the origin of the hormones and their receptors to determine whether they emerged before the divergence of bilaterians from the basal metazoan lineages including cnidarians (sea anemone, coral, and hydra), placozoans, ctenophores (comb jellies), and poriferans (sponges).

## Results

### CKH-Like Peptides in the Most Basal Metazoans

**Fig. 1.—:**
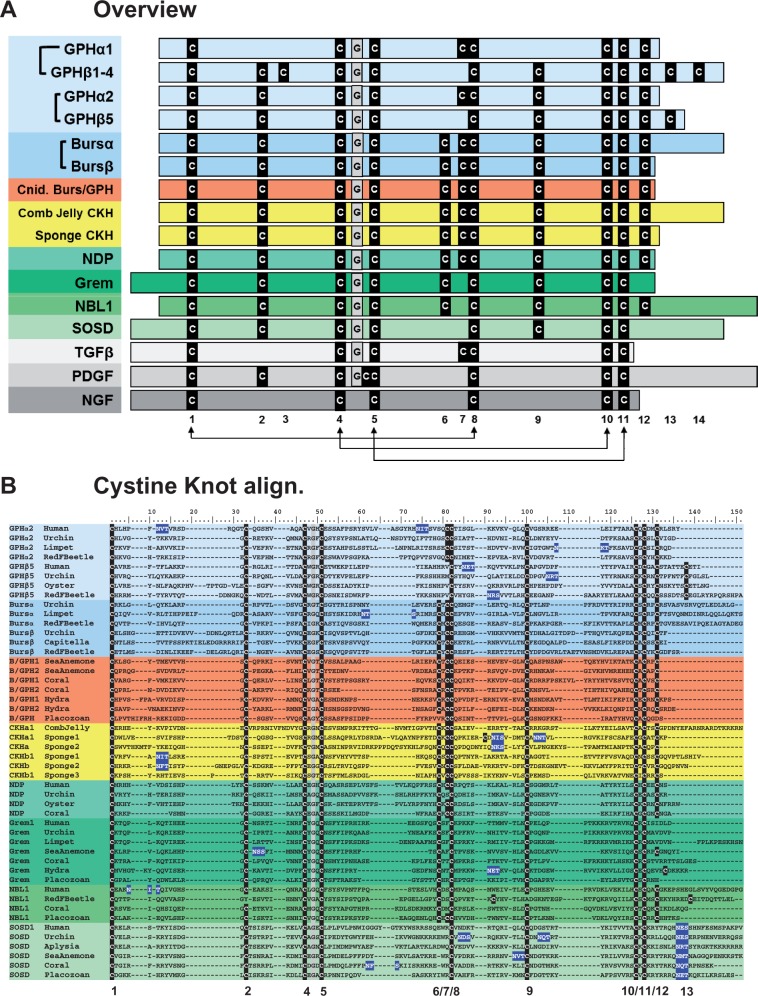

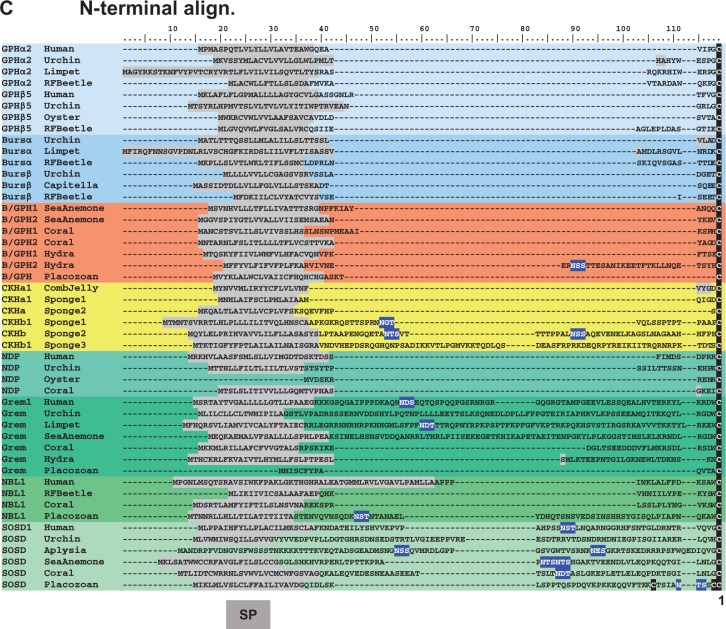
Alignment of superfamily cystine knot growth factor (CKGF) groups from metazoa. (*A*) An overview of superfamily members showing the core cysteines as a black box with a white “C.” The conserved glycine is in a gray box marked “G.” The six cysteines that form the knot are numbered below the diagram with the three conserved cystine knot disulfide bridges shown by connecting arrows. (*B*) Amino acid alignment of the cystine knot domain from selected families in (*A*), followed by partial C-terminal tail. (*C*) The N-terminal region of the proteins shown in (*B*). The predicted signal peptides (SP) are highlighted in gray and putative N-linked glycosylation sites are highlighted in dark blue. The individual families are shown by light blue bars for glycoprotein hormones (GPH): α1 (common subunit for LH, FSH, TSH, and hCG), β1-4 (subunits for LH, FSH, TSH, and hCG), α2, and β5 (subunits for thyrostimulin). Medium blue bars represent bursicon (Burs) hormones with α subunits and β subunits. The orange bar shows the cnidarian (cnid.) hormone that has features of both bursicon and GPHs (Burs/GPH). Placozoans also have putatie peptides with the Burs/GPH primary structure. The yellow bars show the comb jelly cystine knot hormone (CKH) and the sponge (three species) CKHs. The green bars represent different families known as BMP antagonists: Norrie disease protein (NDP), gremlin (Grem), neuroblastoma suppressor of tumorigenicity 1, and sclerostin domain containing (SOSD). The white bar shows the transforming growth factor β (TGFβ); the light gray bar is platelet derived growth factor (PDGF); and the dark gray bar is nerve growth factor (NGF).

An intensive search of sequence databases uncovered putative peptides from cnidarians, placozoans, comb jellies, and sponges with a signature CKGF structure. These peptides have the conserved cysteine residues necessary to form the cystine knot found in the superfamily, as seen in figure 1*A* (arrows indicate the intrachain disulfide bonds of the cystine knot). Although the six cysteines that form the knot are invariant, other cysteine residues within the cystine knot domain are variable among families. The NGF family is the most distinct, retaining only the cysteine residues necessary for the knot. The TGFβ and PDGF families also appear to have a distinct cysteine pattern from the rest (fig. 1*A*) and the peptides each produce specific gaps when aligned with the remaining families (supplementary fig. S1, Supplementary Material online). To date, only TGFβs have been reported in all four lineages that arose before bilaterians (Adamska et al. 2007; Pang et al. 2011).

We hypothesize that members of the remaining three CKGF families (GPHs, Burs, and the BMP antagonists) are more homologous to each other than to the others, with their own distinctive domain surrounding the cystine knot. This “cystine knot hormone” (CKH)-like domain was also found in the newly identified cnidarian, placozoan, comb jelly, and sponge sequences (fig. 1*A*). The alignments presented in figure 1*B* (the cystine knot and C-terminal region) and *C* (the N-terminal region of the peptides) demonstrate the similarities these cnidarian (highlighted in orange), comb jelly, and sponge peptides (in yellow) share with GPHs, Burs, and the BMP antagonists.

Sequence databases from three sponge species were interrogated, including two demosponges (Sponge1, *Ephydatia muelleri* and Sponge2, *Amphimedon queenslandica*) and a homoscleromorph sponge (Sponge3, *Oscarella carmela*). As well, sequence databases from the comb jelly *Mnemiopsis leidyi* were searched. Several putative sponge and comb jelly peptides were identified with a mixture of features found in the GPHs, Burs, and the BMP antagonists, and we have designated these peptides simply as CKHs. Within the cystine knot domain, the pattern of cysteine residues is identical in comb jelly CKH, sponge CKHs, cnidarian Burs/GPHs, and bilaterian Burs and NDP (fig. 1*A*). Unlike typical Grem and SOSD peptides, the sponge and comb jelly sequences have the conserved 7th and 12th cysteine residues (fig. 1*A* and *B*). Some of the sponge sequences have extended N-termini, similar to Grem and SOSD, with putative *N*-glycosylation sites such as SOSD (fig. 1*C*, in blue). The other sponge sequences and the comb jelly sequence have a short N-terminus composed primarily of the signal peptide (SP), similar to cnidarian Burs/GPH as well as bilaterian Burs, GPH, NDP, and NBL1. The C-terminal region is very short with the exception of the comb jelly CKH; this feature is not discriminative between the peptide families of interest. Comparing primary sequence, the putative sponge and comb jelly peptides present features common to the hormones (GPH and Burs) and the BMP antagonists.

Analyzed sequence databases from cnidarians contained representatives of two classes: The anthozoan sea anemone (*Nematostella vectensis*) and coral (*Acropora digitifera*), and the hydrozoan *Hydra magnipapillata* (also known as *H. vulgaris*). All three cnidarians have distinct gene models encoding the BMP antagonist Grem (fig. 1*B* and *C*). The sea anemone and coral also have orthologous gene models for SOSD, which we could not find within the hydra databases. Additionally, there is a probable coral ortholog of NBL1 and NDP, supported by primary structure and/or phylogeny. This implies that the cnidarian peptides have undergone more duplication and structural differentiation than the sponge and comb jelly CKHs (fig. 1*B* and *C*). The Grem and SOSD orthologs share characteristic primary sequence features not found in the GPHs or Burs, including an extended N-terminal region between the signal peptide and cystine knot domain (fig. 1*C*). Additionally, the cysteine pattern is modified in Grem, SOSD, and NBL1, with Grem and NBL1 missing the 7th conserved cysteine residue and SOSD missing the 6th and 7th cysteine (fig. 1*A* and *B*).

**Fig. 2.—:**
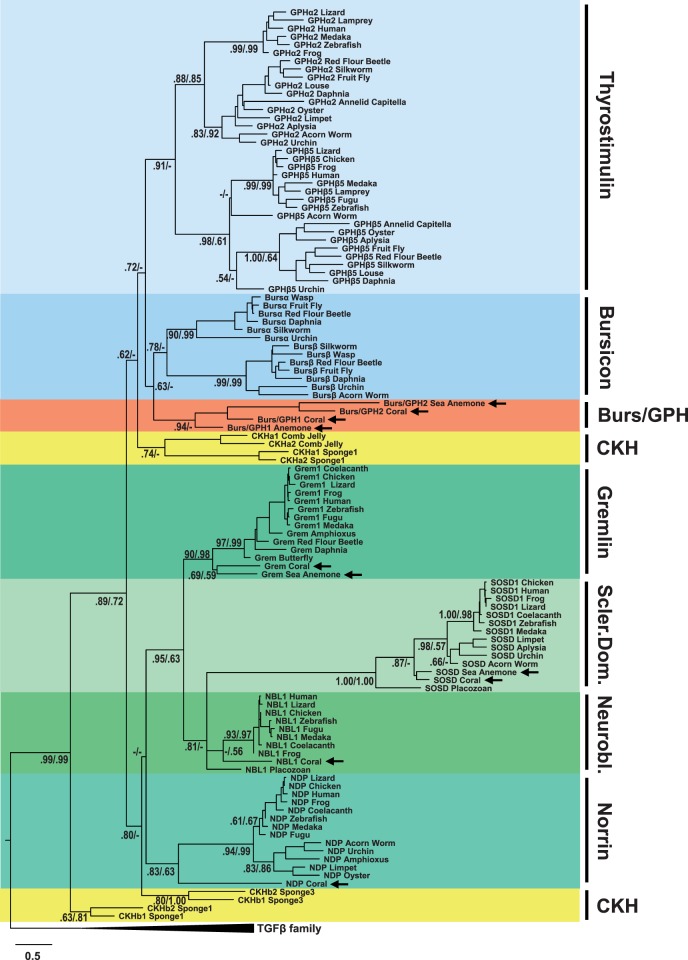
Phylogenetic tree of cystine knot hormones (CKHs) and homologs in basal metazoans. Members of the cystine knot growth factor (CKGF) superfamily were aligned, degapped, and trimmed to include only the cystine knot domain; a maximum-likelihood phylogeny is shown with SH-like supports and Bayesian posterior probabilities, respectively. All cnidarian sequences (sea anemone and coral) are identified with an adjacent arrow. The individual families are shown by light blue bars for glycoprotein hormone (GPH): α2 and β5 (subunits for thyrostimulin). Medium blue bars represent bursicon (Burs) hormones with α subunits and β subunits. The orange bar shows the cnidarian hormone that has features of both bursicon and GPHs (Burs/GPH). The yellow bars show the comb jelly CKH and the sponge (three species) CKHs. The green bars represent different families known as bone morphogenetic protein (BMP) antagonists: Norrie disease protein (NDP), gremlin (Grem), neuroblastoma suppressor of tumorigenicity 1 (NBL1), and sclerostin domain containing (SOSD). The scale bar denotes amino acid substitutions per site and the arrows indicate cnidarian sequences. The tree was rooted to a group of bilaterian, comb jelly, and sponge transforming growth factor β (TGFβ) family sequences. Maximum-likelihood trees were constructed with RAxML 7.7.9 under the PROTGAMMAIWAG model, and the best tree topology was selected with CONSEL 0.20 using the AU statistic. This tree is presented, with SH-like support values first and posterior probability support values second, from a separate Bayesian inference tree constructed with PhyloBayes MPI 1.4f under similar conditions (estimated gamma, WAG substitution model). Supplementary trees, the alignment, and the sources of sequences used are found in supplementary figure S2, Supplementary Material online.

Cnidarians also have gene models encoding peptides that we have designated as Burs/GPH, due to their phylogenetic position in figure 2. We could not determine whether these peptides were specific orthologs of the GPHs, Burs, or BMP antagonists by primary sequence alone. These peptides bear a cysteine pattern identical to that of Burs, the sponge CKHs, and NDP (fig. 1*A* and *B*). The Burs/GPH peptides have no additional cysteine residues after the 12th conserved cysteine, and typically no N-linked glycosylation sites; similar to Burs and NDP, and unlike GPH subunits (fig. 1*B* and *C*). As well, the N-terminal region that precedes the cystine knot domain is short in the cnidarian sequences, similar to Burs, the GPHs, and NDP (fig. 1*C*). Because of the ambiguous nature of these structural features, we used the more reliable phylogenetic position of these peptides as the basis for their classification as Burs/GPHs.

### Phylogenetic Analysis of CKGF Peptides

Phylogenetic analysis of the new sequences found in cnidarians, a placozoan, a comb jelly, and sponges was performed with corresponding bilaterian homologs, and a maximum-likelihood topology is presented in figure 2 with SH-like and posterior probability support values. The maximum-likelihood tree is found in supplementary figure S2*A*, Supplementary Material online, the Bayesian inference tree in supplementary figure S2*B*, Supplementary Material online, the alignment used to produce the phylogenies in supplementary figure S2*C*, Supplementary Material online, and the sequence information in supplementary figure S2*D*, Supplementary Material online. As shown, two major clades are formed that separate the GPHs and Burs from the BMP antagonists (Grem, SOSD, NDP, and NBL1). One group of sponge and comb jelly CKH peptides cluster basal to the Burs and GPH clades, albeit with weak statistical support owing to their short length and low degree of primary sequence conservation. This also prevented statistical inference using bootstrapped trees, and the topology from the Bayesian phylogeny was limited by several polytomies (supplementary fig. S2*B*, Supplementary Material online). The other group of sponge CKH peptides clustered separately, with the Sponge1 sequences basal to both the GPH/Burs and BMP antagonist clades, and the Sponge3 peptides immediately basal to the BMP antagonists (fig. 2). The group of cnidarian Burs/GPH peptides (highlighted in orange) clustered basal to the Burs clade. Care must be made in this interpretation, however, as separate analyses with different taxa and the removal of problematic peptide families, such as SOSD and NDP, resulted in topologies where the same cnidarian sequences would group basally to the GPH clade (not shown). Cnidarian sequences orthologous to Grem and SOSD (shown in green, with arrows) clustered with stronger support to their respective families, owing to a higher degree of sequence similarity shared between them (figs. 1*B*, 2*A*, and 2*B*).

### Phylogenetic Analysis of the Basal Metazoan Receptors: LGRs

**Fig. 3.—:**
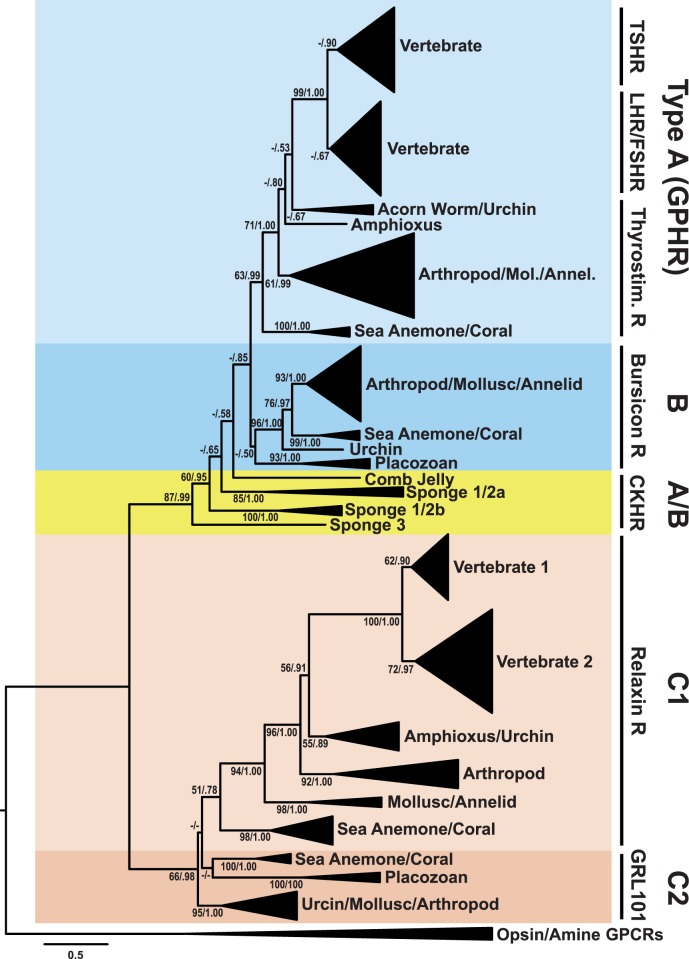
Phylogenetic tree of leucine-rich repeat-containing G protein-coupled receptors (LGRs) in basal metazoans. LGRs, including those of basal metazoans (cnidarians such as anemone and corals, comb jelly, and sponges) and homologs from several bilaterian species, were aligned, degapped, and trimmed to include only the seven transmembrane domain. A maximum-likelihood tree was constructed with RAxML 7.7.9 under the PROTGAMMALGF model. This tree is presented in compressed format, with bootstrap support values first and posterior probability support values second, from a separate Bayesian inference tree constructed with PhyloBayes MPI 1.4f under similar conditions (estimated gamma, LG substitution model). The scale bar denotes amino acid substitutions per site. The tree was rooted to a group of bilaterian opsins and neuropeptide G protein coupled receptors. Supplementary trees, the alignment, and the sources of sequences used are found in supplementary figure S3, Supplementary Material online. Annel., annelid; CKHR, cystine knot hormone receptor; FSHR, follicle-stimulating hormone receptor; GPHR, glycoprotein hormone receptor; GRL101, LGR with multiple LDLa domains; LHR, luteinizing hormone receptor; Mol., mollusc; Thyrostim. R, thyrostimulin receptor; TSHR, thyroid-stimulating hormone receptor.

Sequences homologous to the LGRs were also identified from sponge, comb jelly, placozoan, and cnidarian databases, to complement sea anemone and placozoan LGRs that have previously been identified and characterized (Nothacker and Grimmelikhuijzen 1993; Vibede et al. 1998; Van Hiel et al. 2012). Phylogenetic analysis of these sequences, presented in figure 3, provides evidence for the presence of potential CKH receptors in comb jelly and sponges (highlighted in yellow) and receptors for the Burs/GPH peptides in the cnidarians. As shown, sea anemone and coral LGR sequences cluster basal to the GPH receptor (GPHR) and bursicon receptor (BursR) clades, with reasonable bootstrap (>60) and posterior probability (>0.95) values. The GPHRs have been previously classified as “Type A” LGRs and the BursRs as “Type B” LGRs (Van Hiel et al. 2012). A placozoan receptor group also clusters basal to the BursR clade; however, its position is questionable owing to weak bootstrap (<50) and posterior probability (0.50) support. The comb jelly and sponge sequences (listed as Type A/B) all cluster basal to the entire GPHR/BursR receptor clade (Type A and B LGRs), with varying bootstrap and posterior probability support (fig. 3). There are no receptors for the BMP antagonists, as they bind BMPs to form heterodimers that prevent the stimulation of BMP receptors. The expanded maximum-likelihood tree is found in supplementary figure S3*A*, Supplementary Material online, the Bayesian inference tree in supplementary figure S3*B*, Supplementary Material online, the alignment used to produce them in supplementary figure S3*C*, Supplementary Material online, and sequence information in supplementary figure S3*D*, Supplementary Material online.

Sequences related to the vertebrate relaxin receptors, previously described as “Type C1” LGRs, have also been identified in the cnidarian databases. These receptors cluster basal to the bilaterian Type C1 LGRs, with moderate bootstrap (51) and posterior probability (0.78) support (fig. 3, dark pink). Additionally, a group of cnidarian and placozoan LGRs cluster basal to the rest of the Type C1 LGRs, and the cnidarian sequences contain multiple LDLa domains like the bilaterian GRL101 receptors (fig. 3, light pink). We have labeled these receptors as type GRL101 due to the presence of multiple LDLa domains, although they do not cluster basal to the bilaterian receptors, and are not well supported phylogenetically.

### Structural Features of the Cnidarian, Comb Jelly, and Sponge LGRs

**Fig. 4.—:**
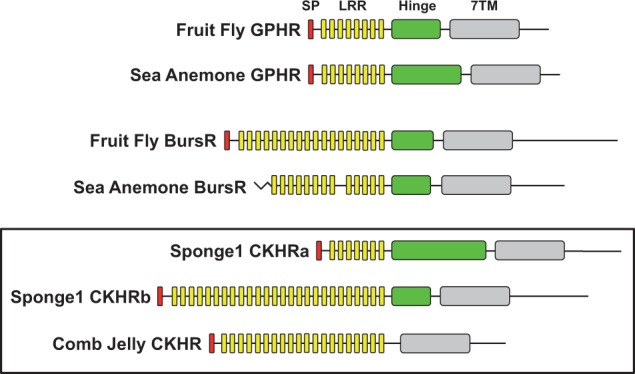
Primary sequence domains found in basal metazoan leucine-rich repeat-containing G protein-coupled receptors (LGRs). Domains common to the fruit fly glycoprotein hormone receptor (GPHR, also knows as thyrostimulin receptor) and fly bursicon receptor (BursR) are compared with receptors predicted from the sea anemone (*Nematostella vectensis*), comb jelly (*Mnemiopsis leidyi*), and a sponge (*Ephydatia muelleri*). The signal peptide (SP, red), leucine-rich repeats (LRRs, yellow), “hinge” (green) region, and seven transmembrane (7TM) domain (gray) are displayed, along with the C-terminal tail, approximately to scale. The sequences used, with these features highlighted, can be found in supplementary figure S4, Supplementary Material online.

Conserved domains in the primary structure of the cnidarian, comb jelly, and sponge LGRs were compared with those found in the fruit fly (*Drosophila melanogaster*) (fig. 4). As shown, there are four major structural features found in these receptors: A signal peptide followed by a variable number of LRRs, a “hinge” region with conserved motifs at each end, and the 7TM region. The cytoplasmic C-terminal tail that follows the 7TM was not considered in detail as it is poorly conserved. Motifs in figure 4 were deduced from the primary sequence of the LGRs shown in supplementary figure S4, Supplementary Material online.

A comparison of the sea anemone and fruit fly GPHRs and BursRs reveals that these receptor types also share a similar number of LRRs (8 for GPHR and 13–18 for BursR) and a hinge region of similar size. The sponge LGRs found in *E. muelleri* (Sponge1) have a disparate complement of LRRs, with 7 repeats found in one receptor and 26 repeats in the other. One of the sponge CKHRs also has a significantly enlarged hinge region compared with other Type A and B LGRs. The comb jelly CKHR has 20 LRRs and appears to lack the conserved hinge region found in all other LGRs to date, with a short region of sequence separating the LRRs from the 7TM region (fig. 4).

### Synteny of TS (GPHα2/GPHβ5) and BMP Antagonists throughout Metazoa

**Fig. 5.—:**
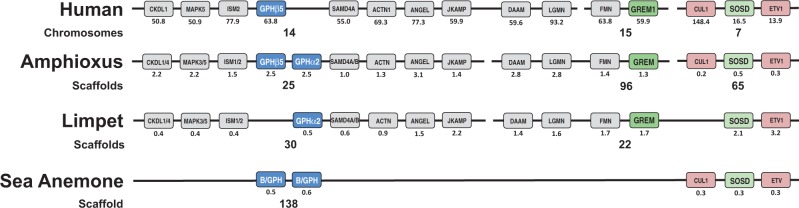
Synteny of genes encoding GPHα2/GPHβ5, gremlin (Grem), and sclerostin domain containing (SOSD) in human, amphioxus, limpet, and sea anemone. The diagram shows a representation of the common ortholog neighborhood shared by the genes encoding the thyrostimulin subunits (GPHα2/GPHβ5), shown in blue, and two bone morphogenetic protein (BMP) antagonists, Grem and SOSD, shown in green. Orthologs common to humans, amphioxus (*Branchiostoma floridae*, JGI assembly v2), the limpet (*Lottia gigantea*, JGI assembly v1), and the sea anemone (*Nematostella vectensis*, JI assembly v1) are shown in gray if they belong to the ancestral chordate linkage group 11, or pink if not. Genes are ordered according to how they are found on the limpet scaffolds, with their species-specific location in megabases (Mb) listed under the corresponding gene box. The orthologs of the genes encoding GPHα2/GPHβ5 found in the sea anemone are labeled B/GPH for bursicon/glycoprotein hormone. All gene boxes are labeled with their uppercase protein designations for legibility.

Conserved synteny is observed for the bilaterian locus that includes the genes encoding GPHα2 and GPHβ5 and the loci that include the genes encoding the BMP antagonists SOSD and Grem in the human, amphioxus, and limpet genomes (fig. 5). As shown, there are several orthologous genes (shown in gray) in proximity of the gene encoding human GPHβ5 that are conserved on single scaffolds found in the genomes of the amphioxus *Branchiostoma floridae* and the limpet *Lottia gigantea*. All of these genes belong to the conserved ancestral chordate linkage group 11 (CLG11), which is a paralogon (syntenic region resulting from whole-genome duplication) common to the genome of amphioxus and humans, presumably present in the ancestral chordate (Putnam et al. 2008). As well, a limpet scaffold that contains the genes encoding the SOSD and Grem peptides shares limited synteny with two separate scaffolds in amphioxus and three chromosomes in humans, including the genes encoding DAAM, Lgmn, and FMN (fig. 5). These genes are also from CLG11, whereas those flanking the gene encoding SOSD (the genes encoding Cul1 and ETV1) are from different CLGs, suggesting they may have separated genomically after the split of protostomes and deuterostomes. Orthologs of these flanking genes are also found in close proximity to the gene encoding SOSD in amphioxus and on the same chromosome in humans. They are also found alongside a gene encoding a SOSD ortholog on the same scaffold in the sea anemone that contains two Burs/GPH genes in close proximity. It should be noted that these two sea anemone Burs/GPH genes are an exception to most cnidarian, comb jelly, and sponge homologs. The genes encoding Bursα/Bursβ in the limpet are not found in the same locus.

## Discussion

### Basal Metazoans Have Peptides in the GPH, Bursicon, and BMP Antagonist Families

**Fig. 6.—:**
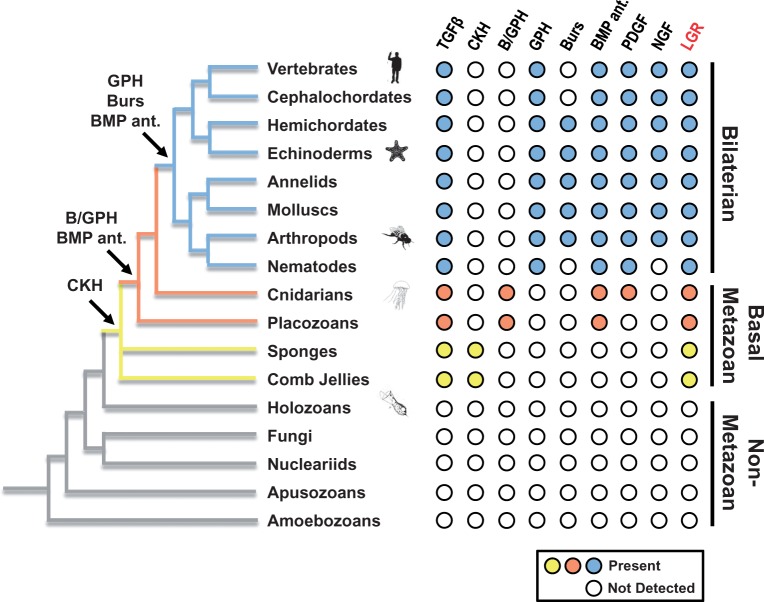
Detection of cystine knot growth factor (CKGF) and leucine-rich repeat-containing G protein-coupled receptor (LGR) homologs in eukaryotic groups. Members of the CKGF superfamily are listed across the top of the diagram, with dots indicating the presence (colored) or absence (white) of orthologs in each metazoan or nonmetazoan group. A representative phylogeny of the various metazoan and nonmetazoan groups is shown on the left, with branches color-coded according to the presence of cystine knot hormone (CKH, yellow), bursicon/glycoprotein hormone (B/GPH, orange) glycoprotein hormones (GPHs, blue), and bursicon (Burs, blue). Arrows indicate the ancestral node where the indicated peptides presumably originated. LGRs are listed in red at the top right of the diagram; they are the presumptive Type A, B, or A/B receptors for the aforementioned peptides. Metazoan and nonmetazoan databases were searched for the presence of homologs, and all major sequenced groups of amorpheans are displayed. Specific species databases that were searched are listed in supplementary table S1, Supplementary Material online. BMP ant., bone morphogenetic protein antagonist; NGF, nerve growth factor family; PDGF, platelet-derived growth factor family; TGFβ, transforming growth factor beta family.

Using a method of homology detection, HMMER, that is more sensitive than methods such as BLAST (Eddy 2009), we have uncovered putative peptides and receptors in cnidarian, placozoan, comb jelly, and sponge species. The peptides appear to be homologs of the GPHs, Burs, and the BMP antagonists Grem, SOSD, NDP, and NBL1 (fig. 1), whereas the receptors are homologous with LGRs, including the GPHRs, BursR, GRL101, and relaxin receptors. These peptides appear to have evolved at the origin of multicellular animals. The only other homologs of CKGFs identified to date in the most basal animals are TGFβ family members identified in a comb jelly (Pang et al. 2011) and a sponge (Adamska et al. 2007). We could not detect homologous CKGF peptides or LGRs in any of the nonmetazoan databases we investigated (fig. 6), including representatives from every major sequenced group of the unikonts (nonmetazoan holozoans, fungi, a nucleariid, an apusozoan, and amoebozoans; see supplementary table S1, Supplementary Material online, for details). Within the most basal of animals, we now have evidence suggesting a primitive endocrine/paracrine system that includes an ortholog (CKH) of the peptide hormones found in bilaterians.

The CKH sequences we found in a comb jelly and multiple sponge species have features common to the GPH, Burs, and BMP antagonist families (fig. 1). Although phylogenetic evidence places one group basal to the bursicon and GPH clades (fig. 2) with weak support, there is stronger evidence of cognate receptors for the comb jelly and sponge peptides (the “Type A/B” receptor sequences in fig. 3). We can only speculate as to whether these peptides function strictly as hormones or perform a hybrid role including BMP antagonism as well. The cnidarian peptides appear to have duplicated in similar fashion to the bilaterian peptides, with primary structure features more specific to Burs/GPH, Grem, NBL1, NDP, and SOSD. Although the LGR paralogs in cnidarians cluster with the GPHR and BursR clades separately (fig. 3), the homology of cnidarian Burs/GPH peptides is still ambiguous regarding Burs or GPH individually (figs. 1 and 2). These peptides have a cysteine pattern identical to that of the sponge CKHs, Burs, and NDP, indicating this may be the pattern found in ancestral CKH-like peptides.

Within the bilaterians, the genes encoding the TS subunits (GPHα2/GPHβ5) are almost always found in close proximity and the peptides form heterodimers (Dos Santos et al. 2009). This also appears to be the case for the bursicon subunits (Bursα/Bursβ) in certain insects (Mendive et al. 2005; Van Loy et al. 2008). Gene translocations do occur, as in the case of human and limpet GPH subunits (fig. 5). The genes encoding cnidarian, comb jelly, and sponge subunits are typically not found in proximity to duplicates (the sea anemone scaffold in fig. 5 is an exception). The maximum-likelihood phylogeny (fig. 2) suggests that the genes encoding GPH subunits share a more recent gene ancestor with each other than they do with those encoding Burs subunits. Therefore, the heterodimeric nature of the bilaterian peptides is the likely result of independent duplications for the genes encoding GPH and Burs subunits. Accordingly, we cannot say for certain whether the comb jelly CKH, sponge CKHs, and cnidarian Burs/GPH peptides are heterodimers or homodimers, but the latter seems more probable, and was likely the ancestral state.

### Basal Metazoans Have LGRs from the Same Families as Bilaterians

The LGR sequences found in both the cnidarian and sponge databases provide a clear picture for the presence of distinct hormone signaling systems in these basal animals. The phylogenetic evidence presented in figure 3 indicates that the Type A/B receptors present in the comb jelly and three sponge species analyzed are basal to both the GPH and Burs receptors. Structural analysis of the sea anemone and fruit fly GPHRs and BursRs (fig. 4) supports the phylogeny of the receptors shown in figure 3. The sea anemone GPHR and BursR sequences have similar features compared with their fruit fly orthologs, including the number of LRRs in the ligand-binding domain. The comb jelly and sponge LGRs have LRR domains with a variable number of repeats (7–26) and hinge domains that can be quite extended or nonexistent. This suggests a simplified CKH-receptor system emerged in the early animals before a duplication event that resulted in the GPHR (Type A) and BursR (Type B) families in the cnidarians and bilaterians.

In addition to the LGRs that bind GPHs and Burs, there are also orthologs of the vertebrate relaxin receptors (Type C1) and GRL101s (Type C2) found in sea anemones, corals, and placozoans. Although orthologs of the vertebrate relaxins have only been identified in invertebrate deuterostomes like the echinoderm starfish (Mita et al. 2009), the presence of these receptors suggests the possibility that insulin-like peptides found in protostomes and cnidarians might also bind their Type C LGRs. It could also be a separate peptide family found in invertebrates, as the only similarity between the CKH-like hormones and relaxins is the presence of intramolecular disulfide bridges, which many other secreted peptides also have. In any case, the presence of Type A, B, and C LGRs in these basal animals indicates that at least a primitive intercellular signaling system utilizing the LGRs was present in early metazoan evolution.

Indeed, the evolution of metazoans coincided with an expansion of GPCR families from the complement found in their nonmetazoan ancestor to those that would bind multiple types of signaling peptides such as the CKH-like family. The LGRs belong to the Rhodopsin (or Class A) family of GPCRs. Homologs from this family can be found in several nonmetazoan eukaryotic genomes not only in groups closely related to the metazoans such as choanoflagellates and fungi, but also from alveolates and heterokonts, groups separated far across the eukaryotic spectrum (Krishnan et al. 2012; de Mendoza et al. 2014). These include homologs of the opsin GPCRs and adrenergic receptors. Upon the evolution of multicellularity within the metazoan ancestors, the Rhodopsin family expanded with new receptor types fused to novel N-terminal domains like the LRRs, resulting in the metazoan-specific LGR receptors found in all types of animals (de Mendoza et al. 2014).

### Synteny of GPH Subunits and BMP Antagonists Suggests Their Conserved Evolution

An analysis of the syntenic environment surrounding the genes encoding GPHα2/GPHβ and those encoding Grem and SOSD in the genomes of the human, amphioxus, limpet, and sea anemone suggests a common environment from which a single ancestral CKH peptide may have evolved. This supports our hypothesis that these genes duplicated tandemly in an early eumetazoan from a common ancestral gene encoding a CKH. Although this syntenic relationship was restricted to these few species, they have been demonstrated to retain a large amount of microsynteny in larger scale analyses (Irimia et al. 2012; Simakov et al. 2013). We do not know why these genes are maintained in a syntenic locus; however, it is possible that they are subject to a common cis-regulatory element that has been retained across many eumetazoan taxa. This is supported by the recent discovery that cis-regulatory associations for many critical developmental regulators such as Grem have been conserved throughout animals (Irimia et al. 2012).

### Conclusions: The Origin of the CKGF Superfamily

The origin of the CKGF superfamily and LGRs in early metazoans follows a pattern of cell signaling gene families that arose alongside animal multicellularity. Many intercellular signaling systems had most of their cytosolic machinery present in the common ancestor of metazoans, choanoflagellates, and filastereans (holozoans) (King et al. 2008; Srivastava et al. 2010; Suga et al. 2013). In early metazoans, these systems added ligands and receptors; examples include the conserved Wnt and Notch–Delta systems (Srivastava et al. 2010; Ryan et al. 2013; Riesgo et al. 2014). Similar to these families, a simple system of CKGFs and their cognate receptors evolved in the ancestral metazoans and this system remains in the sponges and comb jellies. With the rise of the eumetazoans, the repertoire of the superfamily expanded into several families of endocrine ligands, growth factors, and their antagonists.

We can begin to piece together the evolutionary history of the entire superfamily of CKGF peptides with a focus on the extended family which includes the GPHs, Burs, and the BMP antagonists. As suggested by figure 6, it is likely that an ancestral gene in an early metazoan duplicated into those encoding TGFβ and CKH, and this is still the case in comb jellies and sponges. The PDGF family appears to have originated from a CKGF duplication in a common ancestor of cnidarians and bilaterians. We could not find NGF homologs in the basal metazoans, suggesting a duplication in the ancestor of bilaterians produced this family. By the time of the divergence of the cnidarians from the lineage that would become the bilaterians, distinct Burs/GPH and BMP antagonist peptides had appeared. Further duplications produced the GPHα2/GPHβ5 and Bursα/Bursβ heterodimers in early bilaterians. Finally, in the jawed vertebrates, the genes encoding the TS subunits had duplicated to produce the pituitary GPH (TSH/FSH/LH) subunit genes.

## Materials and Methods

### Data Mining for GPH Homologs and LGRs

To determine the presence of GPH homologs and LGRs within the nonbilaterian animal lineages and nonmetazoan eukaryotes, custom hidden Markov models (HMMs) were built using a diverse set of confirmed and predicted bilaterian protein sequences that were initially aligned by MSAprobs (Liu et al. 2010) and then constructed with the hmmbuild program of HMMER 3.0 (http://hmmer.janelia.org/, last accessed May 26, 2014). These HMMs were then used to search protein and translated nucleotide databases from NCBI (http://ncbi.nlm.nih.gov/, last accessed May 26, 2014) and a variety of species-specific databases: 1) cnidarians, including the sea anemone *N. vectensis* (http://genome.jgi-psf.org/Nemve1/Nemve1.home.html, last accessed May 26, 2014), the coral *Ac. digitifera* (http://marinegenomics.oist.jp/genomes/, last accessed May 26, 2014), and the hydrazoan *H. magnipapillata* (http://www.ncbi.nlm.nih.gov/assembly/GCA_000004095.1, last accessed May 26, 2014); 2) the placozoan *Trichoplax adhaerens* (http://genome.jgi-psf.org/Triad1/Triad1.home.html, last accessed May 26, 2014); 3) the ctenophore comb jelly *M. leidyi* (http://research.nhgri.nih.gov/mnemiopsis/, last accessed May 26, 2014); and 4) poriferans including the demosponges *A. queenslandica* (http://metazoa.ensembl.org/Amphimedon_queenslandica/Info/Index, last accessed May 26, 2014) and *E. muelleri*, and the homoscleromorph sponge *O. carmela* (http://compagen.zoologie.uni-kiel.de/datasets.html, last accessed May 26, 2014). Putative homologs that had the conserved cysteines found in bilaterian CKGF superfamily proteins (fig. 1) were then subjected to BLASTp analysis (Altschul et al. 1997) against the nonredundant protein database at NCBI to see if their best hits were indeed homologs of these peptides.

### Alignments

Peptide sequences with features similar to the GPHs, Burs, and the BMP antagonists were aligned from several bilaterian species along with sea anemone (*N. vectensis*), coral (*Ac. digitifera*), hydra (*H. magnipapillata*), comb jelly (*M. leidyi*), and three sponges (Sponge1, *E. muelleri*; Sponge2, *A. queenslandica*; and Sponge3, *O. carmela*). The cystine knot domain (fig. 1*B*) was aligned with MAFFT v7.130 (Katoh and Standley 2013) using the “linsi” strategy with default options. The N-terminal region (fig. 1*C*) was aligned separately with MUSCLE v3.8.31 (Edgar 2004) using default options. Signal peptides and N-linked glycosylation sites were predicted using the Technical University of Denmark Center for Biological Sequence Analysis web servers (http://www.cbs.dtu.dk/services, last accessed May 26, 2014). The sequences used to generate these alignments are found in supplementary figure 2*D*, Supplementary Material online.

### Phylogenetic Analysis

Phylogenetic analysis was conducted using similar methods for both cystine knot peptides and the LGRs. Receptor sequences were first trimmed to their 7TM region using the “–trim” command in the HMMER program hmmalign and an HMM model for the TM region found in rhodopsin-family GPCRs (http://pfam.sanger.ac.uk/family/PF00001/hmm, last accessed May 26, 2014). Peptide and receptor sequences were aligned using MSAProbs (Liu et al. 2010) with default values and degapped using BMGE (Criscuolo and Gribaldo 2010) with a BLOSUM30 matrix, entropy score cutoff of 1 (“-h 1”) and block size of 1 (“-b 1”). The alignments are provided in supplementary figures S2*C* (peptides) and S3*C* (receptors), Supplementary Material online. ProtTest 3 (Abascal et al. 2005) was used to determine the best substitution model for further analysis, which was WAG+I+G (estimated gamma value, estimated invariant sites, and model-based equilibrium frequencies) for the peptides and LG+G+F (estimated gamma, no invariant sites, and empirical equilibrium frequencies). Maximum-likelihood analysis and Bayesian analysis were conducted on the Western Canadian Research Grid (http://www.westgrid.ca/, last accessed May 26, 2014). Maximum-likelihood analysis was performed with RAxML 7.7.9 (Stamatakis 2006) for both the peptide and receptor alignments. The receptor tree was produced under the “rapid bootstrap analysis and search for best-scoring ML tree in one program run” option (“-f a”), with the substitution model PROTGAMMALGF. This topology was then used for the tree figure presented in the Results. Support values were generated from the rapid bootstraps for the receptor tree and posterior probability values from a separate tree generated in PhyloBayes MPI 1.4f (Lartillot et al. 2013). The Bayesian tree was constructed using the same constraints as the maximum-likelihood tree (LG substitution model, four gamma categories), with two chains that had the first 5,000 trees discarded as burn-in, and the next 20,000 trees sampled at every 10th tree (2,000 trees total per chain). To determine the optimal topology for the peptide tree, 100 random trees were generated in RAxML under the substitution model PROTGAMMAIWAG and the resulting trees, and site likelihoods were analyzed in CONSEL 0.20 (Shimodaira and Hasegawa 2001) to determine the topology with the best value from the approximately unbiased (AU) test. Support values were generated from the SH-like values calculated for the optimal topology in RAxML (“f -J”) and posterior probability values from a separate tree generated in PhyloBayes MPI 1.4f (Lartillot et al. 2013). The Bayesian tree was constructed using the same constraints as the maximum-likelihood tree (WAG substitution model, four gamma categories), with two chains that had the first 20,000 trees discarded as burn-in and the next 80,000 trees sampled at every 10th tree (8,000 trees total per chain). The resulting maximum-likelihood trees were compressed, and the branches were reordered in FigTree (http://tree.bio.ed.ac.uk/software/figtree/, last accessed May 26, 2014). The sequences used to construct the trees are listed in supplementary figures S2*D* and S3*D*, Supplementary Material online.

### LGR Features

To determine the features found in the newly discovered comb jelly and sponge LGR sequences, predicted amino acid sequences from the cnidarian sea anemone (*N. vectensis*), comb jelly (*M. leidyi*) and sponge *E. muelleri* (Sponge1) were analyzed for structural motifs using ScanProsite (de Castro et al. 2006) and LRRfinder (http://www.lrrfinder.com/, last accessed May 26, 2014) to compare them with the fruit fly (*D. melanogaster*) GPHR and BursR. LRRs were defined by the LxxLxLxxN/C motif where possible. Sequences from this figure are shown in supplementary figure S4, Supplementary Material online.

### Synteny Analysis

Microsynteny analysis of the amphioxus (*B. floridae*) locus encoding GPHα2/GPHβ5, SOSD, and Grem peptides was performed against the human genome (http://www.ncbi.nlm.nih.gov/genome/guide/human/, last accessed May 26, 2014), the limpet (*L. gigantea*) genome (http://genome.jgi-psf.org/Lotgi1/Lotgi1.home.html, last accessed May 26, 2014), and the sea anemone (*N. vectensis*) genome. All gene models on the scaffolds encoding GPHα2/GPHβ5, SOSD, and Grem in the *B. floridae* v2.0 assembly (http://genome.jgi-psf.org/Brafl1/Brafl1.home.html, last accessed May 26, 2014) were retrieved; BLASTp was used to identify homologs for human, limpet, and sea anemone. A relaxed substitution matrix (BLOSUM45) and short word size (2) were employed to identify divergent homologs, which were considered legitimate if they had an *E* value ≤ 1e^−^^20^ and an identity/similarity of 30%/50%, respectively, along with greater than 50% coverage. *Branchiostoma floridae* protein models were considered homologous if they met these criteria, and there were no more than four closely related human paralogs, with the majority belonging to the same ancestral CLG (Putnam et al. 2008). Homologs identified in limpet and sea anemone that had been previously confirmed against both amphioxus and human were also included. Supplementary figure S5, Supplementary Material online, lists the amphioxus gene models on scaffolds 25, 65, and 96 that had human, limpet, and sea anemone homologs used to construct figure 5.

## Supplementary Material

Supplementary table S1 and figures S1–S5 are available at *Genome Biology and Evolution* online (http://www.gbe.oxfordjournals.org/).

Supplementary Data
