# Supplementary material for: Glycoprotein Hormones and Their Receptors Emerged at the Origin of Metazoans
Source: Genome Biol Evol. 2014 Jun 5;6(6):1466–79. doi: 10.1093/gbe/evu118 (PMC4079206; doi:10.1093/gbe/evu118)
Supplement: Supplementary Data [file supp_6_6_1466__index.html]

Glycoprotein Hormones and Their Receptors Emerged at the Origin of Metazoans — Glycoprotein Hormones and Their Receptors Emerged at the Origin of Metazoans — Supplementary Data 

# Glycoprotein Hormones and Their Receptors Emerged at the Origin of Metazoans

## Supplementary Data

files

**Files in this Data Supplement:**

- Supplementary Data - doc file
- Supplementary Data - xls file
