# Supplementary material for: Glycoprotein Hormones and Their Receptors Emerged at the Origin of Metazoans
Source: Genome Biol Evol. 2014 Jun 5;6(6):1466–79. doi: 10.1093/gbe/evu118 (PMC4079206; doi:10.1093/gbe/evu118)
Supplement: Supplementary Data [file supp_evu118_Supp_Fig_combined.doc]

**Fig. S1.** Alignment of the cystine knot domain of cystine knot growth factor superfamily peptides. Cysteines are highlighted in black. Regions of sequence that are significantly different or introduce gaps compared with the cystine knot hormone-like group (GPH, Burs, BGPH, CKH, Grem, NBL1, NDP and SOSD) are highlighted in color: TGF family in blue, PDGF family in green and NGF family in yellow. Sequences were aligned with MAFFT v7.130 (‘linsi’ defaults). Sequence information provided in supplementary fig. S2D.

10 20 30 40 50 60 70 80 90 100 110 120

....|....|....|....|....|....|....|....|....|....|....|....|....|....|....|....|....|....|....|....|....|....|....|....|

**GPHα2_Human**  CHL-----------------HPF-NVTVRSD---RQ-------GT-----------------------C-QGSHV------AQACVGHCESSAFPSRYSV----------LVASGYRHNI

**GPHα2_Urchin**  CHL-----------------VGY-TKKV-RI----P-------G------------------------C-RETKV-----QMNACRGFCQSYSYPSNLATLQ----------NSDYTQIF

**GPHα2_Oyster**  CYR-----------------MGH-TRTV-QI----P-------G------------------------C-LEFNV-----TTNACRGFCESYAIPSSQRT-----------LSANTRHIL

**GPHα2_RedFBeetle**  CHK-----------------VGH-TRKI-SI----P-------E------------------------C-VEFHM-----TTNACRGFCESWAVPSGPKA--------------TPTQPV

**GPHα2_Cbriggsae**  CKK-----------------VGS-EELI-DE----E-------G------------------------C-DLLII-----RINRCSGTCFSFTFPNPLTK------------------KY

**GPHβ5_Human**  CAV-----------------REF-TFLA-KK----P-------G------------------------C-RGLRI-----TTDACWGRCETWEKPILEPP------------------YI

**GPHβ5_Urchin**  CHV-----------------RQYLQYKA-EK----P-------G------------------------C-RPQNL-----VLDACFGRCDTYEVPELEFP------------------FK

**GPHβ5_Oyster**  CLV-----------------REY-NLFA-QK----PHVTP--TGDVLE--------------------C--SGFV-----KVNSCWGRCDSSEIADYKIP------------------FK

**GPHβ5_RFBeetle**  CHR-----------------RMY-TYRV-TQ--TDD------NGKQ----------------------C--WDTL-----SVMACWGRCDSNEISDWRFP------------------YK

**GPHβ5_Cbriggsae**  --V-----------------PGF-NPLV-QK----D--AAG-KE------------------------C-RGN-V-----ELPFCKGYCKTSESGTHGFP------------------PR

**Bursα_Urchin**  CRK-----------------LGL-QYKL-AR----P-------G------------------------C-RPVTL-----DSVGCRGTCSGYTRISPNNY-----------------LEV

**Bursα_Capitella** CEV-----------------VPV-ILNV-SP----P-------RRYAAI-------------------C-NSTQV-----LTFGCQGNCNSYVEMSKKDP-----------------QRA

**Bursα_RedFBeetle**  CQV-----------------TPV-IHVL-QY----P-------G------------------------C-VPKPI-----PSFACIGRCASYIQVSGSKI-----------------WQM

**Bursβ_Urchin**  CEL-----------------SHG-ETTI-DV----EVVDD---NLQRTL-R-----------------C--RKRV-----QVNQCEGKCISQVSPTVLQH------------------GF

**Bursβ_Capitella** CNT-----------------LHS-TVTF-SS----PKTIELEDGRRRRI-F-----------------C--TGEV-----TVKKCEGFCTSRVSPSVVQY-----------------PGF

**Bursβ_RedFBeetle**  CET-----------------LMS-DINL-IK----EEFDEL--GRLQRI-------------------C--NGEV-----AVNKCEGSCKSQVQPSVITP-----------------TGF

**B/GPH1_SeaAnemone**  CKL-----------------SGY-TMEV-TV----H-------S------------------------C-QPRKI-----SVNTCVGTCVSSALPAAG-------------------LRI

**B/GPH2_SeaAnemone**  CQP-----------------RQG-SVDV-RL----T-------G------------------------C-PEGKA-----FLHLCVGTCYTEDNVV----------------------RD

**B/GPH2_Coral**  CQP-----------------RLN-DVDI-KV----V-------G------------------------C-AKRTV-----RIQQCKGTCRSEES------------------------FN

**B/GPH1_Coral**  CVA-----------------RGF-VMKI-KV----V-------G------------------------C-QERLV-----LINSCLGTCVSYSTPSGSRY------------------EQ

**B/GPH1_Hydra**  CHP-----------------VSFPAVRVDIS----P-------R------------------------C-KDVRV-----AMNQCVGACESMDKNPSN--------------------ER

**B/GPH2_Hydra**  CSA-----------------VPVNAEKI-NV----P-------G------------------------C-YARKL-----RMNKCIGACHSNSGSPDVNG-----------------INS

**B/GPH_Placozoan**  CLPVT-------------HIFRH-REKI-GD----D-------T------------------------C--SGSV-----VVQSCAGYCSSASFPSIDPP------------------FY

**CKHa1_CombJelly**  CER-----------------HEY-KVPI-VD---NE--------------------------------CVRPRNIVF--NDVYGCRGNCESVSMPRKITT-----------TGNVMTIGP

**CKHa1_Sponge1**  CDWLVE------------------SVIF-SH----P-----TDS------T-----------------C-------QGSGYVGSCRGMCLSSAQPRLYDSRD---------AVLQAYNPF

**CKHa_Sponge2**  CSWVT-------------HKMTF-YKEI-------Q-------GHN----------------------C-SSEPI-----DVFKCTGMCASNINPRVIDRKPPP-------DQTSYKHLF

**CKHb1_Sponge1**  CVR-----------------FVA-NITL-SR----E-------G------------------------C-SPSTI-----EVPTCSGACNSFVHYVTTSP------------------HK

**CKHb_Sponge2**  CHR-----------------KRK-NFTI-SY----P-------GNEPGL-V-----------------C-KDGRE-----PFPYCEGGCRSLTRVIQSPP------------------FV

**CKHb1_Sponge3**  CKP-----------------SHY-RHTI-EV---SP-------T------------------------C--RRTV-----SIKQCSGTCTSFTMLSRDGL------------------NI

**GREM1_Human**  CKT-----------------QPL-KQTI-HE----E-------G------------------------C-NSRTI-----INRFCYGQCNSFYIPRHIRK----------------EEGS

**GREM_Urchin**  CKT-----------------QPL-KQRI-EE----P-------G------------------------C-IPRTI-----ANRFCYGQCNSFFIPKQAAS----------------YNEA

**GREM_RedFBeetle**  CKT-----------------EPL-IQKV-KE----E-------G------------------------C-LTRTV-----INRFCYGQCNSFYIPKNPKKRHRHIPVSEETEDEDQNGPA

**GREM_Cbriggsae**  CDG-----------------QKF-KQRI-RV----D-------G------------------------C-LTKVV-----VNRLCHGTCASIFIPRHSTKKL---------------KAA

**GREM_SeaAnemone** CKL-----------------RPV-LQKL-HH----P-------G------------------------C-NSSFI-----MNNMCYGQCMSFFIPRHFTS--------------------

**GREM_Coral**  CKT-----------------RAF-KQTI-KI----P-------G------------------------C-LPVQV-----VNNFCYGQCNSMYIPNHASE-----------------KPL

**GREM_Hydra**  CMV---------------HGTKY-QQVI-SE----R-------D------------------------C-KAVTI-----DVPFCYGQCNSFFIPRYGED--------------------

**NBL1_Human**  CEA-----------------KNI-TQIV-GH----S-------G------------------------C-EAKSI-----QNRACLGQCFSYSVPNTFPQ----------------STES

**NBL1_RedFBeetle**  CQT-----------------TPI-QQVV-AS----P-------GY-------------------------ESVTI-----HNNVCVGACYSYSIPSTQPA--------------EPGELL

**NBL1_Coral**  CRS-----------------VEI-QHSI-QE----P-------G------------------------C-ETKVI-----ENRACMGQCFSYYAPGTHPR-------------KDLSDKR

**NBL1_Placozoan**  CKL-----------------AKI-EQVL-SH----P-------G------------------------C-ISKTI-----SNHICVGQCYSYRIPKSYPP--------------EAGQEN

**NDP_Human**  CMR-----------------HHY-VDSI-SH----P-----LYK------------------------C-SSKMV-----LLARCEGHCSQASRSEPLVS-----------FSTVLKQPF

**NDP_Urchin**  CVR-----------------YYH-TERI-SH----P-----RKP------------------------C-QSKII-----LMSRCAGQCEMASSADPVVS-----------FKSHLRHPF

**NDP_Oyster**  CMK-----------------HYF-VHTI-EH----P-----TKN------------------------C-EKKHI-----LLARCKGFCSKSKTEPRVT------------FSPVLYRPF

**NDP_Coral**  CKR-----------------KPV-SMNV----------------------------------------C-GGREI-----RAYKCEGACESESKILMGDP------------------WF

**SOSD1_Human**  CRE-----------------LRS-TKYI-SD------------G----------Q-------------CTSISPL-----KELVCAGECLPLPVLPNWIG---------GGYGTKYWSRR

**SOSD_Urchin**  CVE-----------------LRS-KRYI-SD------------G----------F-------------CTSTRPI-----TEVVCAGSCIPEDYTGWYFE------------HIKVWGNK

**SOSD_Capitella**  CKE-----------------LRS-KRYI-SD------------G----------F-------------CTSVKPV-----TEVVCTGHCLPIRNLPWYAE------------FIKVWART

**SOSD_SeaAnemone**  CVG-----------------IRI-KRYV-SN------------G----------F-------------CTSNRAI-----KDMICEGRCLPMDELPFFPD------------YSKVLSAT

**SOSD_Coral**  CTG-----------------IRF-RRYV-SN------------G----------F-------------CTSRRPL-----RDMICDGDCLPMDQLPFFPN------------FSKIISRH

**SOSD_Placozoan**  CDG-----------------KKI-HRYV-MK------------G----------L-------------CISKRSI-----KDLLCEGACRPNNIPQDVSA-------------------S

**TGFβ1_Human**  CCV-----------------RQL-YIDFRKD-----------LGWK--------W-------------IHEPKGY-----HANFCLGPCPYIWSLDTQYS--------KVLALYNQHNPG

**TGFβ_Urchin**  CCL-----------------RPL-VINFRRD-----------LKWN--------W-------------IRQPRNY-----SPNFCAGSCPYILSADTSHA--------SVLSLYKHLNPD

**BMP_Oyster**  CAK-----------------HEM-YVDF-DE-----------IGWSGW--------------------IISPKGY-----NAYHCKGACPFPLGQSQKPTNHATVQS----IVHALKVGN

**DPP_Beetle**  CRR-----------------RQM-YVDF-GS-----------VGWNDW--------------------IVAPLGY-----DAYYCGGECEYPIPDHMNTTNHAIVQS----LVNSMK-PK

**DBL1_Cbriggsae**  CRR-----------------TDL-YVDF-DD-----------LGWQDW--------------------IMAPKGY-----DAYQCQGSCPNPMPAQLNATNHAIIQS----LLHSLK-PD

**TGFβ_SeaAnemone**  CQR-----------------HPL-YVDF-TD-----------VGWNDW--------------------IVAPPGY-----HAFYCTGVCPYPIAKHLNATNHAIVQT----IMNTV--DS

**TGFβ_CombJelly**  CRR-----------------QDL-WINF-DE-----------IGWD--------F-------------VITPKDV-----NIGDCGGQCLDADSNIPHAV----VKQ----LLQKMHPAR

**TGFβ_Sponge1**  CRL-----------------QFH-HVNL-SD---------------------------------YFHDIVEPSQY-----SISYCAGTCDSAPDVGTRTW-------V--LFSEHQRDPA

**PDGFA_Human**  CKT-----------------RTV-IYEI-PRSQVDP------TSAN----------------FLIWPPC-----V-----EVKRCTGCCNTS----------------------------

**PDGF_Urchin**  CEP-----------------RST-LLAT-SE-VIDE-------GLTEN----E------VEYAIYWPPC-----I-----TVNRCGGCCSTE----------------------------

**PDGF_Oyster**  CSP-----------------RYQ-CVVI-PR-YTDP-------------------------NVIYYPPC----------TRVLRCGGCAPSE----------------------------

**PDGF_Cbriggsae**  CNL-----------------QNV-CVPV-PQLSDDP-------------------------QLLIYPKC----------YEVKQCVGSCCNS----------------------------

**PDGF_RedFBeetle**  CMP-----------------ELQ-TVNI-AA-SDDP-------------------------SVLYIPQC----------TRVERCGGCCSHH----------------------------

**PDGF_SeaAnemone**  CRP-----------------RPT-VISI-DD----P-------GHN-----------------------YKPYQV-----VLHRCSGTCGDSPPN-------------------------

**NGFB_Human**  CDSVSVWV----------GDKTT-ATDIKGK----EVMV---LGEVNIN-------NSVFKQYFFETKCRDPNPV-D-----SGCRGIDSKHW---------------------------

**NGF_Urchin**  CESTSGWIVKKWGTDMYGQ-----NVTILSE--------IMTAG------------NIQVTQWFYETACARPQGL----HGVQRCLGIDNNNY---------------------------

**NGF_Oyster**  CPVVTHK----------------------TD----PLG-----GLSRDGRLLRLYRDPRTIQRFYETSC--AAGV-----LNRPCR------------------------------YVDS

**NGF_RedFBeetle**  CPS---------------------VLEM-IE----PQG-----GKNRQDQFVELYRDGDYTQRFYERSC--HKDI-----LGKPCR-------FMDK---------------------KL

130 140 150 160 170 180 190 200 210 220

....|....|....|....|....|....|....|....|....|....|....|....|....|....|....|....|....|....|....|....|..

**GPHα2_Human**  TSVSQCCTISGL---KKVKV-QL-QCVGS--------------RREELEIFTARACQC----------DMCRLSRY--------------------------

**GPHα2_Urchin**  TTHGSCCSIATT---HDVNI-RL-QCLDN--------------YEYVDTFKSAASCEC----------SLCV----IG------------------------

**GPHα2_Oyster**  TSRAECCGIEET---HDITV-SV-GCADG---------------LREVTFKSAKTCACSVRR----------------------------------------

**GPHα2_RedFBeetle**  TSVGQCCNIMET---EPVEA-RV-LCVDG---------------VRTLTFKSAVSCSC----------YHCKKD----------------------------

**GPHα2_Cbriggsae**  SVHAKCCRMVEW---EMLET-EL-KCSEG---------------TRKLRIPSATQCEC----------FDCLLQ----------------------------

**GPHβ5_Human**  EAHHRVCTYNET---KQVTV-KLPNCAPG--------------VDPFYTYPVAIRCDC----------GACSTATTECETI---------------------

**GPHβ5_Urchin**  SSHHEMCSYHQV---QLATI-ELDDCDPG--------------VNRTYTYRNAKSCKC----------RNCTPFNTFCFGLSL-------------------

**GPHβ5_Oyster**  ISNHPVCTYSRV---QKRRV-RLPNCHP-------------EHPDPYYVVYDALACSC----------RYCNSKYTSCETLNG-------------------

**GPHβ5_RedFBeetle**  KSNHPVCVHYGR---NRSVV-TLRHCEEG-----------ANPSAARYEYLEAAGCKC----------QQCSSSDTSCEGLRYRPQRS--------------

**GPHβ5_Cbriggsae**  VQISKVCTLVQT---SIRKV-ILDDCDEG-----------AAESIKFVNVPHGSECEC----------SAVPLEQNHS------------------------

**Bursα_Urchin**  ERSCTCCQEMGF---LERTQ-RL-QCPTL------------NPPFRDVTYRIPRRCSC----------RPCRSVASVSRVQTLEDLRL--------------

**Bursα_Capitella** LRSCRCCEPIKF---GVHLA-TM-RCQGN--------------RMLRAPLKFAIECNC----------RPCFSLKTDLSVLRKMI-----------------

**Bursα_RedFBeetle**  ERSCMCCQESGE---REASV-SL-FCPKAKP---------GERKFIKVTTKAPLECMC----------RPCTGVEESAVIPQEIA-----------------

**Bursβ_Urchin**  DKKCHCCREHGM---VHKKV-VMTNCYDHALG------ITDPDFTHQVTLKQPEACRC----------QICTF-----------------------------

**Bursβ_Capitella** KKNCTCCKETRL---RNRVV-LLTNCFEGDQA--------LPDESASLFIREPDDCAC----------SSCEI-----------------------------

**Bursβ_RedFBeetle**  LKECYCCRESFL---RERTI-TLTHCYDPDGVR----LTAETVNSMDVKLREPAECKC----------YKCGDFSR--------------------------

**B/GPH1_SeaAnemone**  EPACTCCQEIES---HEVEV-GL-WCQASPN---------SAWTQEYHVIKTATKCAC----------RPC-------------------------------

**B/GPH2_SeaAnemone**  EASCTCCKPTKF---RSVQV-DV-ECRHN-----------KAWGIVKHVMREHEHCAC----------APCLG-----------------------------

**B/GPH2_Coral**  SRACRCCTPVKR---TEIPV-QL-LCKNS------------VLYIHTHIVQAHEQCTC----------SRCLLH----------------------------

**B/GPH1_Coral**  VKSCNCCQQIKT---KKVDV-GL-WCRDRRFP------SKIVLYRYYHTVESVTQCAC----------VSC-------------------------------

**B/GPH1_Hydra**  KSVCRCCQPVDF---IERNIVKEVACMSNDK---------AVTTLMTIKIFEPIRCEC----------RNCKPS----------------------------

**B/GPH2_Hydra**  AGFCACCQPILF---QEFEI-DL-NCKEN-----------DKNVVKKLKIQNPIECNC----------RQCSRDE---------------------------

**B/GPH_Placozoan**  RPVVRCCQSINT---TTVPL-QL-KCGSNG-------------FKKIIRATYHVQCAC--------------------------------------------

**CKHa1_CombJelly**  VTHCKCCSAIEV---ERRTY-TA-KCRRKG----------RSTILKTYEILSAVTCAC----------TSCGPDNYEFARNRARDTKR---------KRNRS

**CKHa1_Sponge1**  ETDCKCCQPKIER-CDNISV-DM-TCANN-----------TVLRGVKIFSCSAFECGC----------ATCKP-----------------------------

**CKHa_Sponge2**  SQNCQCCQPDQDNYIQNKSI-LY-YCVLPNGE--------KYSTPAMTMIANPTKCAC----------QPC-------------------------------

**CKHb1_Sponge1**  QSQCSCCQPTNYR-IAKRTA-RF-QCAGG------------TIETVTFFVSVAQDCNC----------SSCGQVPTLSHIV---------------------

**CKHb_Sponge2**  ESVCNCCSFLDAE-VRKRTV-DF-KCTDSKGN--------EVTKKVLMYFPRITDCTC----------VRCGQQPNVN------------------------

**CKHb1_Sponge3**  APHCVCCQPVSSS-IKYKNV-VL-SCPEM------------SDQLIVRKVATVNECIC----------RRCS------------------------------

**GREM1_Human**  FQSCSFCKPKKF---TTMMV-TL-NCPEL------------QPPTKKKRVTRVKQCRC----------ISIDLD----------------------------

**GREM_Urchin**  FKSCSFCKPFRV---NHITV-TL-RCPGQ------------NPPIKRKRVPRVKRCRC----------MAVDVP----------------------------

**GREM_RedFBeetle**  FKACAFCRPSKF---TWISV-TL-KCPSL------------MPPFRKKRIQRIKQCKC----------IAANVN----------------------------

**GREM_Cbriggsae**  FRSCAACAPSEY---DYVDI-TL-DCPGK------------SPPTTTKTIVKVKSCKC----------REVRIASF--------------------------

**GREM_Anemone**  ---CAFCTPVSK---NVVSV-HL-KCAGD--------------LKVVKKVSIIQSCSC----------RPCGNQYI--------------------------

**GREM_Coral**  FESCTTCMPKRS---FTKTV-TL-RCPTL------------PVKFRKHKYLHSKKCRC----------STVRRTTSLGE-----------------------

**GREM_Hydra**  FNSCNTCMPKKW---NETKV-QL-NCKTD-----------GEWRVVIKKVLLIGSCEC--------QEVHCDKKKR--------------------------

**NBL1_Human**  LVHCDSCMPAQS---MWEIV-TL-ECPGH-----------EEVPRVDKLVEKILHCSC----------QACGKEPSHEGLSVYVQGED--------------

**NBL1_RedFBeetle**  GPYCDSCQPVET---KCYHV-TL-HADGKN---------TEGPKTFQKRVQIILKCSC----------MSCEKYHREDCEITDQTTLEL-------------

**NBL1_Coral**  MKYCDMCKPSLK---SWTKV-SL-DCPGT------------NHGQVDKLVEVIYSCTC----------QKCIKDLKQG------------------------

**NBL1_Placozoan**  LQHCECCHVVDH---TWNTV-EL-KCPT-------------LKNNVDKLVQYIRSCDC----------RRCHHTKS--------------------------

**NDP_Human**  RSSCHCCRPQTS---KLKAL-RL-RCSGG--------------MRLTATYRYILSCHC----------EECNS-----------------------------

**NDP_Urchin**  KYRCQSCQDHIS---IMKAV-LL-RCQGN--------------ERVYATYRYILSCEC----------ASCKR-----------------------------

**NDP_Oyster**  NYHCKCCRDSLS---IMKAV-SL-NCEGD--------------KPVFATYRYILKCKC----------RNCNFRRW--------------------------

**NDP_Coral**  RAECRCCKSIRT---ETKSV----PCPGGD----------------EEKIRFIHACGC----------GNCNGA----------------------------

**SOSD1_Human**  SSQEWRCVNDKT---RTQRI-QL-QCQDG--------------STRTYKITVVTACKC----------KRYTRQHNESSHNFESMSPA--------------

**SOSD_Urchin**  KRKEWRCVNDSV---RQKKV-TL-LCDNN--------------QTRTYRIKTVRSCKC----------KRLLQQHNESERPNENVERR--------------

**SOSD_Capitella**  KILEWRCVDDVI---RRKRV-RL-LCENG--------------ESRSYKIKVVRSCKC----------KRFMRQQNESPERQRTKTRK--------------

**SOSD_SeaAnemone**  KR-EWRCVPDRK---RRRNV-NV-TCNDG--------------TKRKYRILVVRSCKC----------KRYTRKQNMTNAGGQPENKK--------------

**SOSD_Coral**  KR-EWRCVADDR---RTKKV-KL-ICNDG--------------TMRKYRVSVVRSCKC----------KRYTRKQNQTRPNSEK------------------

**SOSD_Placozoan**  KRYIWRCVAHFR---KTIRV-KM-NCFDG--------------TTKYAFPSVVRKCVC----------KRYRRRQNETKQKILKRSLE--------------

**TGFβ1_Human**  ASAAPCCVPQAL---EPLPI-VY--------------YVGRKPKVEQLSNMIVRSCKC--------------------------------------------

**TGFβ_Urchin**  ASPSPCCSPQTF---KPLTI-LF--------------YNRGRPEIRQLNNMIIETCRC--------------------------------------------

**BMP_Oyster**  DVSTPCCVPNKL---YSISL-LYFDDDE-------------NVILKQYDDMVAASCGC----------H---------------------------------

**DPP_Beetle**  EVPGPCCVPTQL---GQMSM-LYLGSDG-------------SVILKNYKEMVVVGCGC----------R---------------------------------

**DBL1_Cbriggsae**  EVPPPCCVPTET---SPLSI-LYMDVDK-------------VIVIREYADMRVDSCGC----------R---------------------------------

**TGFβ_SeaAnemone**  NVPNACCIPTTL---NPISI-LSLNEFD-------------KVVLKNYKDMVIEGCGC----------R---------------------------------

**TGFβ_CombJelly**  NAGILCCQGANY---KEISA-LY-------------FKSKHEIVIGDMPKIIVKECRC--------------------------------------------

**TGFβ_Sponge1**  TVPPLCCVPERY---TSLTV-LV--------------QRDDTTFLETLLNFAAKSCHC--------------------------------------------

**PDGFA_Human**  ---SVKCQPSRV---HHRSV-KVAKVEYVRKK--------PKLKEVQVRLEEHLECAC----------ATTSLNPDYREEDTG-------------------

**PDGF_Urchin**  ---ILQCAPNNT---RTREM-QVLRLTADQQST-----QIQLSGLISISVREDESCSCECRI----KPQDCHPTREIH--------RNCQCECAA---EYEC

**PDGF_Oyster**  ---VLTCAPKQT---SVKHV-VVFKSTIPYPGS----PSTDYEGLQLVNITQHDSCEPTCTV----KPYHCNPLQSFIA-------RECRCMCTN-RGSVRC

**PDGF_Cbriggsae**  ---LETCHPGTIN-LVKKHVAELLYIGNGRF---------MFNMTREITMEEHTSCSC----------FDCGSNTPVCAPGFVVG-RSCKCECANKEERNNC

**PDGF_RedFBeetle**  ---LLACQPETK---EEIPF-KVIKTQYTGGK------KLKVLSKEVILVEKHTKCKCDCKV----RAEDCNRFQEYRK-------SECRCACTNYDEEKKC

**PDGF_SeaAnemone**  ---IKTCFPKTL---EDVPL-QATDIGQG--------------VSQTVYVKNHTSCMCDCA-------EGCTSENAIRD-------KNCRCICLD---PNNC

**NGFB_Human**  ---NSYCTTTHTF-VKALTM--------------------DGKQAAWRFIRIDTACVCVLSR------KAVRRA----------------------------

**NGF_Urchin**  ---DSVCLTKSAW--VYAMI----RTARGEEG-----WTW---------IAISSSCNCAVRQLSLL--EQIGRRSRLTR-----------------------

**NGF_Oyster**  MWRSKCVQSYTY---VYAIVQDF----------------NTTQPYRMDYMRIKSGCICKVDD----------------------------------------

**NGF_RedFBeetle**  HEHSKCIQKYSY---TYALVRDLGRHHMQRPKNFPSLAAPGGATWTLDYIRIRSGCACVVTP---NKKKRDRLKHRKTRVKDDT------------------

**Fig. S2A.** Maximum-likelihood tree (WAG+I+G) of cystine knot hormone-like sequences.


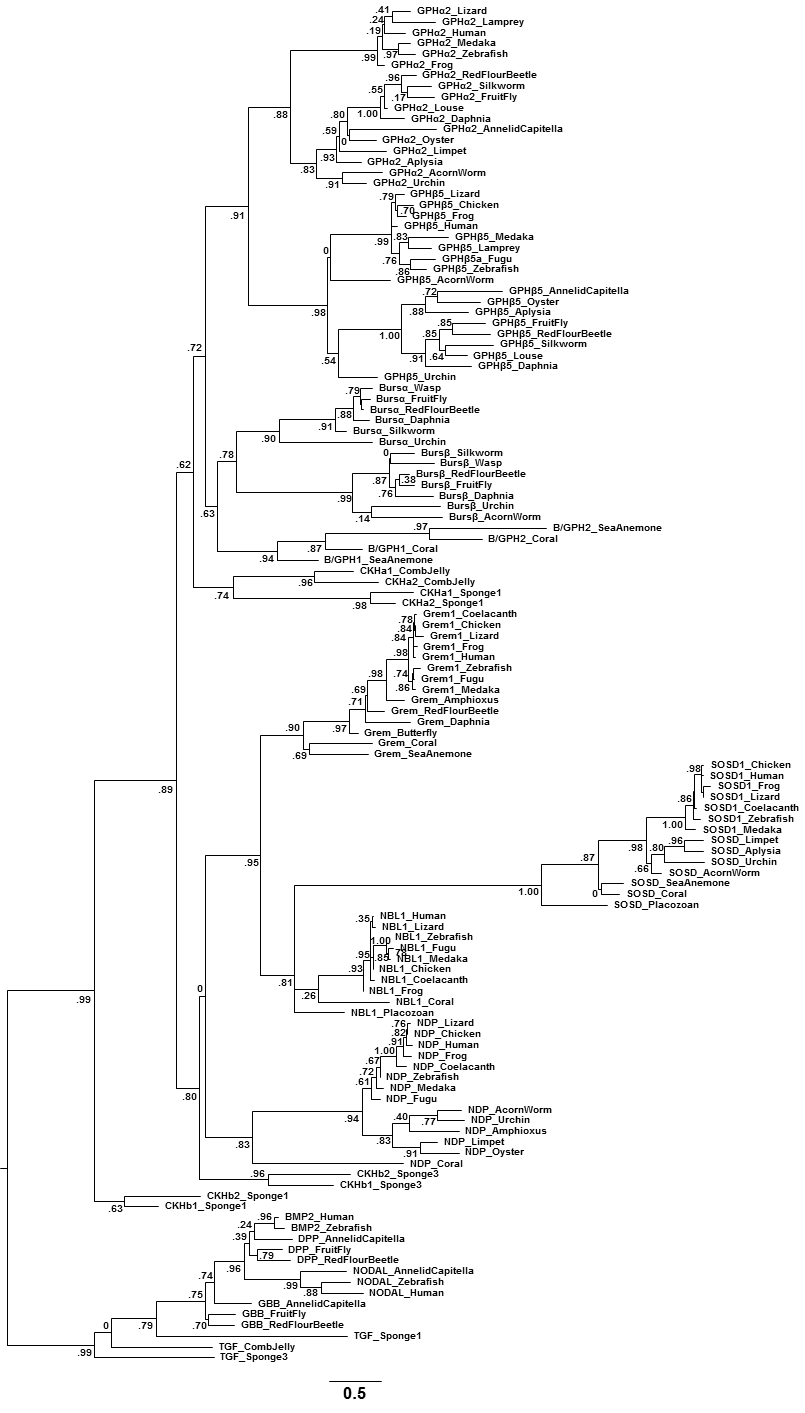


**Fig. S2B.** Bayesian inference tree (WAG) of cystine knot hormone-like sequences.

**
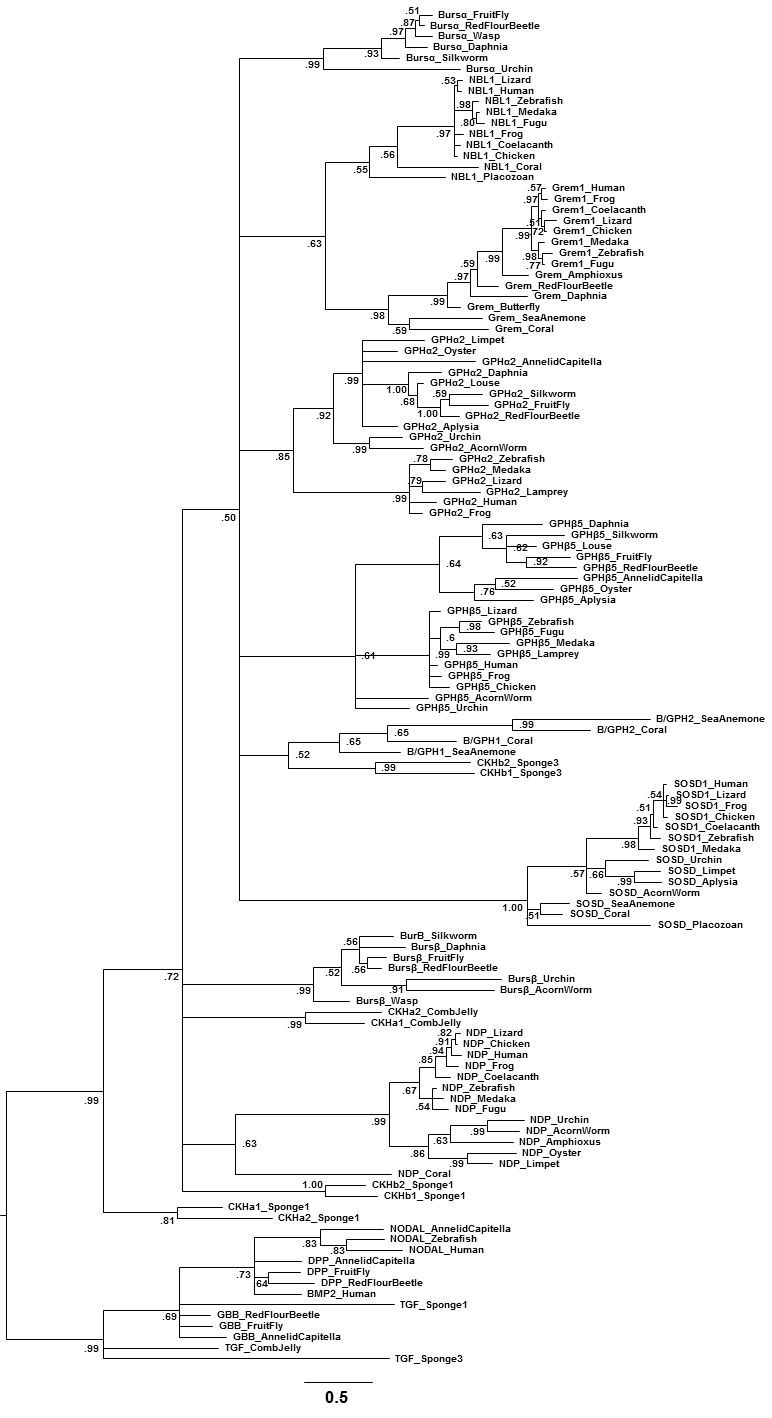
**

**Fig. S2C.** Alignment of cystine knot hormone-like sequences used to produce phylogenetic trees in figs. 2, S2A and S2B.

10 20 30 40 50 60 70 80

....|....|....|....|....|....|....|....|....|....|....|....|....|....|....|....|....|....

**GPHα2_Human**  IPGCHLHPFNVTVRSGTCQG-SHVAQACVGHCESSAFPSRYSVLNITSVSQCCTISGLKKVKVQLQCVGSRREELEIFTARACQCDMCR

**GPHα2_Lizard**  GLGCHLHSFNVTLKSGTCRG-IQTLQACVGFCESSAFPSKYSVLNVTSVSQCCTIAKMQKVKVRLRCGESWRETMELFTAKACQCDMCR

**GPHα2_Frog**  RPGCHLHPFNVTISSGTCRG-TQVINACVGYCESSAFPSKYSVLNITSASQCCTISKMQKVKVQLYCGGSRHEEIEIGTALSCQCDMCR

**GPHα2_Medaka**  APGCHLFPFNVTIRSSTCKG-SHVLHACVGYCESSAFPSRYSVLNITSASRCCTISKDAKVKVRLDCPRGRHDEIEILTAKACRCDMCR

**GPHα2_Zebrafish**  TPGCHLYPFNVTVRTGTCRG-TQLVYACVGYCESSAFPSRYSVLNITSSSRCCTISKDAKVKIRLHCPRGRHADMEILSARACRCSMCH

**GPHα2_Lamprey**  KPGCHPYPFELALRRGACL---ARVHACVGFCESSAFPSKYSVLNITSVAQCCTIRRLEKVRTRVKCGTSNH-VMEVFTAKECQCDVCR

**GPHα2_AcornWorm**  RPGCHLVGYVKTVRVPGCHEEEVRMNACRGYCTSYSYLSSPATLIFTAKGSCCTIDETHNVAVTLQCQNGKVYRDVFKSAKSCVCGICD

**GPHα2_Urchin**  SPGCHLVGYTKKVRIPGCRETKVQMNACRGFCQSYSYPSNLATLIFTTHGSCCSIATTHDVNIRLQCLDNYEYVDTFKSAASCECSLCV

**GPHα2_Limpet**  RPGCTKVFHARVVKIPYCVEFRVETNACRGFCESYAHPSLSSTLRITSRSECCSITSTHDVTVRVWCIGTGT-NKTFKSAVDCGCSICR

**GPHα2_Aplysia**  APGCHLVGHTRTVSIPGCVSFEVTTNACRGFCVSYAIPSPSHTLVITSRAECCGIVDTHDVKVWVACRSGFQ-QKTFKSARSCQCSICR

**GPHα2_Oyster**  KAGCYRMGHTRTVQIPGCLEFNVTTNACRGFCESYAIPSSQRTLILTSRAECCGIEETHDITVSVGCADGLR-EVTFKSAKTCACSVRR

**GPHα2_Capitella**  ITDCAPQGHKRLISVPGCLEFNATTNGCRGYCPSCAYPTPIWLSPITSEAQCCSIKDTHSVVYRVRCFDKIRKTFVFKSASTCECSSCS

**GPHα2_FruitFly**  RPGCHKVGNTRKITIPDCVEFTITTNACRGFCESFSVPSIPLFKPVVSVGQCCNMMKSEEIQRRVLCIEGIR-NVTFNSALSCSCYHCK

**GPHα2_Silkworm**  KPGCHRIGHTRNISIPDCVEFKITTNACRGYCESWSLPSIMLGFPVTSLGQCCNIMEAEDVPVKVLCLDGER-NLIFKSAVSCACYHCQ

**GPHα2_RedFBeetle** KPGCHKVGHTRKISIPECVEFHMTTNACRGFCESWAVPSGPKATPVTSVGQCCNIMETEPVEARVLCVDGVR-TLTFKSAVSCSCYHCK

**GPHα2_Louse**  RPGCHKVGHTRKVSIPDCVEFHITTNACRGFCESWSIPSGMETLVITSVGQCCNIMETEDVDVKVMCLEGIR-ELTFKSAKTCSCYHCK

**GPHα2_Daphnia**  LSGCHQVGHTRRVTIPDCVSFMITTNACRGFCESWSVPSSWEALVITSVGQCCNIMASEDVTVRVMCLGGPR-DFTFKSAKTCSCFTCK

**GPHβ5_Human**  FVGCAVREFTFLAKKPGCRGLRITTDACWGRCETWEKPILEPPY-IEAHHRVCTYNETKQVTVKLNCAPGV-PFYTYPVAIRCDCGACS

**GPHβ5_Chicken**  FIGCAVREFTFLAKKPGCKALRITTDACWGRCETWERPLLHPPY-IESYHRVCTYNETKLVTVMLKCGPGV-PFYTYPVAIRCNCDFCS

**GPHβ5_Lizard**  FIGCAVREFTFLAKKPGCKGMRITTDACWGRCETWEKPVLDPPY-VEAHHRVCTYNETKLVTVKLNCAANV-PFYTYPKAIRCDCSMCL

**GPHβ5_Frog**  FIGCAVREFTFLAKKPGCKGLRVTTDACWGRCETWEKPVLDPPY-IEAHHRVCTYNETKLVTVKLNCSPDI-PFFTYPVAVRCDCDICS

**GPHβ5_Medaka**  FRGCAVREFTFVAQKPGCKELRITTEACWGRCHTWERPILEPPF-IHRHHRVCTYSHIRLLSARLGCLPHV-PLYHYPAALQCHCSGCS

**GPHβ5_Fugu**  FVGCAVREFTFLARKPGCGALQITTDACWGRCETWEKPVLDPPF-VDTYQRVCTYNRSRVVTVSLGCLPDV-PAYSYPVALRCDCSVCV

**GPHβ5_Zebrafish**  FIGCAVREFTFLARKPGCGGLHITTDACWGRCETWQKPVLEPPF-IESHQRVCTYNETRQETVLLNCTAGV-PSYSFPVALRCDCGLCL

**GPHβ5_Lamprey**  FVGCAVREFTFVARKPGCRGARVTTDACWGRCQTWEKPTLEPPH-VEAHHRVCSYNETRAGSVRLGCAPHV-PVFSFPVALSCVCKMCS

**GPHβ5_AcornWorm**  TTRCLVREYNKYIVKSGCEPQRITIDACWGRCQTFEVPDLLPPY-TAANHTMCQYDVTEWRTVELDCNPGV-RTFVYLNALSCSCTKCS

**GPHβ5_Urchin**  RLGCHVRQYLYKAEKPGCRPQNLVLDACFGRCDTYEVPELEFPF-KSSHHEMCSYHQVQLATIELDCDPGV-RTYTYRNAKSCKCRNCT

**GPHβ5_Aplysia**  TLSCHVRSYQFRVTKPRCS-GIVTVNSCWGRCDSSEIGDYLMPY-RISHHPVCTYTGRVPRQVTLGCEDY-PPTFEVFDAAGCECRLCD

**GPHβ5_Oyster**  VTACLVREYNL-FAQKECS-GFVKVNSCWGRCDSSEIADYKIPF-KISNHPVCTYSRVQKRRVRLNCHPEHPPYYVVYDALACSCRYCN

**GPHβ5_Capitella**  ERFCHTRPYTYLASQPHCN-GSITVNACLGSCESQEIPDYRMPY-KLSRHPVCTFGEVRVRGFLLNCHADHPPFHITHEALSCRCKQCD

**GPHβ5_FruitFly**  PLGCHRRVYTYKVTQSECW-DYVSVWSCWGRCDSSEISDWKFPY-KRSFHPVCVHAQRQLVVAILNCHPKAESKYQYMEAVNCHCQTCS

**GPHβ5_RedFBeetle** TIECHRRMYTYRVTQTQCW-DTLSVMACWGRCDSNEISDWRFPY-KKSNHPVCVHYGRNRSVVTLHCEEGANARYEYLEAAGCKCQQCS

**GPHβ5_Silkworm**  SVRCKLKRHSHKVMQTRCW-DDVKIVSCWGYCLSYEISDWQFPY-KESHHPVCVHGERRHASVKLNCDPGVEEIYHYVEAVNCRCQVCS

**GPHβ5_Louse**  SLDCNRRAYSHRVTQTICW-DTITVVSCWGRCDSNEISDWRFPY-KRSHHPVCIHGERKPKTVILNCDNDVEEIHEFMEAISCECSLCK

**GPHβ5_Daphnia**  TPTCFRRPYTFKVYQESCW-DVVTVTSCWGRCSSNEIADWRFPF-KRSQHPVCQHDSILPRAIILNCDPEVNELYMALDAVTCRCELCH

**Bursα_Urchin**  LADCRKLGLQYKLARPGCRPVTLDSVGCRGTCSGYTRISPNNY-EVERSCTCCQEMGFLERTQRLQCPTLNPRDVTYRIPRRCSCRPCR

**Bursα_FruitFly**  GDDCQVTPVIHVLQYPGCVPKPIPSFACVGRCASYIQVSGSKI-QMERSCMCCQESGEREAAVSLFCPKVKPKKVLTKAPLECMCRPCT

**Bursα_Wasp**  IDECEVTPVIHVLQYTGCVPKPIPSFACKGRCSSYLQVSGSKI-QMERSCMCCQESGEREASVSLFCPKTKARKVITKAPLDCMCRPCT

**Bursα_Silkworm**  CQECQMTAVIHVLKHRGCKPKAIPSFACIGKCTSYVQVSGSKI-QMERTCNCCQESGEREATVVLFCPDAQNRKVSTKAPLQCMCRPCG

**Bursα_RedFBeetle** TDECQVTPVIHVLQYPGCVPKPIPSFACIGRCASYIQVSGSKI-QMERSCMCCQESGEREASVSLFCPKAKPIKVTTKAPLECMCRPCT

**Bursα_Daphnia**  ADECQLTPVIHVLQYPGCIPKPIPSFACTGKCTSYVQVSGSKL-QTERSCMCCQESGEREATVSLLCPKAAPRRVVTRAPVDCMCRPCT

**Bursβ_AcornWorm**  EDNCFPGTTDLVIGTKYCS-GMAVLKKCEGSCISQVTPSVR--YGFSKECKCCRERTRTLRTVVLACFDVDNHQMVLQEPSDCACQVCA

**Bursβ_Urchin**  GETCELSHGETTIQTLRCR-KRVQVNQCEGKCISQVSPTVLQ-HGFDKKCHCCREHGMVHKKVVMNCYDHLGHQVTLKQPEACRCQICT

**Bursβ_FruitFly**  DENCETLKSEIHLRQRTCN-ADVIVNKCEGLCNSQVQPSVITPTGFLKECYCCRESFLKEKVITLHCYDPDGMDIRLREPTECKCFKCG

**Bursβ_Wasp**  NENCEKLDSEIRIKFRTCS-DEIQVSKCEGYCDSQVQPSIVTSTGFTKECSCCREEFLEERTVYLNCYDSNGMAIKIREPTNCKCIKCG

**Bursβ_Silkworm**  EENCETVASEVHVRLRSCS-GEVSVNKCEGMCNSQVHPSISSPTGFQKECFCCREKFLRERLVTLHCYDPDGMEVRLREPDECECYKCG

**Bursβ_RedFBeetle** EETCETLMSDINLRQRICN-GEVAVNKCEGSCKSQVQPSVITPTGFLKECYCCRESFLRERTITLHCYDPDGMDVKLREPAECKCYKCG

**Bursβ_Daphnia**  SGTCETLPSTIHIISRTCE-GDIGVAKCEGSCSSQVQPSVVHPSGFLKECMCCRESFLRERVVTLHCYDANGLDVKMREPADCKCFRCG

**B/GPH1_SeaAnemone** NQQCKLSGYTMEVTVHSCQPRKISVNTCVGTCVSSALPAAG---RIEPACTCCQEIESHEVEVGLWCQASPNEYHVIKTATKCACRPC-

**B/GPH2_SeaAnemone** KEVCQPRQGSVDVRLTGCPEGKAFLHLCVGTCYTEDNVV-----RDEASCTCCKPTKFRSVQVDVECRHNKAVKHVMREHEHCACAPCL

**B/GPH1_Coral**  SRHCVARGFVMKIKVVGCQERLVLINSCLGTCVSYSTPSGSR--EQVKSCNCCQQIKTKKVDVGLWCRDRRFYYHTVESVTQCACVSC-

**B/GPH2_Coral**  AGDCQPRLNDVDIKVVGCAKRTVRIQQCKGTCRSEESF-------NSRACRCCTPVKRTEIPVQLLCKNSVLHTHIVQAHEQCTCSRCL

**CKHa1_CombJelly**  YGDCERHEYKVPIVDECVRPRNNDVYGCRGNCESVSMPRKITT-GPVTHCKCCSAIEVERRTYTAKCRRKGTKTYEILSAVTCACTSCG

**CKHa2_CombJelly**  ITDCRRSTFVVPIVHEGQKLRQKNIYGCRGHCDSGSVPEQVNNEMQVNYCKCCSAMEYELKTFSIPTRQGGLKTYKIWSAVTCGCQMCG

**CKHa1_Sponge1**  IGDCDWLVESVIFSHSTCQG-SGYVGSCRGMCLSSAQPRLYDSDPFETDCKCCQPKIEDNISVDMTCANNTVRGVKICSAFECGCATCK

**CKHa2_Sponge1**  SDGCNWVVESVTYPHSVCKG-QGYVGSCKGACPSSAVPRMYENEPFESNCKCCEAAVEDKIPIDMKCPNGTIR-VKVCTALSCACSACK

**CKHb1_Sponge1**  AAPCVRFVANITLSREGCSPSTIEVPTCSGACNSFVHYVTTSP-HKQSQCSCCQPTNYAKRTARFQCAGGTIVTFFVSVAQDCNCSSCG

**CKHb2_Sponge2**  QSTCQKIQIDIQIPRAGCSQGVMGVPICSGACNSYMSYKLSFP-YKQEQCSCCTATTYSKRTVSFLCGNTNVVSYNIAAVMECNCATCP

**CKHb1_Sponge3**  DTSCKPSHYRHTIEVSPTCRRTVSIKQCSGTCTSFTMLSRDGL-NIAPHCVCCQPVSSKYKNVVLSCPEMSDIVRKVATVNECICRRC-

**CKHb2_Sponge3**  RHDCSPKDHEHKIQVTRKCSKKVTVKKCMGQCDSSAVPSSDGT-TVEKSCNCCQPEDTELKDFTVRCGGVR-FKREVVTVTKCFCRRVE

**NDP_Human**  PRRCMRHHYVDSISHYKCSSKMVLLARCEGHCSQASRSEPLVSFPFRSSCHCCRPQTSKLKALRLRCSGGMRLTATYRYILSCHCEECN

**NDP_Chicken**  PSRCMRHHYVDSISHYKCSSKMVLLARCEGRCSQTSRSEPMVSFPFRSTCHCCRPQTSKLKAMRLRCSGGMRLTATYRYILSCHCEECN

**NDP_Lizard**  PVRCMRHHYVDSISHYKCSSKMVLLARCEGRCTQTSRSEPMVSFPFRSTCHCCRPQTSKLKAMRLRCSGGMRLTATYRYILSCHCEECN

**NDP_Frog**  PDRCMRHHYVDSINHHKCTAKMVLLARCEGRCSQTSRSDPLVSFPFRSTCHCCRPQTSKLKAIRLRCSGGRRMTATYRYILSCHCEECN

**NDP_Coelacanth**  PARCMRHHYVDTISHYKCSSKMVLLARCEGRCSQTCRSDPLISFPFKSTCHCCKPKASKLKAVRLRCGSGMRLTATYRYILSCSCERCS

**NDP_Medaka**  PERCVRHHFVETIKHYKCNSKMVLLARCEGPCSHTSRSDPIISFPFKNSCSCCRPHTSKLKAVRLRCSEGTRITATYRYILACNCEECS

**NDP_Fugu**  PGRCMRHHFVETITHYKCNFKMVLLACCEGHCNRTTRSDPLISFPFKSSCSCCRPHTSKLKAVRLRCTGGRRITATYRYILTCNCEECS

**NDP_Zebrafish**  PDRCMRHHFVETITHYKCNSKMVLLARCEGHCSHTSRSDPLISFPFKNTCFCCRPHTSKLKAVRLRCSGGTRITATYRYILACSCEECS

**NDP_Amphioxus**  NSRCKRFHYVQTIRHLDCEVQNVLMARCEGACRRSSRTDPIIEFPFQYRRQSCQAKLSKLKAVRLKCTDGTFVTASYRYITKCACAACP

**NDP_AcornWorm**  ETKCMRYYYTQSITHKKCNDKIVLMSRCSGSCEDTSSTDPVVTFPFKYKCQSCQDQQSNIKAVRLRCRKNQFAYATYRYILSCGCAVCK

**NDP_Urchin**  HPKCVRYYHTERISHKPCQSKIILMSRCAGQCEMASSADPVVSFPFKYRCQSCQDHISIMKAVLLRCQGNERVYATYRYILSCECASCK

**NDP_Oyster**  ENRCMKHYFVHTIEHKNCEKKHILLARCKGFCSKSKTE-PRVTFPFNYHCKCCRDSLSIMKAVSLNCEGDKPVFATYRYILKCKCRNCN

**NDP_Limpet**  --RCMRHYFVETIRHRDCEHKNVLLARCEGSCQKSKTI-PRISFPFKYSCPCCRDALSIMKAVPLKCKNKRRVFATYRYIIQCACHYCG

**NDP_Coral**  KEICKRKPV----SMNVCGGREIRAYKCEGACESESKILMGDP-WFRAECRCCKSIRTET--KSVPCPGGDE---KIRFIHACGCGNCN

**Grem1_Human**  RDWCKTQPLKQTIHEEGCNSRTIINRFCYGQCNSFYIPRHIRKEGSFQSCSFCKPKKFTTMMVTLNCPELQPKKKRVTRVKQCRCISID

**Grem1_Chicken**  RDWCKTQPLKQTIHEEGCNSRTIINRFCYGQCNSFYIPRHVRKEGSFQSCSFCKPKKFTTMTVTLNCPELQPKKKRITRVKECRCISID

**Grem1_Lizard**  RDWCKTQPLKQTINEEGCNSQTIINRFCYGQCNSFYIPRHVQKEGAFQSCSFCKPKKVTTMLVTLNCPELQPKRKRITRVKECRCISID

**Grem1_Frog**  RDWCKTQPLKQTIHEDGCNSRTIINRFCYGQCNSFYIPRHIRREGSFQSCSFCKPKKFTTMVVTLNCPELQPKKKRITRVKQCRCISID

**Grem1_Coelacanth**  RDWCKTQPLKQTIHEEGCNSRTIINRFCYGQCNSFYIPRHVRKEGSFQSCSFCKPRKFTTMTVTLNCPELQPKKKKITRVKQCRCISID

**Grem1_Medaka**  RDWCKTQPLKQTIHEEGCISRTIINRFCYGQCNSFYIPRHIRKEGAFQSCSFCKPKRFTAMTFTLNCPDQQPKRKRIMRVKQCRCISID

**Grem1_Fugu**  RDWCKTQPLKQTIHEEGCVSRTIINRFCYGQCNSFYIPRHIRREGAFQSCSFCKPKRFTTMTFTLNCPDQQPKKKRIQRVKQCRCISID

**Grem1_Zebrafish**  RDWCKTQPLKQTIHEEGCISRTIINRFCYGQCNSFYIPRHVRREGAFQSCSFCKPKRFTTMSFTLSCPDQQPRKKRVQRVKQCRCISIE

**Grem_Amphioxus**  QDWCKTQPLRQTVRAKGCLSRTVINRFCYGQCNSFYIPKHVRKDESFQSCAFCKPHRYSMITVTLRCPSLTPKRKRIQRVKKCKCMSVI

**Grem_Butterfly**  EDWCKTEQLIQKIREPGCLQATVINNFCYGQCNSFYIPKGPRRRPAFKSCSFCKPKKFTWITVTLRCPGQNPRRKRLQKIKQCKCLPVG

**Grem_RedFBeetle** RDWCKTEPLIQKVKEEGCLTRTVINRFCYGQCNSFYIPKNPKKRPAFKACAFCRPSKFTWISVTLKCPSLMPRKKRIQRIKQCKCIAAN

**Grem_Daphnia**  KDWCQSQPLIQRIGQDQCLSATVLNRFCYGQCNSFFIPKNQLKVKAFRSCAVCQPKKTSWVTVTLKCPSLVPRRRRVLIINQCRCM---

**Grem_Anemone**  GDWCKLRPVLQKLHHPGCNSSFIMNNMCYGQCMSFFIPRH------FTSCAFCTPVSKNVVSVHLKCAGDLKVVKKVSIIQSCSCRPCG

**Grem_Coral**  PDACRAYSFKEKIRHRGCNNVTIENNMCYGQCNSFYIPKR------FVSCSYCAPSRMEIYEIRLECPGQNPVLKKVPIVKECACKDCG

**NBL1_Human**  SAWCEAKNITQIVGHSGCEAKSIQNRACLGQCFSYSVPNTFPQSESLVHCDSCMPAQSMWEIVTLECPGHEEVDKLVEKILHCSCQACG

**NBL1_Chick**  SAWCEAKNITQIVGHSGCESKSIQNRACLGQCFSYSVPNTFPQSESLVHCDSCMPAQSMWEIVTLDCPGNDEVDKLVEKILHCSCQACG

**NBL1_Lizar**  SAWCEAKNITQIVGHSGCEAKSIQNRACLGQCFSYSVPNTFPQSESLVHCDSCMPAQSLWEIVTLDCPSNEEVDKLVEKILHCSCQACG

**NBL1_Frog**  SAWCEAKNITQIVGHSGCESKSIQNRACLGQCFSYSVPNTFPQSESLVHCDSCMPIDSVWDVVTLECPGNEEVDKLVEKILQCSCQACG

**NBL1_Coela**  SAWCEAKNITQIVGHSGCESKSIQNRACLGQCFSYSVPNTFPQSESLIHCDSCMPATSMWEIVTLECPGNEEVDKLVEKILRCSCQACG

**NBL1_Medak**  SAWCEAKNITQIVGHTGCQPRSIQNRACLGQCFSYSVPNTFPQSESLVHCDSCMPAQTQWEVVTLNCPGSEEVDKLVERIFHCSCQSCS

**NBL1_Fugu**  SAWCEAKNITQIVGHTGCLPQSIQNRACLGQCFSYSVPNTFPQSESLVHCDSCMPAQTQWEVVTLDCPDSP-VDKLVERIFHCSCQSCS

**NBL1_Zebra**  SAWCEAKNITQIVGHTGCTPRSIQNRACLGQCFSYSVPNTFPQSESLVHCDSCMPAQTQWEVVTLDCSGSDEVDKLVERILHCSCQSCS

**NBL1_Coral**  KSWCRSVEIQHSIQEPGCETKVIENRACMGQCFSYYAPGTHPRKKRMKYCDMCKPSLKSWTKVSLDCPGTNHVDKLVEVIYSCTCQKCI

**NBL1_Placozoan**  KAWCKLAKIEQVLSHPGCISKTISNHICVGQCYSYRIPKSYPPEENLQHCECCHVVDHTWNTVELKCPTLKNVDKLVQYIRSCDCRRCH

**SOSD1_Chicken**  QVGCRELRSTKYISDGQCTSNPLKELVCAGECLPLPLLPGGGYGRRSSQEWRCVNDKTRTQRIQLQCQDGSIRTYKITVVTACKCKRYT

**SOSD1_Human**  QVGCRELRSTKYISDGQCTSSPLKELVCAGECLPLPVLPGGGYGRRSSQEWRCVNDKTRTQRIQLQCQDGSTRTYKITVVTACKCKRYT

**SOSD1_Lizard**  QVGCRELRSTKYISDGQCTSNPLKELVCAGECLPLPVLPGGGYGRRSSQEWRCVNDKTRTQRIQLQCQDGSTRTYKVTVVTACKCKRYT

**SOSD1_Frog**  QVGCRELRSTKYISDGQCTSQPLKELVCAGECLPLPILPGGGYGRRSSQEWRCVNDKTRTQRIQLQCEDGTTRTYKVTVLTSCKCKRYT

**SOSD1_Coelacanth**  QVGCRELRSTKYISDGQCTSNPVKELVCAGECLPAQMLPGGGYGRRNAQEWRCVTDKTRTQRIQLQCQDGSTRTYKITVVTSCKCKRYT

**SOSD1_Medaka**  HVGCRELRSTKYISDGHCTSNPIKELVCAGECLPAQMLWGARGRRSGNNDWRCVNDKTRTQRIQLQCQDGTTRTYKITVVTSCKCKRYS

**SOSD1_Zebrafish**  PVGCRELRSTKYISDGQCTSNPVKGLVCTGQCLPAQMLPIGGYGRRNSQEWRCVNDKTRTQRIQLQCQDGSTRTYKITVVTSCKCKRYS

**SOSD_AcornWorm**  QVGCTELRSKRYISDGFCTSKPIAEVVCAGECVPTRILPFYSDYRTKTREWRCVNDEERYKRVHLQCSNGNTRTYRIKTVRSCKCKRYS

**SOSD_Urchin**  QVHCVELRSKRYISDGFCTSRPITEVVCAGSCIPEDYTGWYFEHNKKRKEWRCVNDSVRQKKVTLLCDNNQTRTYRIKTVRSCKCKRLL

**SOSD_Limpet**  QLGCTELRAKRYISDGFCTSKAIKEVVCAGNCLPIRDIPWYAEFRDKYRDFQCEEDVVRRKKVRLLCQNGEYRSYKIKVVKSCKCKRMR

**SOSD_Aplysia**  QVGCRELRARRYITDGFCTSKPVKEVVCAGNCLPIMDMPWYAEFRTKLRDWQCVEDVVKKKRVKLLCLNGDIRVYRIKVAKSCKCKKIS

**SOSD_SeaAnemone**  NLGCVGIRIKRYVSNGFCTSRAIKDMICEGRCLPMDELPFFPDYSATKREWRCVPDRKRRRNVNVTCNDGTKRKYRILVVRSCKCKRYT

**SOSD_Coral**  NLGCTGIRFRRYVSNGFCTSRPLRDMICDGDCLPMDQLPFFPNFSRHKREWRCVADDRRTKKVKLICNDGTMRKYRVSVVRSCKCKRYT

**SOSD_Placozoan**  SSCCDGKKIHRYVMKGLCISRSIKDLLCEGACRPNNIPQDVS--ASKRYIWRCVAHFRKTIRVKMNCFDGTTKYAFPSVVRKCVCKRYR

**BMP2_Human**  KSSCKRHPLYVDFSDVGWAPPGYHAFYCHGECPFPLA-DHLNTHNSKIPKACCVPTELSAISMLYLDENEKVKNYQDMVVEGCGCR---

**BMP2_Zebrafish**  RSNCRRHALYVDFSDVGWAPPGYHAFYCHGECPFPLP-DHLNTHNSNIPKACCIPTELSPISLLYLDEYEKVKNYQDMVVEGCGCR---

**NODAL_Human**  SQLCRKVKFQVDFNLIGWYPKQYNAYRCEGECPNPVG--EFHNAPHRVPSTCCAPVKTKPLSMLYVDN-GRVDHHKDMIVEECGCL---

**NODAL_Zebrafish**  KPLCKKVDMWVDFDQIGWYPKRYNAYRCEGSCPTPVD--TFTNAPDRVPCLSCVPTRLAPLSMLYYEN-GKMRHHEGMVVAECGCH---

**NODAL_Capitella**  RESCQIKDLIVDFKVIGWYPLSFNAYQCDGKCPLPVVQD-YDNARDSTTRPCCVPTKLKPLSMLYYEK-DMIRHHEDMVVDRCGCR---

**DPP_Capitella**  HPRCRRRQLSVHFEDVGWAPPGYEAYYCQGECPSHLS-DHLNNASQAVPKPCCVPTDLLPISMLYVDEYGEVKSYQDMVVIACGCR---

**DPP_FruitFly**  DDTCRRHSLYVDFSDVGWAPLGYDAYYCHGKCPFPLA-DHFNNAPGKVPKACCVPTQLDSVAMLYLNDQSTVKNYQEMTVVGCGCR---

**DPP_RedFBeetle** KDPCRRRQMYVDFGSVGWAPLGYDAYYCGGECEYPIP-DHMNNAPKEVPGPCCVPTQLGQMSMLYLGSDGSVKNYKEMVVVGCGCR---

**GBB_Marine**  RQSCGRRRDLGWQCRLRLAPDGYSAYYCHGECAFPLN-AHMNNAQLAVPKPCCAPTKLSAISVLYFDDNSNVKKYKNMVVRACGCH---

**GBB_FruitFly**  TRSCQMQTLYIDFKDLGWAPEGYGAFYCSGECNFPLN-AHMNNAPKKVPKPCCAPTRLGALPVLYHLNDENVKKYRNMIVKSCGCH---

**GBB_RedFBeetle** GSTCKMYDLYISFKDLKWAPAGYSAHYCAGECKFPLN-GHMNNAPNKYPKPCCAPTKLTPISVLYFQDDTNVKKYKKMSVKSCGCH---

**TGF_CombJelly**  NYHCRRQDLWINFDEIGWTPKDVNIGDCGGQCLDADSN----PAPARAGILCCQGANYKEISALYFKK-HEIGDMPKIIVKECRCR---

**TGF_Sponge1**  PGACRL-QFHHVNLSDYFEPSQYSISYCAGTCDSAPDV----TTPATVPPLCCVPERYTSLTVLVQRD-DTTETLLNFAAKSCHCV---

**TGF_Sponge3**  EDVCKRHPLLVNFTDVGWHPPVVDIGVCSGVCGSLEKVYHAEALRKQKPGPSCTPMTFKAIKVKRRLG-NTTGVIPRLVVESCMCS---

**Fig. S2D.** Sequences used in the alignments presented in Figures 1B, 1C, S1 and S2C.

| **Peptide** | **Common Name** | **Species** | **Accession** | **Database** |
| --- | --- | --- | --- | --- |
| B/GPH | Placozoan | *Trichoplax adhaerans* | N/A (fgenesh from scaffold 31) | JGI |
| B/GPH1 | Coral | *Acropora digitifera* | adi_v1.00854 | OIST |
| B/GPH1 | Hydra | *Hydra magnipapillata* | XP_002156629.1 | NCBI |
| B/GPH1 | Sea Anemone | *Nematostella vectensis* | XP_001636257.1 | NCBI |
| B/GPH2 | Coral | *Acropora digitifera* | adi_v1.15298 | OIST |
| B/GPH2 | Hydra | *Hydra magnipapillata* | DT608554.1 | NCBI |
| B/GPH2 | Sea Anemone | *Nematostella vectensis* | XP_001629625.1 | NCBI |
| BMP | Oyster | *Crassostrea gigas* | EKC42418.1 | NCBI |
| BMP2 | Human | *Homo sapiens* | NP_001191.1 | NCBI |
| BMP2 | Zebrafish | *Danio rerio* | NP_571435.1 | NCBI |
| Bursα | Daphnia | *Daphnia pulex* | EFX87546.1 | NCBI |
| Bursα | Fruit Fly | *Drosophila melanogaster* | NP_650983.1 | NCBI |
| Bursα | Red Flour Beetle | *Tribolium castaneum* | NP_001107779.1 | NCBI |
| Bursα | Silkworm | *Bombyx mori* | NP_001091845.1 | NCBI |
| Bursα | Urchin | *Strongylocentrotus purpuratus* | NP_001103719.1 | NCBI |
| Bursα | Wasp | *Nasonia vitripennis* | NP_001155852.1 | NCBI |
| Bursβ | Acorn Worm | *Saccoglossus kowalevskii* | XP_002732831.1 | NCBI |
| Bursβ | Daphnia | *Daphnia pulex* | EFX87749.1 | NCBI |
| Bursβ | Fruit Fly | *Drosophila melanogaster* | NP_609712.1 | NCBI |
| Bursβ | Red Flour Beetle | *Tribolium castaneum* | NP_001107780.1 | NCBI |
| Bursβ | Silkworm | *Bombyx mori* | NP_001037289.1 | NCBI |
| Bursβ | Urchin | *Strongylocentrotus purpuratus* | NP_001103717.1 | NCBI |
| Bursβ | Wasp | *Nasonia vitripennis* | XP_001601162.1 | NCBI |
| CKHa1 | Comb Jelly | *Mnemiopsis leidyi* | MLRB018049 | NHGRI |
| CKHa1 | Sponge 1 | *Ephydatia muelleri* | comp31895 | Compagen |
| CKHa2 | Comb Jelly | *Mnemiopsis leidyi* | ML2835 | NHGRI |
| CKHa2 | Sponge 1 | *Ephydatia muelleri* | comp41664 | Compagen |
| CKHb1 | Sponge 1 | *Ephydatia muelleri* | comp73214 | Compagen |
| CKHb1 | Sponge 3 | *Oscarella carmela* | m.227990 | Compagen |
| CKHb2 | Sponge 1 | *Ephydatia muelleri* | comp71092 | Compagen |
| CKHb2 | Sponge 3 | *Oscarella carmela* | m.51490 | Compagen |
| DPP | Annelid Capitella | *Capitella teleta* | ELU12862.1 | NCBI |
| DPP | Fruit Fly | *Drosophila melanogaster* | NP_477311.1 | NCBI |
| DPP | Red Flour Beetle | *Tribolium castaneum* | NP_001034540.1 | NCBI |
| GBB | Annelid Capitella | *Capitella teleta* | ELU11942.1 | NCBI |
| GBB | Fruit Fly | *Drosophila melanogaster* | NP_477340.1 | NCBI |
| GBB | Red Flour Beetle | *Tribolium castaneum* | NP_001107813.1 | NCBI |
| GPHα2 | Acorn Worm | *Saccoglossus kowalevskii* | CAR94705.2 | NCBI |
| GPHα2 | Annelid Capitella | *Capitella teleta* | 222558 | JGI |
| GPHα2 | Aplysia | *Aplysia californica* | NP_001191641.1 | NCBI |
| GPHα2 | Daphnia | *Daphnia pulex* | EFX79541.1 | NCBI |
| GPHα2 | Frog | *Xenopus tropicalis* | XP_002937557.1 | NCBI |
| GPHα2 | Fruit Fly | *Drosophila melanogaster* | NP_001104054.2 | NCBI |
| GPHα2 | Human | *Homo sapiens* | NP_570125.1 | NCBI |
| GPHα2 | Lamprey | *Petromyzon marinus* | CBX24528.1 | NCBI |
| GPHα2 | Limpet | *Lottia gigantea* | 161605 | JGI |
| GPHα2 | Lizard | *Anolis carolinensis* | XP_003229755.1 | NCBI |
| GPHα2 | Louse | *Pediculus humanus* | XP_002427818.1 | NCBI |
| GPHα2 | Medaka | *Oryzias latipes* | XP_004079816.1 | NCBI |
| GPHα2 | Oyster | *Crassostrea gigas* | EKC24491.1 | NCBI |
| GPHα2 | Red Flour Beetle | *Tribolium castaneum* | NP_001164244.1 | NCBI |
| GPHα2 | Silkworm | *Bombyx mori* | NP_001124375.1 | NCBI |
| GPHα2 | Urchin | *Strongylocentrotus purpuratus* | XP_003725463.1 | NCBI |
| GPHα2 | Zebrafish | *Danio rerio* | NP_001160156 | NCBI |
| GPHβ5 | Acorn Worm | *Saccoglossus kowalevskii* | NP_001161671.1 | NCBI |
| GPHβ5 | Annelid Capitella | *Capitella teleta* | 157108 | JGI |
| GPHβ5 | Aplysia | *Aplysia californica* | NP_001191597.1 | NCBI |
| GPHβ5 | Chicken | *Gallus gallus* | XP_003641489.1 | NCBI |
| GPHβ5 | Daphnia | *Daphnia pulex* | EFX79646.1 | NCBI |
| GPHβ5 | Frog | *Xenopus tropicalis* | XP_002939523.1 | NCBI |
| GPHβ5 | Fruit Fly | *Drosophila melanogaster* | NP_001104335.1 | NCBI |
| GPHβ5 | Fugu | *Takifugu rupribes* | XP_003978682.1 | NCBI |
| GPHβ5 | Human | *Homo sapiens* | NP_660154.3 | NCBI |
| GPHβ5 | Lamprey | *Petromyzon marinus* | CAR95358.1 | NCBI |
| GPHβ5 | Lizard | *Anolis carolinensis* | XP_003214333.1 | NCBI |
| GPHβ5 | Louse | *Pediculus humanus* | CAR95343.1 | NCBI |
| GPHβ5 | Medaka | *Oryzias latipes* | XP_004079817.1 | NCBI |
| GPHβ5 | Oyster | *Crassostrea gigas* | EKC24490.1 | NCBI |
| GPHβ5 | Red Flour Beetle | *Tribolium castaneum* | CAR95345.1 | NCBI |
| GPHβ5 | Silkworm | *Bombyx mori* | NP_001124380.1 | NCBI |
| GPHβ5 | Urchin | *Strongylocentrotus purpuratus* | XP_003725461.1 | NCBI |
| GPHβ5 | Zebrafish | *Danio rerio* | NP_001159810.1 | NCBI |
| Grem | Amphioxus | *Branchiostoma floridae* | XP_002604466.1 | NCBI |
| Grem | Butterfly | *Danaus plexippus* | EHJ70788.1 | NCBI |
| Grem | Coral | *Acropora digitifera* | adi_aug_v2a.20502 | OIST |
| Grem | Daphnia | *Daphnia pulex* | EFX89983.1 | NCBI |
| Grem | Hydra | *Hydra magnipapillata* | XP_002160711.1 | NCBI |
| Grem | Placozoan | *Trichoplax adhaerans* | N/A (fgenesh from scaffold 31) | JGI |
| Grem | Red Flour Beetle | *Tribolium castaneum* | XP_973724.1 | NCBI |
| Grem | Sea Anemone | *Nematostella vectensis* | ABF06563.1 | NCBI |
| Grem1 | Chicken | *Gallus gallus* | NP_990309.1 | NCBI |
| Grem1 | Coelacanth | *Latimeria chalumnae* | ENSLACP00000007025 | ENSEMBL |
| Grem1 | Frog | *Xenopus tropicalis* | NP_001093701.1 | NCBI |
| Grem1 | Fugu | *Takifugu rupribes* | XP_003962645.1 | NCBI |
| Grem1 | Human | *Homo sapiens* | NP_037504.1 | NCBI |
| Grem1 | Lizard | *Anolis carolinensis* | XP_003214534.1 | NCBI |
| Grem1 | Medaka | *Homo sapiens* | XP_004082395.1 | NCBI |
| Grem1 | Zebrafish | *Danio rerio* | XP_001344700.2 | NCBI |
| NBL1 | Chicken | *Gallus gallus* | NP_989480.1 | NCBI |
| NBL1 | Coelacanth | *Latimeria chalumnae* | ENSLACP00000011728 | NCBI |
| NBL1 | Coral | *Acropora digitifera* | adi_EST_assem_13939 | OIST |
| NBL1 | Frog | *Xenopus tropicalis* | NP_001006826.1 | NCBI |
| NBL1 | Fugu | *Takifugu rupribes* | XP_003973311.1 | NCBI |
| NBL1 | Human | *Homo sapiens* | NP_877421.2 | NCBI |
| NBL1 | Lizard | *Anolis carolinensis* | XP_003229759.1 | NCBI |
| NBL1 | Medaka | *Oryzias latipes* | XP_004068503.1 | NCBI |
| NBL1 | Placozoan | *Trichoplax adhaerans* | XP_002113818.1 | NCBI |
| NBL1 | Zebrafish | *Danio rerio* | NP_996980.1 | NCBI |
| NDP | Acorn Worm | *Saccoglossus kowalevskii* | GL016179.1 | NCBI |
| NDP | Amphioxus | *Branchiostoma floridae* | XP_002592912.1 | NCBI |
| NDP | Chicken | *Gallus gallus* | XP_416765.1 | NCBI |
| NDP | Coelacanth | *Latimeria chalumnae* | ENSLACP00000013174 | ENSEMBL |
| NDP | Coral | *Acropora digitifera* | adi_v1.15299 | OIST |
| NDP | Frog | *Xenopus tropicalis* | XP_002932837.1 | NCBI |
| NDP | Fugu | *Takifugu rupribes* | XP_003961645.1 | NCBI |
| NDP | Human | *Homo sapiens* | NP_000257.1 | NCBI |
| NDP | Limpet | *Lottia gigantea* | 78012 | JGI |
| NDP | Lizard | *Anolis carolinensis* | XP_003218984.1 | NCBI |
| NDP | Medaka | *Oryzias latipes* | XP_004081655.1 | NCBI |
| NDP | Oyster | *Crassostrea gigas* | EKC33738.1 | NCBI |
| NDP | Urchin | *Strongylocentrotus purpuratus* | GL905581.1 | NCBI |
| NDP | Zebrafish | *Danio rerio* | XP_001338820.1 | NCBI |
| NGF | Aplysia | *Aplysia californica* | NP_001240690.1 | NCBI |
| NGF | Red Flour Beetle | *Tribolium castaneum* | EEZ98286.1 | NCBI |
| NGF | Urchin | *Strongylocentrotus purpuratus* | NP_001073024.1 | NCBI |
| NGFB | Human | *Homo sapiens* | NP_002497.2 | NCBI |
| NODAL | Annelid Capitella | *Capitella teleta* | ELT90528.1 | NCBI |
| NODAL | Human | *Homo sapiens* | NP_060525.3 | NCBI |
| NODAL | Zebrafish | *Danio rerio* | NP_571041.1 | NCBI |
| PDGF | Coral | *Acropora digitifera* | adi_v1.03319 | OIST |
| PDGF | Hydra | *Hydra magnipapillata* | XP_002160342.2 | NCBI |
| PDGF | Oyster | *Crassostrea gigas* | EKC18709.1 | NCBI |
| PDGF | Red Flour Beetle | *Tribolium castaneum* | XP_001814264.1 | NCBI |
| PDGF | Sea Anemone | *Nematostella vectensis* | XP_001631490.1 | NCBI |
| PDGF | Urchin | *Strongylocentrotus purpuratus* | XP_001184357.2 | NCBI |
| PDGFA | Human | *Homo sapiens* | NP_002598.4 | NCBI |
| SOSD | Acorn Worm | *Saccoglossus kowalevskii* | NP_001161651.1 | NCBI |
| SOSD | Aplysia | *Aplysia californica* | EB259655.1 | NCBI |
| SOSD | Coral | *Acropora digitifera* | adi_v1.16523 | OIST |
| SOSD | Limpet | *Lottia gigantea* | 88167 | JGI |
| SOSD | Placozoan | *Trichoplax adhaerans* | XP_002110162.1 | NCBI |
| SOSD | Sea Anemone | *Nematostella vectensis* | XP_001629632.1 | NCBI |
| SOSD | Urchin | *Strongylocentrotus purpuratus* | XP_003724979.1 | NCBI |
| SOSD1 | Chicken | *Gallus gallus* | NP_989704.1 | NCBI |
| SOSD1 | Coelacanth | *Latimeria chalumnae* | ENSLACP00000012342 | ENSEMBL |
| SOSD1 | Frog | *Xenopus tropicalis* | NP_001093740.1 | NCBI |
| SOSD1 | Human | *Homo sapiens* | NP_056279.1 | NCBI |
| SOSD1 | Lizard | *Anolis carolinensis* | XP_003222059.1 | NCBI |
| SOSD1 | Medaka | *Oryzias latipes* | XP_004078458.1 | NCBI |
| SOSD1 | Zebrafish | *Danio rerio* | NP_001017598.1 | NCBI |
| TGF | Comb Jelly | *Mnemiopsis leidyi* | AEP16389.1 | NCBI |
| TGF | Sponge 1 | *Ephydatia muelleri* | comp49207 | Compagen |
| TGF | Sponge 3 | *Oscarella carmela* | g65.t1/m.310560 | Compagen |
| TGF | Urchin | *Strongylocentrotus purpuratus* | XP_793246.2 | NCBI |
| TGFB1 | Human | *Homo sapiens* | NP_000651.3 | NCBI |

**Fig. S3A.** Maximum-likelihood tree (LG+G+F) of LGR sequences.


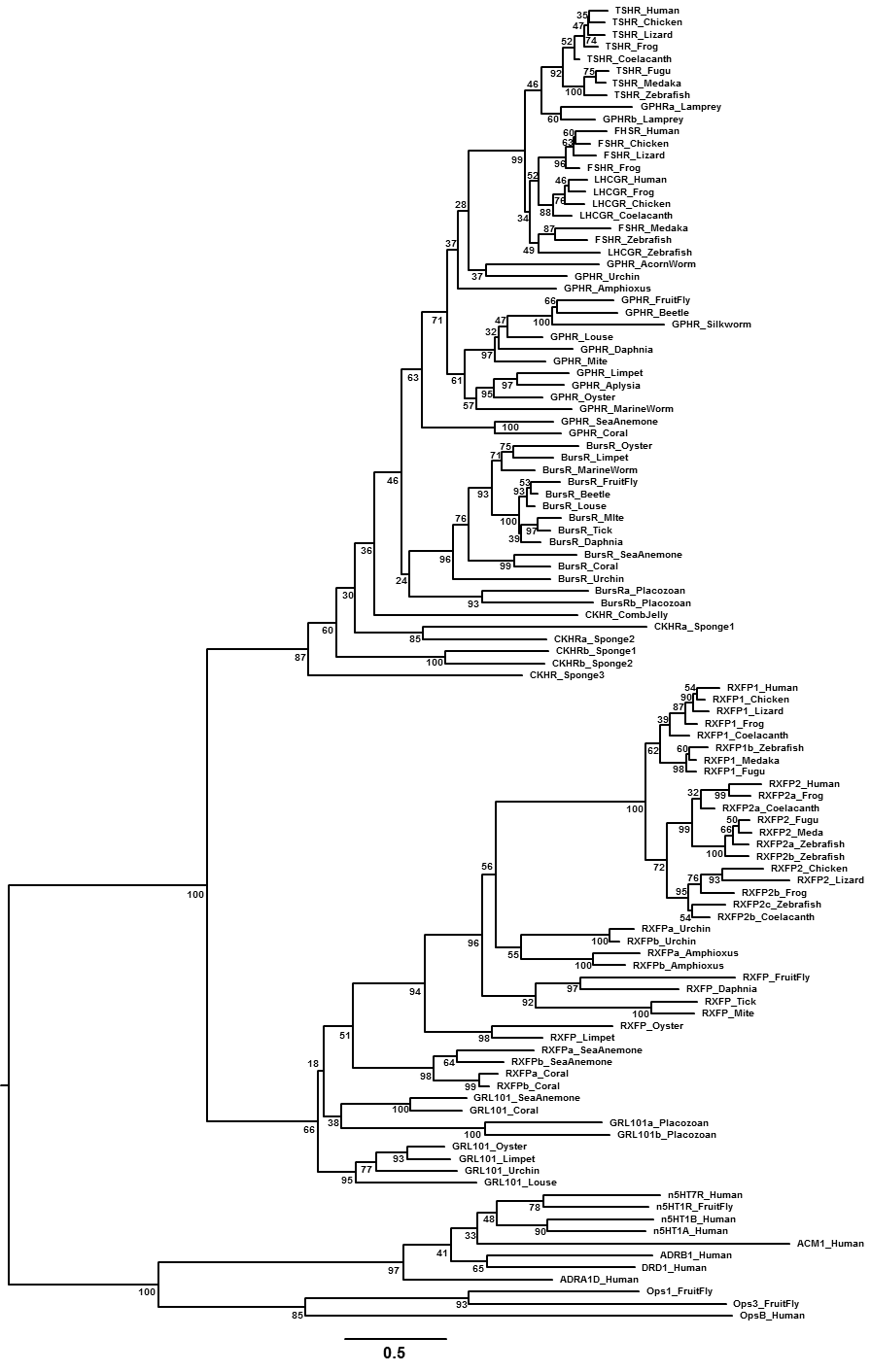


**Fig. S3B.** Bayesian inference tree (LG+G) of LGR sequences.

**
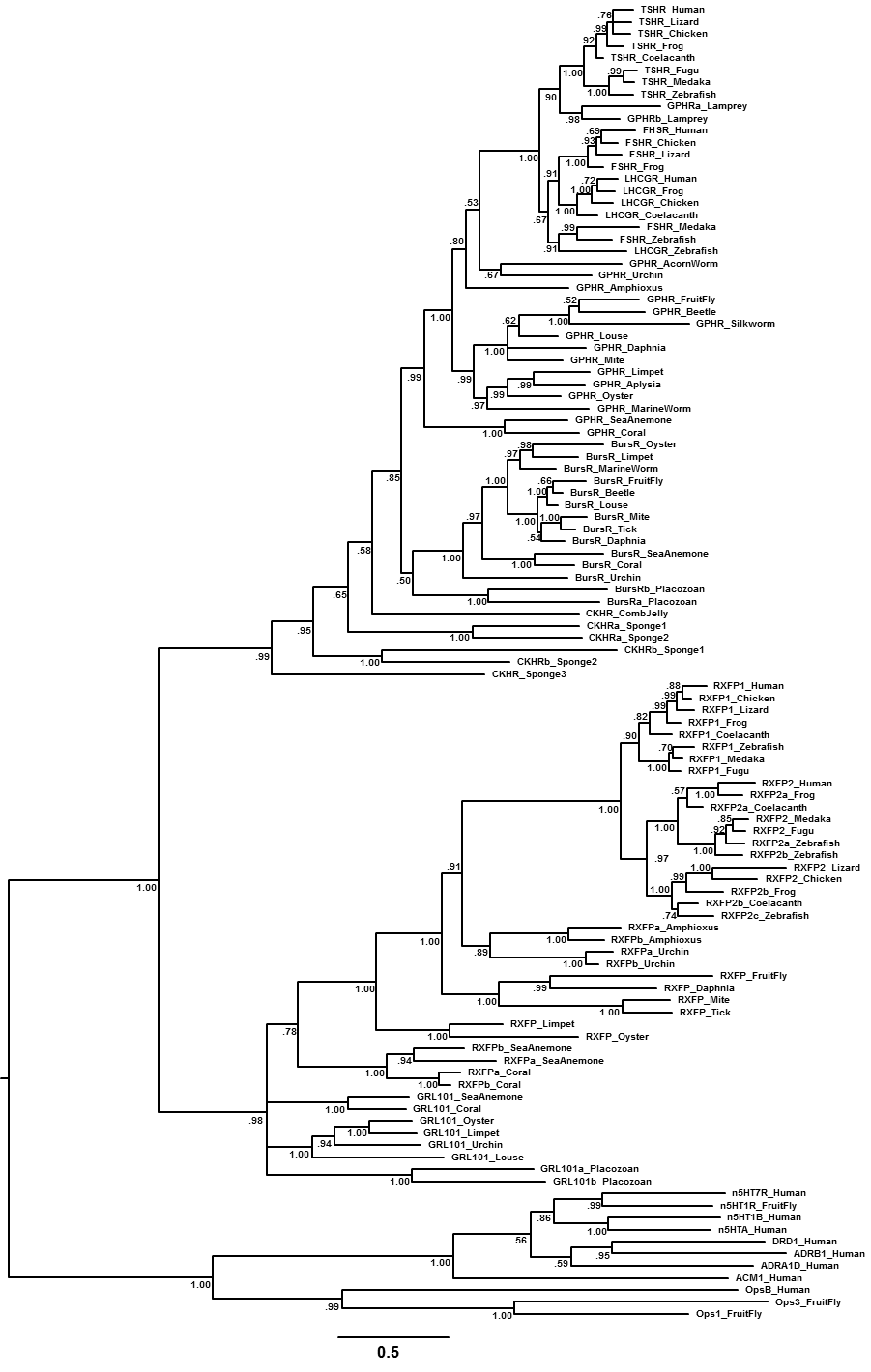
**

**Fig. S3C.** Alignment of LGRs used to produce phylogenetic trees in figs. 3, S3A and S3B.

10 20 30 40 50 60 70 80 90 100 110 120 130 140 150 160

....|....|....|....|....|....|....|....|....|....|....|....|....|....|....|....|....|....|....|....|....|....|....|....|....|....|....|....|....|....|....|....|

**FHSR_Human**  GNIIVLVILTTSQYKLTVPRFLMCNLAFADLCIGIYLLLIASVDIHTKSQYHNYAIDWQTGAGCDAAGFFTVFASELSVYTLTAITLERWHTITHAMQLDKVQLRHAASVMVMGWIFAFAAALFPIFGISSYMKVSICLPMDIDSPLSQLYVMSLLVLNV

**FSHR_Chicken**  GNTTVLIILISSQYKLTVPRFLMCNLAFADLCIGIYLLFIASVDIQTKSRYYNYAIDWQTGAGCNAAGFFTVFASELSVYTLTVITLERWHTITYAMQLNKVRLRHAVIIMVFGWMFAFTVALLPIFGISSYMKVSICLPMHIETPFSQAYVIFLLVLNV

**FSHR_Frog**  GNSVVLIILLTSQYKFTVPRFLMCNLAFADLCMGVYLLLIAAVDIKTKSQYYNYAIDWQTGAGCHAAGFFTVFASELSVFTLTVITLERWHTITYAMQLDKVRFRHATMIMASGWVFSFTVALLPIVGVSSYMKVSICLPMDIENPLSQAYIMFLLVLNV

**FSHR_Lizard**  GNLVVFIILVSSQYKLTVPRFLMCNLAFADLCTGIYLLLIAIKDVQTRSQYYNYAIDWQTGAGCNTAGFFTVFASELSIYTLTVITLERWHTITYAMELDKVRFRHAVVIMLVGWVFAFTIALLPIFEVSSYMKVSICLPMDIETPLAQAYIMFLLVLNI

**FSHR_Medaka**  GNTVVLVVLLGSRSKLTVPRFLMCHLAFADLCMGIYLIIIATIDMLTHGHYYNYAIDWQTGLGCSAAGFFTVFASELSVFTLTAITVERWHTITHALRLDKLRMRHACVIMAAGWIFSSLAALLPTVGVSSYSKVSVCLPMDVESLVSQVYLVSLLLLNI

**FSHR_Zebrafish**  GNTVVLLVLLTSRYKLTVPRFLMCHLAFADLCMGIYLLLIAAVDIHTQSRYYNYGIDWQTGAGCHVAGFFTVFSSELSVYTLTAITLERWHTITYAMQLEQMRLRHACLVMATGWLFSLLAALMPMFGVSSYSKTSICLPMDVETLLSQGYVVLLLLLNA

**LHCGR_Human**  GNMTVLFVLLTSRYKLTVPRFLMCNLSFADFCMGLYLLLIASVDSQTKGQYYNHAIDWQTGSGCSTAGFFTVFASELSVYTLTVITLERWHTITYAIHLDKLRLRHAILIMLGGWLFSSLIAMLPLVGVSNYMKVSICFPMDVETTLSQVYILTILILNV

**LHCGR_Chicken**  GNFIVLLVLITSHYKLTVPRFLMCNLSFADFCMGLYLLLIASVDAQTSGQYYNHAIDWQTGSGCSTAGFFTVFASELSVYTLTVITIERWHTITYAMQLDKLRLRHAVPIMLGGWVFSILIAVLPLLGVSSYMKVSICLPMDIETGLSQAYILLILMLNV

**LHCGR_Frog**  GNAVVLFILLTSHCKLTVPRFLMCNLSFADFCMGIYLLLIASVDSQTKSKYYNHAIDWQTGSGCSAAGFFTVFASELSVYTLTVITLERWHTITYAMQLDKLRLRHAILIMLGGWIFSLVIAMLPLVGVSNYIKVSICLPMDIETPLSQAYIIFILVLNV

**LHCGR_Coelacanth**  GNIIVLLVLLTSRYKLTVPRFLMCNLSFADLCMGLYLLLIASVDSKTRSQYYNHAIEWQTGAGCNTAGFFTVFASELSVYTLTVITLERWHTITYAMQLENLRLRHAIMIMIGGWIFSLSMALLPIAGISNYMKVSICLPMDIETSVSQAYILIILVLNV

**LHCGR_Zebrafish**  GNLVVLLVLFTSRCKLTVPRFLMCHLAFADLCIGIYLLMIATVDLRTRGHYSHHAIEWQTGAGCDIAGFLSVFGGELSIYTLSTITVERWHTITHALRLERLGLSQASLIMTIGWLLCLAMALLPLIGVSSYSKVSMCLPMDIETPLSQAYVILLLLFNV

**TSHR_Human**  GNVFVLLILLTSHYKLNVPRFLMCNLAFADFCMGMYLLLIASVDLYTHSEYYNHAIDWQTGPGCNTAGFFTVFASELSVYTLTVITLERWYAITFAMRLDKIRLRHACAIMVGGWVCCFLLALLPLVGISSYAKVSICLPMDTETPLALAYIVFVLTLNI

**TSHR_Chicken**  GNVFVLFILLTSHYKLTVPRFLMCNLAFADFCMGLYLLLIASVDLYTRSEYYNHAIEWQTGPGCNTAGFFTVFASELSVYTLTVITLERWYAITFAMRPNKIRLRHALVIMLGGWLSCFLLALLPLVRVSSYSKVSICLPMDTETPVAEAYVVFVLICNI

**TSHR_Lizard**  GNAFVLFILLTSHYKLTVPRFLMCNLAFADFCMGLYLLLIASVDFYTRSEYYNYAIDWQTGSGCSAAGFFTVFGSELSVFTLTAITLERWYAITFAMRLDKIRLWHASVIMLAGWMVCFLLALLPLVGVSSYGKVSICLPMDTETPLDQAYIVFVLLLNI

**TSHR_Frog**  GNVFVLFILITSHYKLTVPRFLMCNLAFADFCMGIYLLLIASVDVHTRSEYYNYAINWQTGPGCNAAGFFTVFASELSVYTLTMITLERWYAITFAMRLDKIRLRHASFIMLGGWTLCFFLALLPLVGISSYIKVSICLPMDTEAALSQAYIVFVLMLNI

**TSHR_Coelacanth**  GNIFVLLILLTSHYKLTVPRFLMCNLAFADFCMGIYLLLIASVDLHTQSEYYNRAIDWQTGPGCNAAGFFTVFASELSVYTLTVITLERWYAITFAMRLDKIRLRHASAIMLGGWLFCFLLAILPLVGVSSYAKVSICLPMDTETLLAQVYVICVLMLNI

**TSHR_Medaka**  GNLVVLLVLLTSHYKLSVSRFLMCHLAFADLCMGIYLLLIASVDLHTRSEYFNHAIDWQTSPGCGLAGFFTVFSSELSIYTLTVITLERWYAITFAMRLDKLHLYHAAVVMLGGWIFCLLLALLPLVGVSSYQKVSICLPMDTQSTAAQIYILSVLVLNI

**TSHR_Fugu**  GNVVVLLVLLTSHYKLSVSRFLMCHLAVADLCMGIYLLLIASVDLHTRAEYFNHAIDWQTGPGCGLAGFFTVFASEFSVYTLTVITLERWYAITFAMRLDKLHLHHAAAVMLGGWIFCLILAVLPLVGVSSYQKVSICLPMDTQSIAAQVYILSVLVLNI

**TSHR_Zebrafish**  GNLLVLLVLLTSHYKLSVSRFLMCHLAVADLCMGIYLLLIASVDLHTQSEYYNHAIDWQTGPGCSLAGFFSVFASELSVYTLTTITLERWYAITYAMRLDKLRLSHASVIMLIGWIFCLLLGLMPLVGVSSYQKVSICLPMDTQTLVDQIYIICVLVLNI

**GPHRa_Lamprey**  GNSIVILVILSSRYKLAVPRFLMCNLAFADLCMGVYLLTIASFDVYTRGEYHNHAIDWQTGLGCRLAGFLTVFSSELSVFTLTVITVERWHTIIYTMRLDKVSSAQAVTIMASPWALALVLAALPLAGISSYSKVSICLPMDIETLPSQAYVQLILGLNI

**GPHRb_Lamprey**  GNLFVLLIILTSHYKPTVPRFLMCNLAFADLCMGIYLLIIAGFDLYSRNEFYNHAIDWQTGPGCNIAGFVSVFASELSVYTLMVITVERWHAITFAMRLDKIRFRQALGVMAGGWLFSVVLAGMPLYGVSSYSKVSICLPMEVETFVAQAYVVTVLALNV

**GPHR_Amphioxus**  GNLLVVLVLLLTKTKMNVAKFLICNLAFADFCLGFYLLMLAAADIYSSHSYYTWAIVWQTGGGCQVAGFLTMFGSVLSIFTLTFITVERWYAITYAIHLDRVTMRLATRVMVLGWIAAIVMGLLPLVGVSSYSWTSICLPMDTTDPHDLGYVATTMIISV

**GPHR_AcornWorm**  GNFIVLVVLLSYISKITVTKFLMCNLAFADFLQGVYLLVTGIVDVVARGQYYNYAIDWQWGAGCKSIGFISIFATCVSVFTLTTITMERWYAIIHAMHLNRLHMKMAAKIMLVGWVFSLSMACLPLFGVSDYSVTSMCLPMDATGALDLVYILTLCTIIT

**GPHR_Urchin**  GNFIVLVVLASQRHKMTVPKFLMCNLSLADLCMGLYLLIVASVDVHTAGEYFNYSIQWQYGAGCSVAGFISMLSSELSVFSLTVITIERWYTIIYAIDLNRIRLRLAGRIMIVGWIFSILIAVLPLFKVGNYGITSMCLPFYIENRVTIAYVVVILFVNT

**GPHR_Oyster**  GNIIVLVVLLSNRKKMTVTKFLMCNLAFADFLMGVYLLLLAAIDIHSLGEYFNFAIAWQNEGGCQTAGFLTVFSSELSVFTLTVITMERWYAISHAIHLTRLKLRQSCVVMAVGWLYAVVMALLPLVGINGYGNVSVCLPMDVTKTGDVVYVISLLVLNG

**GPHR_Limpet**  GNLVVILVIITGGSKMTVPNFLMCNLSVADFLMGLYLLLIASMDVHSLGEYFNHAITWQNDGGCQVAGFLTVFASELSIFVLTVITIERWYAISYAMY-AKLKLRQASLLMLLGWGYATFMASLPLMGISGYGTVSICLPMEANDLLDKSYICSLLILNG

**GPHR_Aplysia**  GNLLVIVVLLTARSKLTVPKFLMCNLSFADLLMGLYLLLIACVDAHTLGEYFTHAVSWQNDGGCQVAGFLTVFSSELSVFVLTIITLERWYAISQAIHVNRLRMRQATVLMCAGWLYALAMAILPLFGVSGYGAVSMCLPMEARDAVDIIYIIALLVFNG

**GPHR_Capitella**  GNLIVLIVTIGRASTFTVAKFLMCNLAFADLLISVYLLMLASFDLHTMGVYFTRAIPWQYGGWCQTAGFLAIFATCLSTFTLTVITMERWYAISYAIHLTRLRLRLACKILLLGWVFALIMAFLPILGVSSYSKTSICLPMEASSAMDKAYVLMLLVINA

**GPHR_FruitFly**  GNVAVLTVILSIRPESPVPRFLMCHLAFADLCLGLYLLLVACIDAHSMGEYFNFAYDWQYGLGCKVAGFLTVFASHLSVFTLTVITIERWLAITQAMYLNRIKLRPAALIMLGGWIYSMLMSSLPLFGISNYSSTSICLPMENRDVYDTIYLIAILGSNG

**GPHR_Silkworm**  GNVAVLLVLLTNHTELTVPRFLMCNLAFSDLCTGLYLLMLAVVDLRSYGEFFNYAYNWQYGVGCKIAGFLSVFSGQLSVITLTIVTLERWFAITYAIYLERISLSTAAKIMLGGWLFSSLMAGLPLLGVSDYSSTSICLPVESKDIGVVIYQGSLFLTNA

**GPHR_RedFBeetle** GNLAVIVVVLFSGGELTVNRFLMCNLAFADFCMGLYLLLIASMDLHSVGTYFNFAFDWQYGFGCKLAGFLTVFSCHLSIFTLTIITLERWFAITYAIHLTRIRLGAAAKTMLGGWMYSILVASLPLVGVSNYSSTSICLPMEVNRVADRAYLYSIILVNA

**GPHR_Louse**  GNLAVLLVLLSSRFRMTVPKFLMCNLAMADFLMGLYLLLIAVMDVRSIGVYFNFAIDWQNGWGCQVAGFLTVLSSELSIFTLTVITCERWYTITYAIHLNRLKLKTAAHIMAGGWIYSIMMAALPLAGVSSYSKTSICLPLENRETGDIAYLITLLIFNG

**GPHR_Daphnia**  GNLAVVLVLVQSRGPMAVSKFLMVNLALADLCMGLYLFLIVGMDVNTIGVYFNYAIDWQNGIGCQVAGFLTVFATELSVFTLVVITSERWYTITYAINLTRLRLSTTAKIVAVGWIGSFTMASLPLVGVSSFSTTSICLPLENHNLSDTIYLLLLLILNG

**GPHR_Mite**  GNLAVMVVLMSSRFRMTVSKFLMCNLALADLCMGLYLLLIAVEDVTTVGSYFNHAIQWQHGAGCKVAGFLTVFASELSIYTLTTITLERWYAITFSIHLNRLKLRTAARIMAFGWGYAILVATLPLVGVSGYSKTSICLPMVNRNTVDVTYLSSLLSFNG

**GPHR_SeaAnemone**  -NFVVALVLLVSERRLNVTRFLMCNLAFADFCLGLYLFILTCVSMDTHGLYHNYVRRWQYGAGCKLTGFLAVFATELSVYTLVLITLERFYAIVYAMQLNRLSMRMTVRAMAAGWVAAVLLASLPLMGASSYSKVAICLPFDVSTTGSIAYVAFLLFLNG

**GPHR_Coral**  -NLTVAFVLIASERRLNVHRFLMSNLAFADFCLGLYIFTLVCVSLNTSGEYYNSVRTWQYGASCQITGFLAVFSTELSVFTLTLITIERFFAIVYAMEINRVSLRKAVKVMVVGWLFAFLVALLPLLGVNDYRSVAICLPFDSDSRNASAYIAIVLVLNF

**BursR_Urchin**  GNATVIFVIFLSHSKMDVPRFLICNLASADFAMGLYLTILASKDAASLGVFRQTAVQWQLGNGCRVAGFLAVFSSELSIYTLTTITLERFYAIKHAMHLERLKLRRAVIIMAIGWIFAITIATLPLLKYGIHYRYAVCLPVKVDDTKSLIYVATIMISNG

**BursR_Oyster**  GNGIVLFVSITARSSMDVPRFLICNLACADFMMGAYLGILAILDASTLDEFQFYAIKWQRSGGCLTAGFLGIVSSMLSVYTLTVITVERFYAISNAMQLNRIRLKQAGIIMTFGWVFSITNASLPYFGISDYRKFAICLPFETSDRASLAYVCFLMLFNA

**BursR_Limpet**  GNGVVLFVSITSRSKMDVPRFLICNLACADFFMGIYLGFLAVVDASTLGEFRKHAIWWQLSPGCLIAGFVGVFSSELSVFTLTVITIERFYAITHAMQLNRLSLRHAGYVMLLGWLYSIILASLPLFGISDYRKFAVCLPFEITDVFSKAYVCYIMIFNG

**BursR_Capitella**  GNGIVLFISIVSHRRMDVPRFLICNLASADFLMGIYLGFLAVVDASTLGEFRKYAIKWQTSPGCQVAGFLGVLSSELSVFTLTVITMERFYAISHAVHMNRLSLRNARYIVSAGWLMAIILAMLPLFGISDYRKFAICLPFETGDSVSLGYVCFIMLVNC

**BursR_FruitFly**  GNGTVVFVLLCSRSKMDVPRFLVCNLAAADFFMGIYLGILAIVDAATLGEFRMFAIPWQMSVLCQLSGFLAVLSSELSVYTLAVITLERNYAITHAIHLNRLSLKQAGYIMSVGWVFALIMALMPLVGVSDYRKFAVCLPFETTTPASLTYVISLMFING

**BursR_RedFBeetle** GNGTVVFVLIFSRGKMDVPRFLVCNLAAADFFMGIYLGFLAVVDASTLGEFRMYAIPWQMSAGCQLSGFLGVLSSELSVYTLAVITLERNYAITHAMHLNRLSLKHAGYIMICGWSFAIVMGLLPLFSVSDYRKFAVCLPFETKDAASLTYVVFLMFING

**BursR_Louse**  GNGTVVFVLIFSRSKMDVPRFLVCNLAAADFFMGIYLGFLAVVDASTLGEFRMYAIPWQMSVGCQLAGFLGVLSSELSVYTLAVITLERNYAITHAMHLNRLSLKHAGYIMACGWSFALIMSLFPLFGVSDYRKFAICLPFETETPVSLAYVVFLMLING

**BursR_Daphnia**  GNGAVVFVLIFARSKIDVPRFLVCNLGMADFFMGIYLGFLAVVDASTLGEFRMYAIPWQLSAGCQVAGFFGVLSSELSVFTLAVITMERNYAITHAMHLNRLSLRHASYIMAAGWLFALIMAFLPLVGVSDYRKFAVCLPFEVGNSVSKGYVVFLMVING

**BursR_Mite**  GNGLVVVVLSFGRSKIDVPRFLVCNLAMADFLMGIYLGFLAVVDASTLNEFKFYGVAWQTSFGCQLAGFFGVLSSELSVYTLAVITMERNYAITHAMHLNRLSLKHATYIMSIGWSFALIMAILPLFGISDYRKFAVCLPFETGDQASMTYVVSLIAVNG

**BursR_Tick**  GNGLVVVVLSLGRSKMDVPRFLVCNLALADFFMGVYLGFLAVVDASTLGEFRVYGIGWQMSAGCQTAGFLGVLSSELSVYTLSVITMERNYAITHAMHLNRLSLKHAGYIMSCGWLFALVMAVLPLVGVSDYRKFAVCLPFETEDLASLAYVVSLILVNG

**BursR_SeaAnemone**  GNVTVIIVILVSKTKMDVSRFLIVNLAVADMCMGLYLGLLAIVDASTIGDFLHHGVEWQLSTGCKTAGFLALLSSEASVFTLTVITIERFIAIRHALHIHKMSLRKTTIVMVIGWGLALIIATLPLAKVSDYTKVSVCLPFEIGEIESLTFVTFTMVLNC

**BursR_Coral**  GNATVIFVIMARSSRIDVSRFLICNLAVADLCMGIYLGLLAIVDASTRGNFRSYGVKWQLSSGCKTAGFLAVLSSETSVFTLTVITVERYIAITHALDITKMSLKKTAVVMFIGWCFALIAATLPLFDVSDYTKFSVCLPFETGDTKSLAYVTSVLTLNG

**BursRa_Placozoan**  -NTLVLMVILNAEGHLTVTKFLICNLAFADFCMGIYLALMAITDLTTANQYANYAINWQTGGGCATAGFIAIFSAELSTMTLTVITIERYLAIVHAMYYEQLKLFGACIAMSICWILSIVLALLPIVGVNSYSLICICLPADFTSSMSVGYILILVGINA

**BursRb_Placozoan**  GNILVLIVIYGSPLKFTITKFLICNLAIADLCMGFYLGTMATIDAIFADNYANFAIAWQLSAGCKLAGFIAVFSTELSVYTLLMITIERYMVVTNAMYYKHLKLRGACIAMLIGWIFGLTLATLPLLHVNSYSKTCICLPSDIDTLVGKLYMAVILTLNG

**CKHR_CombJelly**  --------------RFTPTRFLIVNLSFSDLLTGIYLMMLAAVDAKTYGFYEDYAVDWQNGPGCQIAGFMVTFSSELSVFTLTMISVERMVTIIHYSNPYHLKTRHIALAMLLGWLISATAGAFPLLGFSDYSLVAICLPFQNSDGGDLALILSLMVINT

**CKHRa_Sponge1**  -------FFRHKQKHLQVVNFFYLNLATADFLMGVYLFTIGTEDLNTPGAFFTQSAAWQSSPGCNFAGFCAMTSISMSVFTLVLITLERVHSIVQVFEQRKMSMKLAALLMAAGWVFAVVMGALPIAHVSDYGKTAVCLPFDSSSAVSAGYVYFVLIATG

**CKHRa_Sponge2**  ---------LKNHKKPHLMYFLYINLAMADLFMGIYLLTIAVVDLDTIGDYSRHAIEWQTSAGCRFAGFCAIFSSLLSIYTLLVITVERVYTIKFALQHKRFHKQTVTVSILVGWVLTITLCILPMVGLSSYERVGICLPFEARETADQAYIVLILVLTG

**CKHRb_Sponge1**  GNGSVLFVMLASKDKIKVPQFFISNLAFADFCLGIYLAFLACVDARTYGQFYQSALEWQRGPGCSTAGFIAVFSSMLSCSMLVAVTLERVYTICHSPKTG-ISMSTAIVVALFCWLISFAFATLPLVGINSYSRVAVCLPFVTAELRDKIYIGVLLTTTM

**CKHRb_Sponge2**  -NLAVLFVMIVSKEKLEVPHFFICNLAFADFLLGVYLAFLGAVDIRTRGGFYKSALTWQTGPGCQTAGFIAIFSAELSVFILTFLTLERLHTIAYSFKSGRLRLRNATIIVFICWILAGILAALPLFDVNTYSEVAVCLPFRLSNIRDKLFIALILTVNL

**CKHR_Sponge3**  NTI-VFIVIVAPKYETRTSSFFIMFLSLADFLMGVYLLILAVVDLTTTSEYSEHALDWQVSSGCNAAGFFAVFSSMLAVFTLTIITIDRFISMRFTMGGKRLNLQKARVSVILGVIFALVVAVLPLTGVSSYNEVGICLPFSVESHKDLGFITFLLVIRL

**RXFP1_Human**  GNIFVICMRPYIRSENKLYAMSIISLCCADCLMGIYLFVIGGFDLKFRGEYNKHAQLWMESTHCQLVGSLAILSTEVSVLLLTFLTLEKYICIVYPFRCVRPGKCRTITVLILIWITGFIVAFIPLSNKEFYGTNGVCFPLHSEDTEAQIYSVAILGINL

**RXFP1_Chicken**  GNIFVICMRPYIRSENKLHAISIMSLCCADCLMGIYLFVIGAFDLKYRGEYNKHAQLWMDSIHCQLVGSLAILSTEVSVLLLTYLTLEKYICIVYPFRCLKPRKCRTISILVLIWVIGFAVAFIPLSNKEFYGTNGVCFPLHSEQSESQIYSVVILGVNL

**RXFP1_Lizard**  GNIFVICMRLYMRSENKLHALSIMSLCCADCLMGIYLFVIAVFDLKYRGEYNKHAQLWMDSIHCQLVGSLAILSTEVSVLLLTYLTLEKYICIVYPFRFLRPGKYGTISVLVLIWLIGFVVAFIPLSNKDFYGTNGVCFPLHSEKSEGQIYSVIILGVNL

**RXFP1_Frog**  GNIFVICTRPYIRSENKLHAMSIISLCCADCLMGVYLFVIGYFDLKYRGEYNENAQAWMDSTQCRLVGSLAILSTEVSVLLLTYLTLEKYICIVYPFRCLKPGKCRTITTLILIWIIGFVIAFIPLSNQTFYGTNGVCFPLHSEQPEAQIYSVIILGVNL

**RXFP1_Coelacanth**  GNIFVICMRPYIRSENRLHTMSIMSLCCADCLMGIYLFVIGAFDLKYRGEYNQHAQLWMDSMQCQLIGSLAMLSTEVSVLILTYLTLEKYICIVYPFQCLTPGKRRTVATLIAIWIVGFVIAFIPLVSKDFYGTNGVCFPLHSEQTEAQIYSVIILGLNL

**RXFP1_Fugu**  -NIFVICMRAYIRSENKLHAMCIISLCCADGLMGVYLFMIGAYDLKYRGEYNRHAQAWMDSIQCQVIGSLAMLSTEVSVLLLTYLTLEKYICIVYPFQYLTPGWQRTVSILLGIWVFGFVVAFLPLACKEPYGTNGVCFPLHSEQPEAYVYSIVILGLNL

**RXFP1_Medaka**  GNIFVICMRSYIRSENKLHAMCIISLCCADGLMGVYLFMIGSYDLKFRGEYNRHAQSWMDSTQCQIIGALAMLSTEVSVLLLTYLTLEKYICIVYPFQYLTPGRRRTVTILTSIWVFGLIVAFLPLACKGLYGTNGVCFPLHSEQPEAHVYSIVILGLNL

**RXFP1_Zebrafish**  GNIFVICMRSYIRSENKLHAMCIISLCCADGLMGVYLFMIGAYDLKFRGEYNRHAQAGMDSEACQVIGSLAMLSTEVSVLLLTYLTLEKYICIVYPFRYLTLGRRRTVTILVVIWVLGFIIAFLPLLFKGVYGTNGVCFPLHSEQPEAQIYSIVILGLNL

**RXFP2_Human**  GNLFVIGMRSFIKAENTTHAMSIKILCCADCLMGVYLFFVGIFDIKYRGQYQKYALLWMESVQCRLMGFLAMLSTEVSVLLLTYLTLEKFLVIVFPFSNIRPGKRQTSVILICIWMAGFLIAVIPFWNKDYYGKNGVCFPLYYDQTESKGYSLGILGVNL

**RXFP2_Chicken**  GNLCVICMRSCVVTESSAHTMAIKSLCCADGLMGIYLFVIGAFDLKYSGEYNRHAQSWMASVPCQLVGSLAMLSSEVSVLLLTYMTLEKYLSIVFPFSYRRAGRKQTASVLAAIWLLGLSLSIVPLCCKESYGRNGVCFPLQSELGEARGFSTTILGLNL

**RXFP2_Lizard**  GNLFVIFTRSFIVTEHSKHTMAIKSLCCADCLMGAYLFFLGAFDLKFSGEYNRHAQAWMSSPSCHLVGSLAVLSAEVSVLLLTYMTLEKYLCIAFPFGRYGASERRTRLSLALLWLLGLSLTLLPFSCKEAYGSNGVCFPLQSHPNEARGYSTGILGLNL

**RXFP2a_Frog**  GNIFVIGMRSCIQSENKTHTMSIKVLCCADCLMGIYLFFIGVFDVKYRGQYKKYALLWMESLQCRSLGFLAMLSTEVSVLLLTFLTLEKYLAIVFPFSNIRPGKRQTLIILISLWAVGFIIAIVPFWNEDFYGKNGVCFPLYPDQTEGQGYSLGVLGVNL

**RXFP2b_Frog**  GNLFVICMRSFIVTENCQHTMSIKSLCCADCLMGFYLFCLGAFDIKFQGEYNKHAQAWMESWECKLVGSLAMLSSEVSVMMLTYMTLEKYLCIVFPFSHFRAGRGQTLCTLTAIWGLGFFITVIPFFSYDTYGRNGVCFPLQSDSTEARGYSVSILGLNL

**RXFP2a_Coelacanth** GNVFVICMRSFIRAENKLHAMCIKILCCADCLMGVYLFFVGVFDVKYRGEYNKYAHSWMESLQCRIIGFLAMLSTEVSVLLLTYLTVEKYFVIVFPFSNIRPGKCQTMIILISIWIIGFVIAVIPIWKEDLYGRNGVCFPLHSDQTEAKGYSIGILGLNL

**RXFP2b_Coelacanth** GNIFVICLRSCIVSENRQHTMSIKSLCCADCLMGVYLFFIGAFDIKYYGEYNRHAQEWMESVQCQFIGSLAMLSTEVSVLLLTYMTLEKYLCIVFPFSHYRAGKKQTLSTLVSIWVLGFIIAVIPFWDKDSYGKNGVCFPLNNDLAEARGYSTVILGINL

**RXFP2_Fugu**  GNLLVIGMRSLIRAENNQHAACIKVLCCADCLMGVYLFFLGVSDVKFRGEYNRNALLWMESVECRTIGFLAMLSSEVSVLLLTYLTLEKFLVIVFPFSNLRPSKPLTGVVLASIWLLGVVIAAVPLMNEDVYGRNGVCFPLHSDRQEAKGYSTGILGLNL

**RXFP2_Medaka**  GNLLVIGMRSLIRAENNLHAVCIKVLCCADCLMGVYLFFVGVFDVKFRGQYNRNALQWMESVECRTIGFLAMLSSEVSVLLLTYLTLEKFLVIVFPFSNLRPGKLQTVVILSSIWLMGFIIAAVPLMNEEIYGRNGVCFPLHSDRQEAKGYSTGILGLNL

**RXFP2a_Zebrafish**  GNLFVIGMRSFIRAENNLHAACIKVLCFADCLMGVYLFFLGIFDVKFRGEYNRNALIWMDSVECRTIGFLAMLSSEVSVLLLTYLTLEKFLVIVFPFSHLRPAKLQTVLILAFIWFLGFVIAAVPLLNEDLYGRNGVCFPLHSDRLEAKGYSTGILGLNL

**RXFP2b_Zebrafish**  GNLFVIGMRTVLRAENNLHAFCIKVLCCADCLMGVYLFFVGIFDVKFRGEYNKNAKVWMESLECRTIGFLAMLSSEVSVMLLTYLTLEKFLVIVFPFSHLRPSKCQTFTVLVSIWLLGISIAAVPLLNEDTYGLNGVCFPLHSERLEAKGYSTGILGLNL

**RXFP2c_Zebrafish**  GNIFVICLRSCIASENQHHTMAIKSLCCADCLMGVYLLFIGAFDIKYCGEYNRHAQIWMESLSCQLIGSLAMLSTEVSVMMLTYMTLEKYLCIVFPFQHYRAGRKQTLCSLTFIWLLGFIIAVIPFWDKQTYGRNGVCFPLHSDQTEARCYSTAILGLNL

**RXFPa_Amphioxus**  GNTGVIIGRTAIKQENKVHSFFIQNLCASDLIMGVYLLIIGTKDVMLRGVYNQHAEEWKTGYGCKLSGFLAMLSAEVSVLLLTYMSVERFLCVVFPYRDNRPDRWQAGMTILLIWLGGFLLALVPLMVPEYYGSNGVCFPLHLHEPYGWEYSAFVIGINF

**RXFPb_Amphioxus**  GNTAVMIGRTVMKQENKVHSLFIKNLCASDFIMGVYLLVIATKDMTFRGVYHRHTHAWTNSLGCQITGFLAMLSAEVSVLLLTYMSVERFLCVVFPYRDNRPNLRQAAVAICLIWFFGLLLSAAPLFIPYYYGSNGVCFPLHLHEPHGWEYSAFILGVNF

**RXFPa_Urchin**  GNLGVLVSRAFMKAENKVHTVVVINLCTADFLMGLYLLIIGTQDVKYRDNYNQYALEWTNGITCKVSGLLAIISSEVSVLTLMFISLERYFIIVYPYSFQRIKAKRAILILAVIWFIGTLLAVFPMIPVAYYGSNGVCFPLHLHDPRGWEYSAFLLGVNT

**RXFPb_Urchin**  GNLGVLVSRAFMKAENKVHTVVVINLCTADFLMGIYLLIIGTHDVKYRDNYNQHALEWTNGITCKVSGLLAMISSEVSVLTLMFISLERYFIIVYPYSFQRIKTRRAIMVLAFIWFFGTLLAVVPIIPVDYYGSNGVCFPLHLHEPRGWEYSVVVLGINS

**RXFP_Oyster**  GNLLVLLGRSVLREDNQVHSFYIKNLSFADMLMGLYLIIIGFHDQLFRGNYLVEDEEWRSSATCDVCGILSTFSNEASVLTLTLITLDRYISITLPLFRRRKSFKFALMNVCIIWIISLLLSVLPVTIPDYYKDNAVCVPFQLHRPRGWEYSTFLLGLNL

**RXFP_Limpet**  GNIIVLLGRFLLKEDNQIHSFFIKNLSFADMIMGVYLLMIGTRDKMYRGNYVLHDEEWRNSWTCDLSGIISTVSSEVSVLTLTVITLDRYICIMYPLSLRKRGLKVAYCVMGFTWLMCILFAVIPVIGIPYYRNNGVCIPLHLHDPRGWEYSSFLIGMNL

**RXFP_FruitFly**  GNVLVLWGRFIYRDENVAVTMVIRNLALADMLMGFYLVTIGVQDYRYRNEYYKVVLDWITSWQCTLIGTLAVSSSEVSMLILAFMSLERFLLIADPFRGHSIGNRVMWLALICIWITGVGLAVAPVLLWRTYGYSGTCFPLHIHEAFGWLYSAFVLGVNL

**RXFP_Daphnia**  GNALVLGGRNLAKTENRILAMFVKNLAAADMCIGIYLLCLGERDISFRGQYNQHAHQWMMSWQCTVIRVLAVSTSEVSLLLLTFMSVERFVSISHPFGERTLNFRAAFISTALIWTVGLALSLIPVLYWSEHGSNGLCFPLHIHEPSGWQYSAFLIGING

**RXFP_Mite**  -NSVVFTWRVISKKEDRVLSLFIKNLSIADFLMGVYLLIVGILDVAFRDEYNRHARQWMTSWRCTAVGLLAMISCEVSVLILSLITIERYRCIKTNVRVV--TVTAARWCVVGVWCTGFLLALYPVFHWPQYSSNGLCFPLHIDDPFGWQYSALVLGINL

**RXFP_Tick**  GNSVVFAWRFLAKKEDRVLSLFIKNLSMADLLMGIYLVTVGSLDVAFRDEYNKHAHQWMSSWFCTLCGLVAMVSCEVSVLILSLITIERYCCIKTNVRAV--TVNAARYFLAVVWLAGLVLALFPVLRWPSYSSNGLCFPLHIDDPFGWEYSAFVLGINF

**RXFPa_SeaAnemone**  GNVFVIFWRVTDGQSNQTHSLLLTNLAVSDLLMGVYLMIIAITDAKWQGEYFKHDVTWRAGIGCQFAGIISMLSSEVSVFILALITADRLICIVFPFKFRRLTRKRAMILCVIIWTLGAIASLLPVTGISYYGRSGVCLPLQLSKSRGWEYSVAFIGLNS

**RXFPb_SeaAnemone**  GNIFVVAYRLVVREDNRVHSLLLTNLAISDFMMGLYLLIIAFKDVQWQGEYFKHDLSWRVSGLCQFAGALSMISSEVSVLMLTIITLDRFICIVFPMRFGRLGLKKAIAICILLWTFGTLISVVPILGIEYYGRSSVCLPLQLSSDRGWEYSVSFIGLNF

**RXFPa_Coral**  GNLIVILMRVVTKEDNNVQSVLLTNLAISDLLMGIYLLIIAIKDVQWQGEYFLHDFRWRSGVPCALTGVLSMISSEVSVLMLTVLTTDRLICIVFPFKVRGMNRSVAYAVVGGVWVLGTMLAVIPILWFEYYGKSAVCLPLQLSEDKGWEYAVGIIGLNF

**RXFPb_Coral**  GNLFAILLRVVVKEDNKVHSFLLTNLALSDLLMGIYLLIIAIKDVQWQGEYFLHDFKWRSGVLCALTGVLSMVSSEVSVLMLTVITTDRLICVVFPFKVRRMNRSVALAVVGGVWVFGAMLAVIPILGLEYYGKSAVCLPLQLSSERGWEYAAGIIGLNF

**GRL101_Urchin**  GNLVVIMWRVNSKRDNKVHSFLITNLAVGDMLMGIYLLIIAGVDAYYRGDYIVHDKTWRNSGLCKFAGFLSTFSSELSVFSLTIITLHRLSSIVFPFRIKDMEFTRAVWVMCVSWGLVAFLAALPLCGVAYYGRSGVCLALHITPDKGWEYSVFILGLNF

**GRL101_Oyster**  GNLLVFIWRVRDDRNGKVHSFLITNLAVGDFFMGVYLLIIAVVDSYYRGEYIIFDRSWRESELCKFAGFISTFSSELSVFTLTVITLDRLICIIFPLKLKRLGIKQASVVMPCIWIVVIVLSGIPLFGLDYYGRSGVCLAFHITPDKGWEYSVFVLVLNF

**GRL101_Limpet**  GNLLVIGWRARDLRGGKVHSFLITNLAIGDFFMGIYLLIVAVVDSYYRGVYIVHDKAWRNSDLCRFAGFLSTLSSELSVFTLTVITLDRLICIIFPLKMRRLSLKESFLVMLGVWLVVLCLSVLPLIGLDYYGRSGVCLAFHITPDHGWEYSVAILVVNF

**GRL101_Louse**  GNLLVIGWRMNYKHKNKVHSFLITNLAVGDFLMGFYLLIIASVDAHYRGVYSVHDEEWRSSKLCSLAGFLSTLSSELSVFTLILITFESFLVIMFPFKVTRLQMSEIRWVMLGVWIAAVCLSGLPLLYKDYYGRTAVCLALCITIDKGWKYSAFIIFLNF

**GRL101_SeaAnemone** GNGFVLIWRLKTKSESRVHSLLLLNLALSDFMMGVYMVVIGSVDKYYRGKYFIYNEEWKRSHLCQFCGFLSTVSCEASVFILTTMTVDRYVAIVHPLRNLCLKISGAYKALISIWLLAFILAFLPLTGIKYYGRSGVCLPLHLTAEKGWEYSVFVLVLNF

**GRL101_Coral**  GNAFVLLWRLKTKSDNRVHALLLLNLAIADFFMGIYLALIGSVDAFYRGRYFIYNDRWKHSPLCQFSGFVSTLSSEASVMILTVMTMDRYVTIVHPFKHFGLSIRGAHVILIITWIAAFVLAGVPLTGIPYYARSGVCLPLHLTADKGWEYSVFLLALNF

**GRL101a_Placozoan** --------WRLKRNINTVNSFLVGQLAIADFLMGVYLIIIASADAYFRDNYSENERNWIRSITCQIAGFLATLSSEVSVYILACITTDRLICIVYPHSKSKITLRLAKIIAACGWILIAIIFAIPLFDIQFYSKSSVCLPLHIATDSSWLYSIVMTFTNM

**GRL101b_Placozoan** ---------------ESVNVLFVDALAIADLMMAIYLIIIGSTDIYYRKKYSHYDDYWRKSPLCHFAGFLATLSCQMSVYILTAITVDRLICIISPYSPYRINLSTARKAIFWGWLIVIVTIGIPLLGIPYYGRSSVCLPFHFSNVDGWQYSISVTVVNL

**OpsB_Human**  LNAMVLVATLRYKKLRQPLNYILVNVSFGGFLLCIFSVFPVFVAS--------CNGYFVFGHVCALEGFLGTVAGLVTGWSLAFLAFERYIVICKPFGNFRFSSKHALTVVLATWTIGIGVSIPPFFGWSREGLQCSCGPDWYTKYRSESYTWFLIFCFI

**Ops1_FruitFly**  GNGVVIYIFATTKSLRTPANLLVINLAISDFGIMITNTPMMGINL--------YFETWVLGMMCDIYAGLGSAFGCSSIWSMCMISLDRYQVIVKGMAGRPMTIPLALGKIAYIWFMSSIWCLAPAFGWSREGNLTSCGIDYLEDWNPRSYLIFYIFVYY

**OPS3_FruitFly**  GNGLVIWVFSAAKSLRTPSNILVINLAFCDFMMMVKT-PIFIYNS--------FHQGYALGLGCQIFGIIGSYTGIAAGATNAFIAYDRFNVITRPMEGK-MTHGKAIAMIIFIYMYATPWVVACYETWGREGYLTSCTFDYLTNFDTRLFVACIFFSFV

**ACM1_Human**  GNLLVLISFKVNTELKTVNNYFLLSLACADLIIGTFSMNLYTTYL--------LMGHWALGLACDLWLALDYVASNASVMNLLLISFDRYFSVTRPLSYRKRTPRRAALMIGLAWLVSFVLWAPAILFWQYER----IQFL-----SQPIITFGTMAAFY

**n5HT7R_Human**  GNCLVVISVCFVKKLRQPSNYLIVSLALADLSVAVAVMPFVSVTDL-------IGGKWIFGFFCNVFIAMDVMCCTASIMTLCVISIDRYLGITRPLTYPRQNGKCMAKMILSVWLLSASITLPPLFGWAQ--DDKVCLIS-----QDFGYTIYSAVAFY

**n5HT1R_FruitFly**  GNVLVCIAVCMVRKLRRPCNYLLVSLALSDLCVALLVMPMALLYE--------VLEKWNFGLLCDIWVSFDVLCCTASILNLCAISVDRYLAITKPLEYGKRTPRRMMLCVGIVWLAAACISLPPLLILGN-EGQPICTVC-----QNFAYQIYALGSFY

**n5HT1A_Human**  GNACVVAAIALERSLQNVANYLIGSLAVTDLMVSVLVLPMAALYQ--------VLNKWTLGVTCDLFIALDVLCCTSSILHLCAIALDRYWAITDPIDYVKRTPRRAAALISLTWLIGFLISIPPMLGWRTRSDPDACTIS-----KDHGYTIYSFGAFY

**n5HT1B_Human**  SNAFVIATVYRTRKLHTPANYLIASLAVTDLLVSILVMPISTMYT--------VTGRWTLGVVCDFWLSSDITCCTASILHLCVIALDRYWAITDAVEYSKRTPKRAAVMIALVWVFSISISLPPFF-WRQEEEVSECVVN-----TDILYTVYSVGAFY

**DRD1_Human**  GNTLVCAAVIRFRHLRKVTNFFVISLAVSDLLVAVLVMPWKAVAE--------IAGFWPFG-FCNIWVAFDIMCSTASILNLCVISVDRYWAISSPFRYEKMTPKAAFILISVAWTLSVLISFIPVQSWHKTSTIDNCDSS-----LSRTYAISSVISFY

**ADRB1_Human**  GNVLVIVAIAKTPRLQTLTNLFIMSLASADLVMGLLVVPFGATIV--------VWGRWEYGFFCELWTSVDVLCVTASIETLCVIALDRYLAITSPFRYQLLTRARARGLVCTVWAISALVSFLPILHWWRDEDPKCCDFV-----TNRAYAIASVVSFY

**ADRA1D_Human**  GNLLVILSVACNRHLQTVTNYFIVNLAVADLLLSATVLPFSATME--------VLGFWAFGAFCDVWAAVDVLCCTASILSLCTISVDRYVGVRHSLKYPIMTERKAAAILALLWVVALVVSVGPLLGWKE-PDERFCGIT-----EEAGYAVFSVCSFY

170 180 190 200 210 220 230 240

....|....|....|....|....|....|....|....|....|....|....|....|....|....|....|....|....|.

**FHSR_Human**  LAFVVICGCYIHIYLTVRNPNIVSSSSDTRIAKRMAMLIFTDFLCMAPISFFAISASLKVPITVSKAKILLVLFHPINSCANPFLY

**FSHR_Chicken**  LAFVIICICYICIYFTVRNPNVISSNSDTKIAKRMAILIFTDFLCMAPISFFAISASLRVPITVSKSKILLVLFYPINSCANPFLY

**FSHR_Frog**  LAFVVICTCYIGIYLTVRNPDVISSNSDTKIAKRMAILIFTDFLCMAPISFFAISASLKIPITVSKSKILLVLFYPINSFANPFLY

**FSHR_Lizard**  LAFIVICTCYISIYFTVRNPNVFSSNNDTKIAKRMAILIFTDFLCMAPISFFAISASLKVPITVSNSKILLVLFYPINSCANPFLY

**FSHR_Medaka**  LAFFCVCGCYLSIYLTYRKPSSAPAHADTRVAQRMAILIFTDFLGMAPVSFFAISAALKLPITVSDSKLLLVLFYPINSYSNPFLY

**FSHR_Zebrafish**  AAFLVVCVCYTLIYLTVRNPAFVPANADMRIAKRMAVLIFTDFLCMAPISFFAISAAFKLPITASHAKVLLVLFYPINSCSNPFLY

**LHCGR_Human**  VAFFIICACYIKIYFAVRNPELMATNKDTKIAKKMAILIFTDFTCMAPISFFAISAAFKVPITVTNSKVLLVLFYPINSCANPFLY

**LHCGR_Chicken**  IAFLVICACYIKIYVAVQNPELVAANKDTKIAKRMAILIFTDFTCMAPISFFAISAAIKVPITVTNSKILLVLFYPVNSCANPFLY

**LHCGR_Frog**  IAFIIICACYIKIYIAVQNPELAPTNKDTKLAKKMAVLIFTDFTCMAPISFFAISAAFKVPITVTNSKILLVLFYPVNSCANPFLY

**LHCGR_Coelacanth**  IAFLVVCICYVKIYLSVQNPDFAAKNKDTKIAKRMAILIFTDFTCMAPISFFAISAAFKVPITVTNSKILLVLFYPINSCANPFLY

**LHCGR_Zebrafish**  GAFLVICGCYVCIYSAVRNPEFPGRAADAKIAKRMAVLIFTDFLCMAPISFFAISAAFKVPITVTNSKILLVLFYPINSCANPFLY

**TSHR_Human**  VAFVIVCCCYVKIYITVRNPQYNPGDKDTKIAKRMAVLIFTDFICMAPISFYALSAILNKPITVSNSKILLVLFYPLNSCANPFLY

**TSHR_Chicken**  IAFVIICACYIKIYITVRNPQYKSGDKDTKIAKRMAVLIFTDFLCMAPISFHALSAIMNKPITVTNSKILLVLFYPLNSCANPFLY

**TSHR_Lizard**  IAFIVICACYIKIYITVRNPQYKSGDKDTIIAKRMAVLIFTDFLCMAPISFYALSAIMNKPITVSNSKILLVLFYPLNSCANPFLY

**TSHR_Frog**  IAFIIICACYIKIYITVRNPQYKSGDKDTKIAKRMAILIFTDFICMAPISFYALSAIMNKPITVSNSKILLVLFYPLNSCANPFLY

**TSHR_Coelacanth**  IAFIIICACYIKIYITVRNPHYKSGNKDTKIAKRMAILIFTDFICMAPISFYALSAILNKPITVTNSKILLVLFYPLNSCANPFLY

**TSHR_Medaka**  MAFFVICACYIKIYCTVRNPHYHSGSKDTNIAKRMAILIFTDFLCMAPISFYAMSAVLDRPITVSKSKILLVLFYPLNSCANPFLY

**TSHR_Fugu**  LAFFIICACYFRIYCAVHNPHYHSGSKDTNIAKRMAVLIFTDFLCMAPISFYALSAVLDRPITVSNSKILLVLFYPLNSCANPFLY

**TSHR_Zebrafish**  LAFIVICVCYIKIYCAVHNPSHQSSNKDTNIAKRMAVLIFTDFLCMAPISLYAMTAVLDHPITVSNSKILLVLFYPLNSCANPFLY

**GPHRa_Lamprey**  LAFLVICVCYAHIYSTVRNPSYNLGSHDAKIAKRMAVLIFTDFTCMAPISFFAILAAAKLPITVSHTKILLVLFYPLNACANPLLY

**GPHRb_Lamprey**  LAFVIICACYVHIYITVRNPNYISGNQDTKIARRMAILIFTDFICMAPISFFAISAAFKLPITVTNTKILVVVFYPLNSCANPFLY

**GPHR_Amphioxus**  LAFGLICGCYIGMYLAVRHTDGEAKKNDAKVAKRMAILVFTDFACWFPIALFGITAAFGHPIDVTNSKILLVIFYPINSCANPFLY

**GPHR_AcornWorm**  LAFLLVVGCYVKMYHTVRSPESLAHKKDATVAKRMGILVFTDFACVVPIMVFAFTAALAKPIGVEEAKILLVIFYPINSCTNPFLY

**GPHR_Urchin**  IAFAVICACYVKMYLTVRNPHTVMQRKDSKVAKRMAVLVFTDFACWAPMALFGLSGAFGHHLTTNQSKIFIVLFYPINSCANPFLY

**GPHR_Oyster**  LAFIVICMCYINMYCRVRGNDSLSQSSDTRIAKRMATLIFTNFICWAPIAFFGITAAAGWPIDVSNSKILLVFFYPLNSCANPYLY

**GPHR_Limpet**  VAFIVICCCYVNIYFNVQQNNTTARTNDATIAKRMAILVFTNFVCWAPIAFFGLTASAGFPIDITNSKILLVFFYPLNSCANPFLY

**GPHR_Aplysia**  VAFVAICGCYISMFLQVRASESMARSNDATIAKRMAILVLTNFICWAPIAFFGLTASSGIPIDITNSKILLVFFYPFNSCANPFLY

**GPHR_Capitella**  LAFCLICFCYVDMFRQVRGDSSTASHNDLTIAKKMSILVFTDFLCLFPIAFFGLTAAAGNSIDVTESKILLVFFFPLNSCANPFLY

**GPHR_FruitFly**  VAFSIIAVCYAQIYLSLGRETRQNSPGELSVAKKMALLVFTNFACWSPIAFFGLTALAGYPINVTKSKILLVFFYPLNSCADPYLY

**GPHR_Silkworm**  LAWVTIVVCYVQIYRSLGGGGGAAAAAERRIANKMALLIGTDLLCWAPVAFFGVTALAGVPVDVSHGKVLLVFFYPLNACANPFLY

**GPHR_RedFBeetle** IAFALIAFCYAQIYLSLGQET----RHEMAIAKKFALLVFTDFATWAPISFFSVTALAGYPIGVTKSKILLVFFYPINSCANPYLY

**GPHR_Louse**  LAFWLICTCYGKMYCSIKKGQLARSHSDMTVAKRMALLVFTDFACWAPITFFGLTALSGYPIDVSKTKILLVFFYPLNSCANPYLY

**GPHR_Daphnia**  IAFLFICLCYSHMYRSIRGNHQLASHSDTTVAKRMALLVFTNFACWTPIAFFGLTAVAGYPISVTNSKILLVFFYPLNACTNPYLY

**GPHR_Mite**  LAFILICACYARMYSSIAGQQMANYYGDTTVAKRMALLVFTDFACWAPIAFFGLTAVAGYPIDMTKSKILLVFFYPLNSCANPFLY

**GPHR_SeaAnemone**  AAFAFVLYLYMRMLMTVISGG-APKRDDSKVAKRMALLVLTDFVCWAPIAFFGILAAFGTPIDVTASKTLLVFFFPINSLCNPFLY

**GPHR_Coral**  GTFLVVAGLYAKMFQVVVGPG-APQRNDAKVAKRMALLVFTDFVCWTPIAFFGLLAAFGIPIGVEESKFLLVFFFPLNSLCNPFLY

**BursR_Urchin**  LAFVIIMLCYTSIYCSIQGSHA-WNSNDSRVARRMSLLVFTDFICWAPFAIFALASAFGKDIPLAGSKVLIVFVLPVNSCANPFLY

**BursR_Oyster**  VSFVVIVICYIKMYCSILGSNA-WNSKDFRIAKRMAILVFTDFLCWAPIIFFSITAAFGKNVGLNEAKVLTIFVLPLNSCANPFLY

**BursR_Limpet**  LCFFTILSCYLIMYLSIRQSQA-WNSGDTRVAKRMALLVFTDFICWAPIALLSLASAFGKNIHLNEAKVLTIFVLPLNSCANPFLY

**BursR_Capitella**  IAFFIIVACYMKMYCSIRGSQA-WHSNDTRIAKRMALLVFTDFACWAPITFFSLTAAFGKEISLNDAKVFTIFVLPLNSCANPFLY

**BursR_FruitFly**  CAFLTLMGCYLKMYWAIRGSQA-WNTNDSRIAKRMALLVFTDFLCWSPIAFFSITAIFGLQISLEQAKIFTVFVLPLNSCCNPFLY

**BursR_RedFBeetle** VAFLILMGCYLKMYCAIRGSQA-WNSNDSRIAKRMALLVFTDFLCWSPIAFFSLTAAFGLQISLEQAKVFTVFVLPLNSCCNPFLY

**BursR_Louse**  VAFFILMGCYLKMYCAIRGSQA-WNSNDSRIAKRMALLVFTDFLCWSPIAFFSLTAAFGVQVSLEQAKVFTVFVLPLNSCCNPFLY

**BursR_Daphnia**  VAFLILMGCYLKMYCAIRGSQA-WNSNDSRIAKRMALLVFTDFLCWAPIAFFSLTAASGLQVSLEEAKIFTVFILPLNSCCNPFLY

**BursR_Mite**  VAFLILMGCYLKMYCAIRGSQA-WNSNDSRIAKRMALLVFTDFLCWAPIAFFSLTAVSGMHINLEEAKVFTIFVLPLNSCANPFLY

**BursR_Tick**  VAFLILMGCYLKMYCAIRGSHA-WNSNDSRIAKRMALLVFTDFLCWAPIAFFSLTAVAGLQVSLEEAKVFTIFVLPLNSCANPFLY

**BursR_SeaAnemone**  AAFLVIFGCYVGIYLQVRGSNA-WNTNDTQVALRMSLLVVTDLMCWAPIAFLALTATFGTTVSLNEAKVFTVFCFPLNSCANPFLY

**BursR_Coral**  TAFLVILTCYIKIYCAIRGSSA-WNTNDFRTAQRMALLVFTDFTCWAPITFLSLAAAFGGDVSLKEAKIFTVFVFPLNSCANPFLY

**BursRa_Placozoan**  LAFILIVYSYCVMYYTVHSTHSATQEADRRIARRMALLIFTNFACWTPIAFFGFGAALKLNITVSSAKFLLVFIFPINATANPFLY

**BursRb_Placozoan**  MATILVMGCYLHIFYVVHYSNSTANRTDLIIARRMLILIFVDFLCWAPIAVFGLSAVFGLKISVSKAKFLMVFIFPLNSCMNPFLY

**CKHR_CombJelly**  IAVMVIIYCYLTIFCNVRHLQKPLDNADFRETLRMALIILTDVLCWLPIVVCGVSAAVGKPITTSNAKILVVFFLPLNACANPFLY

**CKHRa_Sponge1**  VASLVITTSYIVIGCNVFYNRKASKMNECYVAIRMSVIVLSNFACWFPIAVVGLGSATGQKIGLDDAKVFMVVVFPLNASLNPIIY

**CKHRa_Sponge2**  IASFAIMFCYVLLFYLVVCNNRLSGREELKLALRMSLLVMTDFACWAPIALFGLTAVFQKPINVTDSKILMVFVFPLNSCLNPILY

**CKHRb_Sponge1**  VAFLIILVSYIVILCIVCRSPAQNRKETVKLLRKMAPLVITNFLCWFPIVVIGYSALADKPIGVSQAKWLVVLVYPFNACANPFWY

**CKHRb_Sponge2**  TAFFIILSSYLHILRLFCRSRAGNKREKIVISFKMGMLVLTNLICWLPLAVVGYAAIVDQHINFTVAKFFIILIFPINACLNPFIY

**CKHR_Sponge3**  VSLLIIGLAYYLIYKKYTESQSVWNPKEKKVAIRMTFLVVSNCICWVPISVLGLLVLYGPRISLFAAKILVIFFFPFNAALDPFLY

**RXFP1_Human**  AAFIIIVFSYGSMFYSVHQSAINQVKKEMILAKRFFFIVFTDALCWIPIFVVKFLSLLQVEIPGTITSWVVIFILPINSALNPILY

**RXFP1_Chicken**  AAFLIIVFSYGSMFYSVHQTAINHIKKEMILAKRFFFIVFTDALCWIPIFILKLLSLLQVEIPGTITSWVVIFILPINSALNPLLY

**RXFP1_Lizard**  VAFIIIVFSYGSMFYSIHQTAINHMKKEITLAKRFFFIVFTDALCWIPIFILKLLSLLQVEIPGSITSWVVIFILPINSALNPLLY

**RXFP1_Frog**  AAFIIIVFSYSSMFYSIHRTAINHIKKEMTLAKRFFFIVFTDALCWIPIFILKLLSLLQVEIPGSISSWVVIFILPINSALNPILY

**RXFP1_Coelacanth**  LAFIIIVFSYASMFYSILQTGAAQVKKEMTIAKRFFFIVFTDALCWIPIFVLKVLSLLQVEIPGSISSWVVIFVLPINSALNPILY

**RXFP1_Fugu**  VAFLIIVFSYASMFYNIQRTGTNHIKKEVTIAKRFFSIVITDSLCWIPIFILKILSLLHVEIPGTISSWVVIFILPINSALNPILY

**RXFP1_Medaka**  VAFLIIVFSYASMFYNIQRTGTNHIKKEVTIAKRFFSIVITDSLCWIPIFVLKILSLLQVEIPGTISSWVVIFILPINSALNPILY

**RXFP1_Zebrafish**  VAFLIIVLSYGSMFYNIQRTGTNHIKKELTIAKRFFSIVITDSLCWIPIFILKTLSLMEVEIPGTISSWVVIFILPINSALNPILY

**RXFP2_Human**  LAFLIIVFSYITMFCSIQKTALNCFGREVAVANRFFFIVFSDAICWIPVFVVKILSLFRVEIPDTMTSWIVIFFLPVNSALNPILY

**RXFP2_Chicken**  AAFITIVFAYTGMFYSIRITTCNVCPRDVAVAKRFFFIVFTDALCWIPIFLLKLLSLLQVEIPGTVTSWVVIFILPINSALNPILY

**RXFP2_Lizard**  VAFLLVSFAYGGMFHSIHATAASILSVEVALAKRFFFIVFTNALCWIPIFILKLLSLLDVDIPGTVTSWVVVFILPINSTLNPILY

**RXFP2a_Frog**  LAFIIIVFSYISMFCSIQKTALSHIHTDVAVANRFFFIVFSDAVCWIPVFLLKILSLFRVEIPGTVTSWIVIFILPINSALNPILY

**RXFP2b_Frog**  LAFVTIVFSYSSMFYSIHKTGASVLSREVTIAKRFFFIVFTDALCWIPIFLLKAVSLTEAEIPGTITSWIVIFILPINSALNPILY

**RXFP2a_Coelacanth** LAFITIVFSYVSMFYSINKTGISHIRRDVAVANRFFFIVFSDAICWIPIFLLKILSLLNVEIPGTITSWVVIFILPINSALNPILY

**RXFP2b_Coelacanth** LAFIIIVFSYSSMFYSVHNTGASVFSREVAIAKRFFFIVFTDALCWIPIFLLKTLSLLQVETPGTITSWVVIFILPINSALNPILY

**RXFP2_Fugu**  VAFLIIVFSYSSMFYSIYKTGISRLHRDVAVANRFFFIVFSDALCWIPIFLVKILSLLEVEIPDTISSWVVIFILPINSALNPILY

**RXFP2_Medaka**  VAFLVITMSYSSMFYNIYKTGISRLHKDVAVANRFFFIVFSDALCWIPIFLVKVLSLLEVEIPGTISSWVVIFVLPINSALNPILY

**RXFP2a_Zebrafish**  VAFLVIVISYSSMFCSIYKTGISRLHKDVAVANRFFFIVFSDALCWIPIFLVKTLSLMKVEIPGTINSWVVIFILPINSALNPILY

**RXFP2b_Zebrafish**  LAFLIIVVSYSSMFYSIYKTGIGRLHRDVAMAHRFFFIVFSDALCWIPIFMVKILSLLEVEIPGTITSWVVIFILPINSALNPILY

**RXFP2c_Zebrafish**  LAFVMIVFSYSSMFYSVQKTAKTVYDREVTIAKRFFFIVFTDALCWIPIFLLKILSLLRVQIAGTIILWVVIFILPINSALNPILY

**RXFPa_Amphioxus**  SSLLVIMAAYIGMFISIQRTRSFSLLSDMSFAKRFFFIVLTDSLVWLPITAIKFMALTSGPISGTTYAWIVVFVLPINSAVNPILY

**RXFPb_Amphioxus**  VSVLVIGAAYVGMFISIQRTRQLTLFTDMSFAKRFFFIVLTDSLVWLPITIIKFIALAGIPIPGTMYAWIVIFVLPINSAINPILY

**RXFPa_Urchin**  TTFFVIVVSYTAMFLSIRKTRRCGVKDDMAYAKRFFFIILTDSLCWLPIAILKIMSLYNYIISETLYGWIVVFVLPINSALNPILY

**RXFPb_Urchin**  TAFVAIALSYTGMFLSIRKTRRCGMKGDMAYAKRFFFVILTDSLCWLPIAVLKVMSLCNFIISETLYGWIIVFVLPINSALNPILY

**RXFP_Oyster**  AAFSFICFAYIHMFIAIKRSTKHFENKERTLVKRFFFIILTDFLCWMPIIIIKFVALSGYKINQDTYAWLIIFVMPINSAINPLLY

**RXFP_Limpet**  ASFLFISYAYLAMFVSIKHTEVTRESRERCLVKRFSFIVITDFICWIPIIIIKVVALSGVKISGDLYAWVVVFILPVNSALNPLLY

**RXFP_FruitFly**  LLLVMIAMLYTALLISIWRTRSPLTLLDCEFAVRFFFIVLTDFLCWVPIIVMKIWVFFNYNISDDIYAWLVVFVLPLNSAVNPLLY

**RXFP_Daphnia**  SCVLVIVGVYTALFVSIRRTRTPLAPNEIEIAVRFFFIVFTDCLCWMPTILLKIMALANVYIPADLYAWLVVFILPVNSAINPLL-

**RXFP_Mite**  AAMLLISMLYAWMFVIIRNDRQLKRREDSVLAFRFFLIVLTDCLCWIPIIVIKLAALCNVKISPDVYAWVVVFVLPINSALNPVIY

**RXFP_Tick**  FAMVLVMGLYLSMFCIIKEDRQMKKQEDAVLALRFFFIVLTDCMCWIPIVIIKILALLEVQISENIYAWVVVFILPINSALNPVIY

**RXFPa_SeaAnemone**  IAFIFILVAYMTMFWTVKRVSGTSMNKESAMAKKLIFIISTDFCCWMPVIVIGILSLTGAFYDPQVYAWIAVFVLPVNSSINPILY

**RXFPb_SeaAnemone**  IAFMFILLAYIAMFWTVRKSSARNAKKESALARKLMVIILTDFCCWMPVILIGILSLTGNFHDPLAYVWIAVFVLPVNSSINPILY

**RXFPa_Coral**  VSFLYILVAYIVMFMTVKNSSKTNMKRESQMARRMFFIILTDFLCWMPVILIGLLSVLGKFHDPQAYIWIAVFVLPVNSSINPFLY

**RXFPb_Coral**  VSFVYILVAYIVMFMTVKNSSKTNLKRESQMARRMFFIILTDFLCWMPVILIGLLSLLGKFHDPEAYVWIAVFVLPVNSSINPILY

**GRL101_Urchin**  VSFITIMVSYGVMFNVARKTQKLKSKGSDSMARRMSVIVFTDFCCWVPIILLGLASLSGAYVPTSVYAWVAVFVMPVNSAVNPILY

**GRL101_Oyster**  LSFLVIFISYLWMFLVAKQTTTSKAKSDNSMAKRMTLIVMSDFFCWVPIILLGFASLGGATVPPQVYAWVAVFVLPLNSAMNPVLY

**GRL101_Limpet**  ISFLLIFLSYFWMFTVAKRTRSTETKTDAAMARRMTMIVMTDFFCWVPIILLGYASLGGASIPQNVYAWVAVFVLPLNSAINPVLY

**GRL101_Louse**  ISLILIAMGYIWMYGAAKNTRLLSKRMEQVMARRMIFIVATDAACWVPVILLGILSLNGVSVPSQVFAGIAVFVLPLNAAVNPIFY

**GRL101_SeaAnemone** ISFMAIFILYFIMFIKIKQSHQSQRSATASIGSRMVFIVLTDFVCWIPIIIIGIASLSGMQAPPEVYAWVAVFVLPLNSALNPILY

**GRL101_Coral**  ASFMIIFFLYLIMFLKIQKTRK-AGPAVSSIGSRMVFIVLTDFCCWIPIIIIGIASLV------------------------DDRN

**GRL101a_Placozoan** VGCLYILGAYIAIFIKLHQNKKHTNKQDRVVTRNMVLIVGTDLCCWIPIIIMTYLTLANIPIDKTIFAWVAVFVLPLNSAVNPIIY

**GRL101b_Placozoan** IACMFILLAYLALMIKLKTR-KRSARQDRVVTVKMILVIGTNLCCWLPIIAITFLALFKIHVPRETIAWIAVFVLPLNSAMNPVIY

**OpsB_Human**  VPLSLICFSYTQLLRALKAVAATTQKAEREVSRMVVVMVGSFCVCYVPYAAFAMYMVNNRNLDLRLV-TIPSFFSKSACIYNPIIY

**Ops1_FruitFly**  IPLFLICYSYWFIIAAVSAHEK-EKSAEGKLAKVALVTITLWFMAWTPYLVINCMGLFKFELTPLNT-IWGACFAKSAACYNPIVY

**OPS3_FruitFly**  CPTTMITYYYSQIVGHVFSHEKNKETAEIRIAKAAITICFLFFCSWTPYGVMSLIGAFGDKLTPGAT-MIPACACKMVACIDPFVY

**ACM1_Human**  LPVTVMCTLYWRIYRETENRARFSLVKEKKAARTLSAILLAFILTWTPYNIMVLVSTFCKDVPETLW-ELGYWLCYVNSTINPMCY

**n5HT7R_Human**  IPMSVMLFMYYQIYKAARKSAASIFKREQKAATTLGIIVGAFTVCWLPFFLLSTARPFCGTIPLWVE-RTFLWLGYANSLINPFIY

**n5HT1R_FruitFly**  IPLSVMLFVYYQIFRAARRIVLFQLAKEKKASTTLGIIMSAFTVCWLPFFILALIRPFETMVPASLS-SLFLWLGYANSLLNPIIY

**n5HT1A_Human**  IPLLLMLVLYGRIFRAARFRIRMALARERKTVKTLGIIMGTFILCWLPFFIVALVLPFCESMPTLLG-AIINWLGYSNSLLNPVIY

**n5HT1B_Human**  FPTLLLIALYGRIYVEARSRILLMAARERKATKTLGIILGAFIVCWLPFFIISLVMPICKDFHLAIF-DFFTWLGYLNSLINPIIY

**DRD1_Human**  IPVAIMIVTYTRIYRIAQKQIRMSFKRETKVLKTLSVIMGVFVCCWLPFFILNCILPFCGSIDSNTF-DVFVWFGWANSSLNPIIY

**ADRB1_Human**  VPLCIMAFVYLRVFREAQKQVKLVALREQKALKTLGIIMGVFTLCWLPFFLANVVKAFHREVPDRLF-VFFNWLGYANSAFNPIIY

**ADRA1D_Human**  LPMAVIVVMYCRVYVVARSTTRLKFSREKKAAKTLAIVVGVFVLCWFPFFFVLPLGSLPQLPSEGVF-KVIFWLGYFNSCVNPLIY

**Fig. S3D.** Sequences used in the alignment presented in fig. S3C.

| **Receptor** | **Common Name** | **Species** | **Accession** | **Database** |
| --- | --- | --- | --- | --- |
| 5HT1A | Human | *Homo sapiens* | NP_000515.2 | NCBI |
| 5HT1B | Human | *Homo sapiens* | NP_000854.1 | NCBI |
| 5HT1R | Fruit Fly | *Drosophila melanogaster* | NP_524599.1 | NCBI |
| 5HT7R | Human | *Homo sapiens* | NP_000863.1 | NCBI |
| ACM1 | Human | *Homo sapiens* | NP_000729.2 | NCBI |
| ADRA1D | Human | *Homo sapiens* | NP_000669.1 | NCBI |
| ADRB1 | Human | *Homo sapiens* | NP_000675.1 | NCBI |
| BursR | Annelid Capitella | *Capitella teleta* | 226336 | JGI |
| BursR | Coral | *Acropora digitifera* | adi_v1.05605 | OIST |
| BursR | Daphnia | *Daphnia pulex* | EFX85901.1 | NCBI |
| BursR | Fruit Fly | *Drosophila melanogaster* | NP_476702.1 | NCBI |
| BursR | Hydra | *Hydra magnipapillata* | XP_002169896.1 | NCBI |
| BursR | Limpet | *Lottia gigantea* | 115178 | JGI |
| BursR | Louse | *Pediculus humanus* | XP_002423467.1 | NCBI |
| BursR | Mite | *Metaseiulus occidentalis* | XP_003737324.1 | NCBI |
| BursR | Oyster | *Crassostrea gigas* | EKC26266.1 | NCBI |
| BursR | Red Flour Beetle | *Tribolium castaneum* | XP_975514.1 | NCBI |
| BursR | Sea Anemone | *Nematostella vectensis* | XP_001635321.1 | NCBI |
| BursR | Tick | *Ixodes scapularis* | XP_002407612.1 | NCBI |
| BursR | Urchin | *Strongylocentrotus purpuratus* | XP_782167.3 | NCBI |
| BursRa | Placozoan | *Trichoplax adhaerans* | XP_002107746.1/JGI_51554 | NCBI |
| BursRb | Placozoan | *Trichoplax adhaerans* | XP_002107856.1/JGI_51783 | NCBI |
| CKHR | Comb Jelly | *Mnemiopsis leidyi* | ML08388a | NHGRI |
| CKHR | Sponge 3 | *Oscarella carmela* | g9064.t1 | COMPAGEN |
| CKHRa | Sponge 1 | *Ephydatia muelleri* | m.193551 | COMPAGEN |
| CKHRa | Sponge 2 | *Amphimedon queenslandica* | XP_003385791.1 | NCBI |
| CKHRb | Sponge 1 | *Ephydatia muelleri* | m.83304 | COMPAGEN |
| CKHRb | Sponge 2 | *Amphimedon queenslandica* | XP_003385642.1 | NCBI |
| DRD1 | Human | *Homo sapiens* | NP_000785.1 | NCBI |
| FHSR | Human | *Homo sapiens* | NP_000136.2 | NCBI |
| FSHR | Chicken | *Gallus gallus* | NP_990410.1 | NCBI |
| FSHR | Frog | *Xenopus tropicalis* | XP_002935422.1 | NCBI |
| FSHR | Lizard | *Anolis carolinensis* | XP_003216196.1 | NCBI |
| FSHR | Medaka | *Oryzias latipes* | NP_001188443.1 | NCBI |
| FSHR | Zebrafish | *Danio rerio* | NP_001001812.1 | NCBI |
| GPHR | Acorn Worm | *Saccoglossus kowalevskii* | XP_002739238.1 | NCBI |
| GPHR | Amphioxus | *Branchiostoma floridae* | XP_002610242.1 | NCBI |
| GPHR | Annelid Capitella | *Capitella teleta* | 139301 | JGI |
| GPHR | Aplysia | *Aplysia californica* | XP_005091539.1 | NCBI |
| GPHR | Coral | *Acropora digitifera* | adi_v1.10706 | OIST |
| GPHR | Daphnia | *Daphnia pulex* | EFX71271.1 | NCBI |
| GPHR | Fruit Fly | *Drosophila melanogaster* | NP_524393.2 | NCBI |
| GPHR | Hydra | *Hydra magnipapillata* | XP_002155960.1 | NCBI |
| GPHR | Limpet | *Lottia gigantea* | 92538 | JGI |
| GPHR | Louse | *Pediculus humanus* | XP_002429811.1 | NCBI |
| GPHR | Mite | *Metaseiulus occidentalis* | XP_003741293.1 | NCBI |
| GPHR | Oyster | *Crassostrea gigas* | EKC35054.1 | NCBI |
| GPHR | Red Flour Beetle | *Tribolium castaneum* | XP_968907.2 | NCBI |
| GPHR | Sea Anemone | *Nematostella vectensis* | XP_001641580.1 | NCBI |
| GPHR | Silkworm | *Bombyx mori* | NP_001037033.1 | NCBI |
| GPHR | Urchin | *Strongylocentrotus purpuratus* | XP_782262.3 | NCBI |
| GPHRa | Lamprey | *Petromyzon marinus* | AAW80618.1 | NCBI |
| GPHRb | Lamprey | *Petromyzon marinus* | AAW80619.2 | NCBI |
| GRL101 | Coral | *Acropora digitifera* | adi_v1.02959 | OIST |
| GRL101 | Limpet | *Lottia gigantea* | 142042 | JGI |
| GRL101 | Louse | *Pediculus humanus* | XP_002423759.1 | NCBI |
| GRL101 | Oyster | *Crassostrea gigas* | EKC37058.1 | NCBI |
| GRL101 | Sea Anemone | *Nematostella vectensis* | XP_001624838.1 | NCBI |
| GRL101 | Urchin | *Strongylocentrotus purpuratus* | XP_789319.3 | NCBI |
| GRL101a | Placozoan | *Trichoplax adhaerans* | XP_002112613.1 | NCBI |
| GRL101b | Placozoan | *Trichoplax adhaerans* | XP_002116101.1 | NCBI |
| LHCGR | Chicken | *Gallus gallus* | NP_990267.1 | NCBI |
| LHCGR | Coelacanth | *Latimeria chalumnae* | ENSLACP00000012918 | ENSEMBL |
| LHCGR | Frog | *Xenopus tropicalis* | XP_002935423.1 | NCBI |
| LHCGR | Human | *Homo sapiens* | NP_000224.2 | NCBI |
| LHCGR | Zebrafish | *Danio rerio* | NP_991188.1 | NCBI |
| Ops1 | Fruit Fly | *Drosophila melanogaster* | NP_524407.1 | NCBI |
| Ops3 | Fruit Fly | *Drosophila melanogaster* | NP_524411.1 | NCBI |
| OpsB | Human | *Homo sapiens* | NP_001699.1 | NCBI |
| RXFP | Daphnia | *Daphnia pulex* | EFX79619.1 | NCBI |
| RXFP | Fruit Fly | *Drosophila melanogaster* | NP_733115.1 | NCBI |
| RXFP | Limpet | *Lottia gigantea* | 108234 | JGI |
| RXFP | Mite | *Metaseiulus occidentalis* | XP_003737043.1 | NCBI |
| RXFP | Oyster | *Crassostrea gigas* | EKC17803.1 | NCBI |
| RXFP | Tick | *Ixodes scapularis* | XP_002400885.1 | NCBI |
| RXFP1 | Coelacanth | *Latimeria chalumnae* | ENSLACP00000003987 | ENSEMBL |
| RXFP1 | Frog | *Xenopus tropicalis* | XP_002934794.1 | NCBI |
| RXFP1 | Fugu | *Xenopus tropicalis* | XP_003970701.1 | NCBI |
| RXFP1 | Human | *Homo sapiens* | NP_067647.2 | NCBI |
| RXFP1 | Lizard | *Anolis carolinensis* | XP_003221751.1 | NCBI |
| RXFP1 | Medaka | *Oryzias latipes* | XP_004086569.1 | NCBI |
| RXFP1 | Zebrafish | *Danio rerio* | NP_001177863.1 | NCBI |
| RXFP1a | Chicken | *Gallus gallus* | XP_420385.2 | NCBI |
| RXFP2 | Chicken | *Gallus gallus* | XP_426253.3 | NCBI |
| RXFP2 | Fugu | *Takifugu rupribes* | XP_003979236.1 | NCBI |
| RXFP2 | Human | *Homo sapiens* | NP_570718.1 | NCBI |
| RXFP2 | Lizard | *Anolis carolinensis* | XP_003227257.1 | NCBI |
| RXFP2 | Medaka | *Oryzias latipes* | XP_004076336.1 | NCBI |
| RXFP2a | Coelacanth | *Latimeria chalumnae* | ENSLACP00000007110 | ENSEMBL |
| RXFP2a | Frog | *Xenopus tropicalis* | XP_002934071.1 | NCBI |
| RXFP2a | Zebrafish | *Danio rerio* | ABV48903.1 | NCBI |
| RXFP2b | Coelacanth | *Latimeria chalumnae* | ENSLACP00000014853 | ENSEMBL |
| RXFP2b | Frog | *Xenopus tropicalis* | XP_002938964.1 | NCBI |
| RXFP2b | Zebrafish | *Danio rerio* | ABV48904.1 | NCBI |
| RXFP2c | Zebrafish | *Danio rerio* | XP_001919542.3 | NCBI |
| RXFPa | Amphioxus | *Branchiostoma floridae* | XP_002611689.1 | NCBI |
| RXFPa | Coral | *Acropora digitifera* | adi_v1.19513 | OIST |
| RXFPa | Sea Anemone | *Nematostella vectensis* | XP_001626505.1 | NCBI |
| RXFPa | Urchin | *Strongylocentrotus purpuratus* | XP_793379.3 | NCBI |
| RXFPb | Amphioxus | *Branchiostoma floridae* | XP_002611688.1 | NCBI |
| RXFPb | Coral | *Acropora digitifera* | adi_v1.17524 | OIST |
| RXFPb | Sea Anemone | *Nematostella vectensis* | XP_001637653.1 | NCBI |
| RXFPb | Urchin | *Strongylocentrotus purpuratus* | XP_784249.2 | NCBI |
| TSHR | Chicken | *Gallus gallus* | NP_001180517.1 | NCBI |
| TSHR | Coelacanth | *Latimeria chalumnae* | ENSLACP00000018752 | ENSEMBL |
| TSHR | Frog | *Xenopus tropicalis* | XP_002938419.1 | NCBI |
| TSHR | Fugu | *Takifugu rupribes* | XP_003971550.1 | NCBI |
| TSHR | Human | *Homo sapiens* | NP_000360.2 | NCBI |
| TSHR | Lizard | *Anolis carolinensis* | XP_003214472.1 | NCBI |
| TSHR | Medaka | *Oryzias latipes* | XP_004083585.1 | NCBI |
| TSHR | Zebrafish | *Danio rerio* | NP_001139235.1 | NCBI |

**Fig. S4.**  LGR sequences featured in Fig. 5. Signal peptides have red text and cysteine residues in the N-terminal and hinge regions are highlighted in black. LRR regions are highlighted in yellow, N-terminal hinge motifs are highlighted blue, C-terminal hinge motifs are highlighted green and the seven-transmembrane region is highlighted gray.

**>GPHR_FruitFly_NP_524393.2**

MEKHPSLSQRMGTTYRPRKGLKCLSFEFQCRLLLHHLLLTSLSGRHFVYATSAVGGALSA

NNCHDIHHGFDVYPNLTAVSLAQSTDTPLTATMPRSAWKCCCWNASNQAEEVECRCEGDG

LNRVPQTLTLPIQRLTIASAGLPRLRHTGLKVYGSTLLDVAFTDCLQLELIQDGAFANLT

LLRTIYITNAPKLTFLSKDVFLGISDTVDIIRIINSGLTRVPDLGHLPPHNILQMIDLDN

NQITRIDSKSIKVKTAQLILTNNEISYVDDSAFFGSKIAKLSLKENKKLQMMHPNAFDGI

IDITELDLSSTSLVGLPSAGLQNIEALYIQNTHTLKTIPSIYNFRNLQRAYLTHSFHCCA

FQFPSRHDPQRHAQRMLEIEKWRKQCKSDSGTRKERSTLDNPFNMPEDFGSFGGTDDSAT

DITPITFASFDYMADDTMNKGTFHEKIILNPGDDSSAELCGNFTFRKPNIECYPMPNDLN

PCEDVMGYQWLRISVWIVVALAVVGNVAVLTVILSIRPESTPVPRFLMCHLAFADLCLGL

YLLLVACIDAHSMGEYFNFAYDWQYGLGCKVAGFLTVFASHLSVFTLTVITIERWLAITQ

AMYLNHRIKLRPAALIMLGGWIYSMLMSSLPLFGISNYSSTSICLPMENRDVYDTIYLIA

ILGSNGVAFSIIAVCYAQIYLSLGRETRQAHQNSPGELSVAKKMALLVFTNFACWSPIAF

FGLTALAGYPLINVTKSKILLVFFYPLNSCADPYLYAILTSQYRQDLFTLLSKLGLCQQS

ALKYKDSLSGQATTRFTIHGSIQRHSSLTCKMQTVMGAETQKMLKNSEDYV

**>GPHR_SeaAnemone_XP_001641580.1**

MGRGRVCSLLLAFLLVAFGAAQPDVCKELCSCSLDAVHCSQLTVFPPIRTFPANISTLTI

SYSPKAAIRIEELAELPYLTSLNLVYNKITNVSKFVSSNLRVLNLDHNQIESVEPGAFDS

LENLLDLSLANNKLKSFPVFRKRSSITRLFVGGNIGITSLDKQSLYNLPRLQLLSLKSSG

LTSLPYAIFRRNKELQALLLSRSVSLTTINEYAFDGLKTLTKLDLSYTRVTTIPNQGLEY

LEKIYLKGVPDFWVLPATLWNIREVHLDQYNSFLCCAFYLQRGTPDCTYLSGQTGDVPTY

LPITDLETTSSTSTNAATSPTRFGTPSSGFGTPPSGFGTPPSGFGTPPAGFGTPPSGFGT

PPSGFGTPPSGFGTPPSGFGTPPAGFGTPPSGFGTPPSGFGTPPSGFGTPPSGFGTPPSG

FGTPSSGFGTAPSGFGTPPGGYGNRSTPYRPIITLPPGFHNVTSKVHSTIPHENKTNASA

YRCPDTRPLVKCFPKPDAFHPCEDIMGAAWLTTFSFLVGGMAVVANFVVALVLLVSERRL

NVTRFLMCNLAFADFCLGLYLFILTCVSMDTHGLYHNYVRRWQYGAGCKLTGFLAVFATE

LSVYTLVLITLERFYAIVYAMQLNTRLSMRMTVRAMAAGWVAAVLLASLPLMGASSYSKV

AICLPFDVSTTGSIAYVAFLLFLNGAAFAFVLYLYMRMLMTVISGGDMEGAPKRDDSKVA

KRMALLVLTDFVCWAPIAFFGILAAFGTPLIDVTASKTLLVFFFPINSLCNPFLYAFFTK

AFKRELFVLLSRCGFCRRRALKYSGTLSSLMYSRRK

**>BursR_FruitFly_NP_476702.1**

MAARCRWSWRLALCPLLLQLLLQLLLLPPSAMGHDETKENPAPDMQNSQEQEPYVHLQHL

QQQQQQNPQTVQQLSQITVNRTSKSASVTPTGIRENVMLPSADPEKEAQILYEKSLQEYH

GSQLSTASTATDVIAGKRTLHSICERWLQKHCHCTGSLEVLRLSCRGIGILAVPVNLPNE

VVVLDLGNNNLTKLEANSFFMAPNLEELTLSDNSIINMDPNAFYGLAKLKRLSLQNCGLK

SLPPQSFQGLAQLTSLQLNGNALVSLDGDCLGHLQKLRTLRLEGNLFYRIPTNALAGLRT

LEALNLGSNLLTIINDEDFPRMPNLIVLLLKRNQIMKISAGALKNLTALKVLELDDNLIS

SLPEGLSKLSQLQELSITSNRLRWINDTELPRSMQMLDMRANPLSTISPGAFRGMSKLRK

LILSDVRTLRSFPELEACHALEILKLDRAGIQEVPANLCRQTPRLKSLELKTNSLKRIPN

LSSCRDLRLLDLSSNQIEKIQGKPFNGLKQLNDLLLSYNRIKALPQDAFQGIPKLQLLDL

EGNEISYIHKEAFSGFTALEDLNLGNNIFPELPESGLRALLHLKTFNNPKLREFPPPDTF

PRIQTLILSYAYHCCAFLPLVAMSSQKKTSQVQEAVLFPSDAEFDMTLWNNSMMNIWPQM

HNLSKQLGASMHDPWETAINFNEEQLQTQTGGQIATSYMEEYFEEHDVSGPATGYGFGTG

LFSGMSTEDFQPGSVQCLPMPGPFLPCADLFDWWTLRCGVWVVFLLSLLGNGTVVFVLLC

SRSKMDVPRFLVCNLAAADFFMGIYLGILAIVDAATLGEFRMFAIPWQMSVLCQLSGFLA

VLSSELSVYTLAVITLERNYAITHAIHLNKRLSLKQAGYIMSVGWVFALIMALMPLVGVS

DYRKFAVCLPFETTTGPASLTYVISLMFINGCAFLTLMGCYLKMYWAIRGSQAWNTNDSR

IAKRMALLVFTDFLCWSPIAFFSITAIFGLQLISLEQAKIFTVFVLPLNSCCNPFLYAIM

TKQFKKDCVTLCKHFEESRVVGGGGPGGRGAVARTKRGDLPPPLLPAAAVAHPPGCRCLR

MLPSEMPNWHKMEQTPSMWQRLRTFCCGENRRRRKQRRQPQQRRQRAYTAAAANPYQYQF

AELRQQRQNRASSISSENFCSSRSSSWRHGPPSSAPVPPGNCSMPLKMLEPHAHPHGHGR

RRHSAWLITRKTSQDSNLSSSRNDSSASATTASTSTFRLSRSSAGSSTPLPSIIAHNGKA

QLDAVKPRLVRQEAVQEEEDSSPPRLGVRFLPTIPSAADSSVVMEDGDSANTGVASFLGM

PLPGASSGFLIAPTTAATTSPPPVVLQPAKPPPDPNDAPL

**>BursR_SeaAnemone_XP_001635321.1**

MSLVFGTQQAKELLVINSKQHRDQQSIFRNTFFPYCCTLFRFLDYNDLTVVPDGLKALSR

LQHLNLNFNRISKVPRNAFNKLRLLVELFLDYNDLTVVPDGLKALSRLQHLNLNFNRISK

VPRNAFNKLRLLVELNLRGNKLKSIPTSAINDLMRLQQLELDGNPIRVIKDHAFKGLFRL

TEIALGWEKLEEVYHNAFTELPLLRRLPLGNVKLKHFPNLTGTHSLQILTLESNYIEYLP

RNFCGMFPKLYDFNANMNKIKRIPDMSGCKSMELLKLVHNQISSIGSSLQGMARLKDLTL

EGNRITEVNDNTFKGVKSLETLDLAKNQIKHISKNAFSHFERLQTLDLSENLFVRLPSAG

MQQIRRLYLRGNRHLKELSVQDLPRVHTVVAAYPYHCCGFRKKVNNRKITGNGEAVVSEG

AWYWTKEHEYLRLDRPGNSSLTLDDDDLSEFGSGKVDAALGLDTEFSGNGSEPAVYNSTI

VGSADSILGELDDWQREVNCTPLPDPFFPCEDLMGSWLLRMGVWIVFMLALLGNVTVIIV

ILVSKTKMDVSRFLIVNLAVADMCMGLYLGLLAIVDASTIGDFLHHGVEWQLSTGCKTAG

FLALLSSEASVFTLTVITIERFIAIRHALHIHKKMSLRKTTIVMVIGWGLALIIATLPLA

KVSDYTKVSVCLPFEIGEIESLTFVTFTMVLNCAAFLVIFGCYVGIYLQVRGSNAWNTND

TQVALRMSLLVVTDLMCWAPIAFLALTATFGTTFVSLNEAKVFTVFCFPLNSCANPFLYA

IFTSQFKKDCLTICRRVKNSPVPPKIHITLSKKRFSVSGAALERGSNASAQVPSRDTNSD

GRLLMLRLSNVIVNRRFSLPPEVRLGTGNKLKTIPQESSRSLTNSPSSCSNANSTEQTTL

NPEKRRMIIEMETAL

**>GPHR_CombJelly_ML08388a**

MLLFLLLVFTISGALGAACGGHYPSRCKCEFDSHGKVRSVNCTDAAISQFPDIDVSVRTL

DLSGNKITHLRRKNGGKVYNDLLSLTLNDCQIKTIEEGFFLQFPSVNIVKLKGNKLTRLP

DISSSSSVILDMTYNHISKLNNFSNPTLHSLYLDFNLLTVLKEHDLNNLPSLKVLTLMFN

SIEKIHKDALSPAISHLSLGSNKLREVPVLEKLSASLLSLSLEKNKLLNFDNVLRYPNLE

NCTLASNAITYFPKRKSREFLSAIRVLVLSRNSIDQINRADFEMFPNLYYLELSENAISH

IPDHCFSSLPLLRKLDLSDNSIKNVSETSLKRTERLETLYLQQNSIINLKLPLNLLNLHE

LDVTGNKLTEPPNCPPSLLLLYIAENNIDKLQSEHFASARRLKDLDVSNNQIRKIDEDLF

YLLPELERLHIQNNLLSTVDVLDLSSNIYLTMLNLGNNGLKHLPDNFLDKTSVTTLSLPN

NQLTKLTNTTLAGVEGKLRFLDLSFNDLEELSHVGLSAVESLLLEGKTPKTKKKRRRQKS

LCLATGKMRPPLITRQRSLLLSNSLVLSPSVLRTSTKTGRTGQFYNEFQLLMRFTPTRFL

IVNLSFSDLLTGIYLMMLAAVDAKTYGFYEDYAVDWQNGPGCQIAGFMVTFSSELSVFTL

TMISVERMVTIIHYSNPYKHLKTRHIALAMLLGWLISATAGAFPLLGFSDYSLVAICLPF

QNSDGGDLALILSLMVINTIAVMVIIYCYLTIFCNVRHLQKPLDNADFRETLRMALIILT

DVLCWLPIVVCGVSAAVGKPLITTSNAKILVVFFLPLNACANPFLYSLSRAGFKKDFLCV

LDKMGLCKQAYYIASRRKSGMDLDLKKKLQAETKELRKPSQDSTSTTRSSSGYSSDSSYI

KHLVNQFKSNIGSLEETRDFDT

**>GPHRa_Sponge1_m.193551**

MITAAVGRVASIVLSLLISCGRVTTSTSTRCFDAESSFQKQGCVCNDFEQYTFISCAFLN

GELPTSMSLNYSYVSGLSIHGGTLTSLKRSNFNFFPSLTQLSIQNTLLQHIDPDAFKDVT

LTSITLENSRLTEFPPVANLSRLHILSVKGNRISSITKANLLNTVSLLTLDLESNQITEL

PVDVFENTCLLATLSLSNNNIKEFRYGTFRHTTITSLDLSFTPVEQLPTDGIRDHLSEIN

LKGATHFTYPSFTENDATHGNLTYPVLNKITMPFHTFCCQLRSQRIRPLQIKKSNSKRQG

SLPQPTPSPSTSLNSIHSTSSLVSNCITSTVVQTTCRTQAVSLSPTVAPAQLSTSATLMT

FTALDGATVISATPTPSFFTSTTTSFSSRASCSNLWTASGGQCISSTLLFAPTTTTSLPS

EVVSYVLDISGTQDIAQSTASVIFVTPVAPSSLPIMTSPSVSDMSTVPNPATGTDLSFTT

TSFLPTDSLSATPMVSCYTETVVEVCCNLPPVDCSARCNNDPADPVFCLTCYGSGCIDAY

CLSTAGCICAAEQSSISGNTSAVVCTTVNSSLESPSSTAPSVHETPAPPILTESCTESVP

TYESTNNEYIECIPEEDPFNPCEELLSDGIRVVIWIVITITLLGNGTVVTCMMAYGLFFR

HKQKHLQVVNFFYLNLATADFLMGVYLFTIGTEDLNTHYDYPGAFFTQSAAWQSSPGCNF

AGFCAMTSISMSVFTLVLITLERVHSIVQVFEQRKMSMKLAALLMAAGWVFAVVMGALPI

AHVSDYGKTAVCLPFDSSSAVSAGYVYFVLIATGVASLVITTSYIVIGCNVFYNRQKGYP

ANASKMNECYVAIRMSVIVLSNFACWFPIAVVGLGSATGQKWIGLDDAKVFMVVVFPLNA

SLNPIIYTLSTRVFRETLIMMGKKCHLADFSRRASHLHSRETSTLRRLSLSKTSTTDLFA

SSSGSRSNVSLSDPAKIEATMGTTLGTTNIISLASIREEEFEDPQGLVIRNDVAARGSEQ

YELVVETSPESTSMETSFAGIIENRAHSEE

**>GPHRb_Sponge1_m.83304**

MQISPVISLMRLLCLGLLLTAVATVSGVSPDVCSVCHCTPLTINCSSQGLLEIPFFGNET

EIQRLDLSGNNIRNVPSSSLRGIAGTLQLISLANNQITEIEDGTFADMPFLQAVVLKQNR

LSSLPENVFGHRVIATLDLSYNNFGAVPSASLRNLAGVQSLFLNNNPLQSLSDTTFVNVS

GIQELDLSYCNLSNENFNPKALDKKIFGFLQKLTLDGNYFTVLNAEFFKAFVPSLVTLSL

QYCGIKTLKHGSFDVFNHLQTLYLDYNYIEVLEKNIFYGIVLTTLSIQYCRITTIHRGAF

NKSSIETLKLNYNYLSRIRRVDFDGLYDMKNLELSHNKISRIIGKPFLRQHNILLDHNEL

VDASFGDPNVPNSVEPLADLSHLEVIDISHNQLTRLPNLHASESKLLQLLAHHNQITDLS

ETDLSGMSILVILDLSSNKITRISSSALSNCVNLYKLNLANNLFFQLPSLKNQTVLDQLF

ISNCKIEALPNDLCLTCPLLGTLSVEYNQLKSIPNLSSCKNLREVLLENNLLTALDESTF

NGLSGLHTIDLSYNTISSLTNGTFRGLVNLKSLHLDYNAIRELPSGIFEDLVNLNRIDLN

NNKIQSLPDNLFQRTNIIAEIRLNNNEIKYVSSIAFSNNLTYLQRLNLSSNMFSEWTFPE

GGFRSLLYLSLANVEQLYQAPDPIQCDCPGVQLLEFTYPYHCCLWQDRVNPKNLKPTTTP

SNEDGNFTLPVYNPTFPQILTVIPPFHSGNGANIATLIPILDWFSEAYGVDYRILPGPII

IWYFSNSTRVYTDITPPPPTVQSLVGSRSVMCSPPGDALRPCDNLMSPWPLRAAIWFILV

LALLGNGSVLFVMLASKDKIKVPQFFISNLAFADFCLGIYLAFLACVDARTYGNQFYQSA

LEWQRGPGCSTAGFIAVFSSMLSCSMLVAVTLERVYTICHSPKTGISMSTAIVVALFCWL

ISFAFATLPLVGINSYSRVAVCLPFVTAELRDKIYIGVLLTTTMVAFLIILVSYIVILCI

VCRSPAVGQNRKETVKLLRKMAPLVITNFLCWFPIVVIGYSALADKPLIGVSQAKWLVVL

VYPFNACANPFWYAIFTKNFRHRMRSMFKHTTTYLQTPNAIGLRIHLGHNSKSRSINDLD

EDEKRRRRQSQRSRSVVAYTHTAVFGTLHGQEAPAEPYMGRRASLPAVLRQSECANVPAD

SLLFQMRMPHTTQNNSLPNVFEATKCHEVETSLTHGSDSSRLHKSLSVLKEIPEAEDMDD

DKKSTVSTQSNEYHDAADHIAEAHYCSHRQVTQDLRSLDSCCSSASNAVTETSLGSTQHL

TSVIPHTTMKYTLDCNSSFIGSETEV

**Fig. S5.** Synteny orthologs in Fig. 5 found in amphioxus, human, limpet and sea anemone. ‘Loc.’ indicates the scaffold (or chromosome in human) on which the gene model is found, followed by its specific location in megabases. JGI PID indicates a protein ID from the respective JGI genome, and ALG indicates the conserved ancient chordate linkage group to which the ortholog belongs.

| **Ortholog** | **Amphioxus Loc.** | **JGI PID** | **Best Human Hit** | **e-value** | **Human Loc.** | **ALG** |
| --- | --- | --- | --- | --- | --- | --- |
| CKDL1/4 | 25:2.2 | 275907 | NP_004187.2 | 0 | 14:50.8 | 11 |
| MAPK3/5 | 25:2.2 | 117888 | NP_003609.2 | 0 | 2:39.5, 14:50.9 | 11 |
| ISM1/2 | 25:1.5 | 202308 | NP_543016.1 | 4e-81 | 20:13.2, 14:77.9 | 11 |
| GPHβ5 | 25:2.5 | 202529 | NP_660154.3 | 1e-28 | 14:63.8 | 11 |
| GPHα2 | 25:2.5 | 117901 | NP_570125.1 | 1e-10 | 11:64.7 | 11 |
| SAMD4A/B | 25:1.0 | 202549 | NP_056404.4 | 0 | 14:55.0, 19:39.8 | 11 |
| ACTN | 25:1.3 | 275861 | NP_001265272.1 | 0 | 14:69.3, 11:66.3 | 11 |
| ANGEL | 25:3.1 | 117919 | NP_653168.2 | 2e-89 | 1:213.2, 14:77.3 | 11 |
| JKAMP | 25:1.4 | 275868 | NP_001092095.1 | 4e-113 | 14:59.9 | 11 |
| DAAM | 96:2.8 | 220475 | NP_055807.1 | 0 | 14:59.6 | 11 |
| LGMN | 96:2.8 | 280591 | NP_005597.3 | 0 | 14:93.2 | 11 |
| FMN | 96:1.4 | 220344 | NP_001096654.1 | 1e-123 | 15:33.0, 1:240.2 | 11 |
| GREM | 96:1.3 | 220377 | NP_071914.3 | 6e-61 | 1:240.6, 15:33.0 | 11 |
| CUL1 | 65:0.2 | 123717 | NP_003583.2 | 0 | 7:148.4 | 13 |
| SOSD | 65:0.5 | 84013 | NP_056279.1 | 2e-20 | 7:16.5 | 16 |
| ETV1 | 65:0.3 | 123714 | NP_004947.2 | 5e-124 | 7:13.9 | 16 |
|  |  |  |  |  |  |  |
| **Ortholog** | **Best Limpet Hit** | **e-value** | **Limpet Loc.** | **Best SeaAnem Hit** | **e-value** | **SeaAnem Loc.** |
| CKDL1/4 | JGI_161601 | 5e-175 | 30:0.4 | XP_001641563.1 | 0 | 3:0.8 |
| MAPK3/5 | JGI_178509 | 0 | 30:0.4 | XP_001641110.1 | 1e-151 | 5:1.6 |
| ISM1/2 | JGI_118957 | 3e-64 | 30:0.4 | N/A | N/A | N/A |
| GPHβ5 | N/A | N/A | N/A | N/A | N/A | N/A |
| GPHα2 | JGI_161605 | 1e-8 | 30:0.5 | N/A | N/A | N/A |
| SAMD4A/B | JGI_119139 | 5e-48 | 30:0.6 | XP_001640702.1 | 1e-65 | 7:2.1 |
| ACTN | JGI_189716 | 0 | 30:0.9 | XP_001633290.1 | 0 | 74:0.5 |
| ANGEL | JGI_118950 | 8e-103 | 30:1.5 | XP_001632273.1 | 2e-66 | 89:0.5 |
| JKAMP | JGI_119100 | 4e-110 | 30:2.2 | XP_001636478.1 | 4e-113 | 38:0.3 |
| DAAM | JGI_159871 | 2e-133 | 22:1.4 | XP_001624968.1 | 8e-140 | 296:0.1 |
| LGMN | JGI_115714 | 1e-147 | 22:1.6 | XP_001624969.1 | 8e-174 | 296:0.2 |
| FMN | JGI_159891 | 9e-124 | 22:1.7 | XP_001637929.1 | 2e-68 | 26:0.5 |
| GREM | JGI_88176 | 3e-144 | 22:1.7 | ABF06564.1 | 2e-29 | 39:1.1 |
| CUL1 | JGI_215748 | 0 | 29:1.6 | XP_001629633.1 | 0 | 138:0.3 |
| SOSD | JGI_88167 | 2e-20 | 22:2.1 | XP_001629632.1 | 9e-18 | 138:0.3 |
| ETV1 | JGI_115677 | 7e-73 | 22:3.2 | XP_001629613.1 | 4e-67 | 138:0.3 |
